# ERAP1 Activity Modulates the Immunopeptidome but Also Affects the Proteome, Metabolism, and Stress Responses in Cancer Cells

**DOI:** 10.1016/j.mcpro.2025.100964

**Published:** 2025-04-04

**Authors:** Martha Nikopaschou, Martina Samiotaki, Elli-Anna Stylianaki, Kamila Król, Paula Gragera, Aroosha Raja, Vassilis Aidinis, Angeliki Chroni, Doriana Fruci, George Panayotou, Efstratios Stratikos

**Affiliations:** 1 National Centre for Scientific Research Demokritos, Agia Paraskevi, Greece; 2 Department of Chemistry, National and Kapodistrian University of Athens, Zografou, Greece; 3 Biomedical Sciences Research Center “Alexander Fleming”, Institute for Bioinnovation, Vari, Greece; 4 Biomedical Sciences Research Center “Alexander Fleming”, Institute for Fundamental Biomedical Research, Vari, Greece; 5 Department of Pediatric Hematology and Oncology, Bambino Gesù Children’s Hospital, IRCCS, Rome, Italy; 6 Center for Translational Immunology, University Medical Center Utrecht, Utrecht University, Utrecht, The Netherlands

**Keywords:** aminopeptidase, antigen processing and presentation, cancer immunotherapy, cellular stress, metabolism

## Abstract

Endoplasmic reticulum (ER) aminopeptidase 1 (ERAP1) metabolizes peptides inside the ER and shapes the peptide repertoire available for binding to major histocompatibility complex class I molecules (MHC-I). However, it may have additional effects on cellular homeostasis, which have not been explored. To address these questions, we used both genetic silencing of ERAP1 expression as well as treatment with a selective allosteric ERAP1 inhibitor to probe changes in the immunopeptidome and proteome of the A375 melanoma cancer cell line. We observed significant immunopeptidome shifts with both methods of functional ERAP1 disruption, which were distinct for each method. Both methods of inhibition led to an enhancement, albeit slight, in tumor cell killing by stimulated human peripheral blood mononuclear cells and in significant proteomic alterations in pathways related to metabolism and cellular stress. Similar proteomic changes were also observed in the leukemia cell line THP-1. Biochemical analyses suggested that ERAP1 inhibition affected sensitivity to ER stress, reactive oxygen species production, and mitochondrial metabolism. Although the proteomics shifts were significant, their potential in shaping immunopeptidome shifts was limited since only 9.6% of differentially presented peptides belonged to proteins with altered expression and only 4.0% of proteins with altered expression were represented in the immunopeptidome shifts. Taken together, our findings suggest that modulation of ERAP1 activity can generate unique immunopeptidomes, mainly due to altered peptide processing in the ER, but also induce changes in the cellular proteome and metabolic state which may have further effects on tumor cells.

## Highlights

• ERAP1 shapes the immunopeptidome of cancer cells.
• ERAP1 silencing or chemical inhibition can also affect the proteome of cancer cells.
• ER stress, ROS production, and mitochondrial metabolism are also affected.
• Immunopeptidome shifts are largely independent of proteome effects.
• ERAP1 activity has secondary effects on cellular metabolic homeostasis.

## Graphical Abstract

**Figure:**
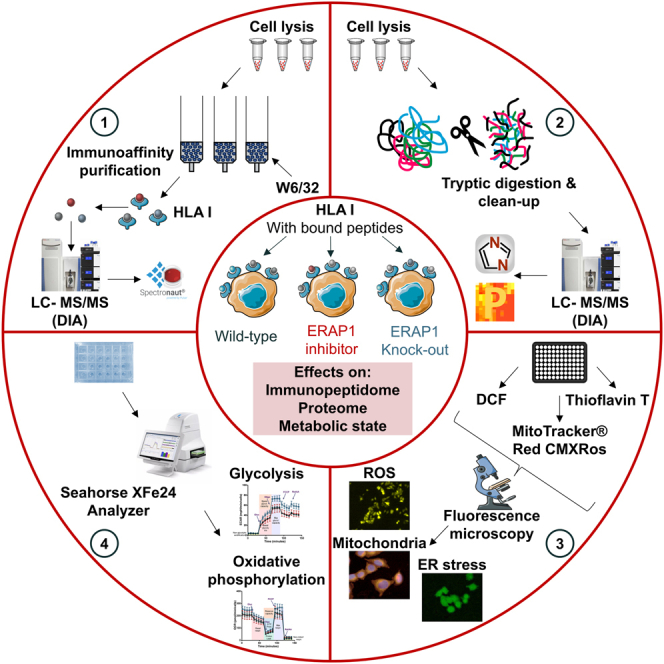

## In Brief

ERAP1 is an intracellular enzyme that regulates the generation of antigenic peptides and adaptive immunity responses. Modulation of ERAP1 activity can generate unique immunopeptidomes in cancer cells, mainly due to altered peptide processing in the ER, but also induce changes in the cellular proteome and metabolic state which may have further effects on tumor cells.

The ability of viruses and tumors to hide within autologous cells poses a major challenge for the immune system. To overcome this challenge, the immune system has developed the major histocompatibility complex class I (MHC-I) antigen presentation pathway, which is active in almost all cell types (1, 2). In this pathway, MHC-I molecules bind fragments of intracellular proteins and transport them to the cell surface (2, 3). There, the peptide-MHC-I complexes are presented to cytotoxic T lymphocytes, which can distinguish between native and foreign proteins and kill cells expressing viral proteins or tumor antigens (1, 2, 3, 4, 5). Natural killer cell responses can also be regulated by MHC-I cargo and complement these effects (6). For effective immune surveillance, a broad peptide repertoire is typically presented (5). This repertoire is collectively known as the immunopeptidome (7).

The generation of the immunopeptidome usually begins with the degradation of intracellular, often defective (8), proteins by the proteasome. The peptide products of this degradation are delivered to the endoplasmic reticulum (ER) via the transporter associated with antigen processing (TAP) (9). Peptides generated by the proteasome are between 3 and 22 amino acids long, while only about 15% correspond to the optimal length for MHC-I binding (8–9 amino acids). Another 15% are longer than 10 amino acids and could serve as peptide precursors for loading on MHC-I (10). These precursors can further be trimmed to the optimal length by the ER-resident aminopeptidases, ERAP1 and ERAP2 (11, 12, 13, 14). Trimmed peptides can be loaded onto MHC-I molecules with the help of the peptide-loading complex (PLC), a multiprotein machinery consisting of the MHC-I together with TAP, tapasin, protein disulfide isomerase family A member 3 (PDIA3), and calreticulin (CALR) (15). The peptide-loaded MHC-I are finally transported to the cell surface via the Golgi complex (4, 9).

Given the importance of the immunopeptidome in adaptive immunity, several efforts are underway to better understand how it is generated and how it can be modulated for therapeutic applications in the context of cancer, autoimmunity, and infections (7). Among others, modulation of the immunopeptidome can be achieved by perturbations in the activity or expression of proteins in the antigen processing and presentation pathway (16), including ERAP1 and its murine homolog ERAAP (17, 18, 19, 20).

Modulation of ERAP1 activity can be induced *in vitro* in two different ways: genetic knockout of the enzyme or pharmacological modulation. The development of ERAP1 inhibitors has been ongoing for several years, and many of the efforts have focused on the active site of the enzyme which contains a zinc atom (21, 22). However, as the active site is largely conserved among other family members, active-site inhibitors often suffer from limited selectivity (23). An alternative approach that has been gaining traction recently is the targeting of ERAP1 allosteric sites with compounds that display enhanced selectivity (23, 24).

In addition to MHC-I antigen processing and presentation, ERAP1 has been suggested to participate in other biological functions, including blood pressure regulation (25, 26), angiogenesis (27), ectodomain shedding of cytokine receptors (28, 29), Hedgehog-dependent tumorigenesis (30), innate immunity (31, 32) and ER stress (33). Since indirect proteomic alterations can contribute to changes in the immunopeptidome (34), ERAP1 could also affect the immunopeptidome indirectly, through its effect in some of the aforementioned functions. At the same time, potential effects of ERAP1 in pathways other than antigen processing and presentation may also have therapeutic potential. Consequently, it is essential to simultaneously evaluate the effects of ERAP1 inhibition on the immunopeptidome and overall cellular functions. This can be performed in an unbiased way by evaluating the changes in the cellular proteome after ERAP1 modulation, as proteins are effectors of biological function (35).

To better understand the potential interplay between direct and indirect regulation of the immunopeptidome by ERAP1, as well as explore its role in cellular homeostasis, we combined immunopeptidomic with proteomic and biochemical analyses in the melanoma cell line A375, a well-established cellular system for exploring immunopeptidome modulation (18). We used a data-independent acquisition (DIA) strategy to analyze how the immunopeptidome and proteome of A375 cells are altered after genetic or pharmacological ERAP1 inhibition, utilizing a recently developed allosteric inhibitor (24). We demonstrate that although ERAP1 activity is critical for immunopeptidome regulation, its function also impacts cellular homeostasis and its activity can induce changes related to mitochondrial metabolism and ER stress. Our data suggest that ERAP1 activity, while highly specialized for antigen presentation, is also important for ER peptide homeostasis and may therefore play a dual role in metabolic adaptations of tumor cells attempting to evade the innate and adaptive immune response.

## Experimental Procedures

### Cell Culture

W6/32 hybridoma cell line (HB-95, American Type Culture Collection), A375 cells (CRL-1619, American Type Culture Collection), and A375 cells after ERAP1 KO (previously generated in-house (19)) were cultured in Dulbecco's modified Eagle medium (DMEM, Biowest, L0104) with the addition of 10% fetal bovine serum (FBS, Biowest, S1810), 2 mM L-glutamine (Biowest, X0550), and 1% Penicillin-Streptomycin (Biowest, L022) at 37 °C, 5% CO_2_. The generation of ERAP1 KO clones has been described before (19). A Western blot analysis confirming the absence of ERAP1 is shown in Supplemental Fig. S1. The immunopeptidome and proteomic analysis of A375 ERAP1 KO cells was performed with clone 1G5. However, later passages of this clone presented gradually unexpected behavior and morphology. Genome-wide SNP array analysis (36) of the 1G5 clone revealed a partial duplication in 1p35 for about 20% of the cells (Supplemental Fig. S2), which however was not reflected in the proteomics results. Subsequently, downstream experiments were performed with clone 1B12 which did not show any genomic instability (Supplemental Fig. S2). THP-1 WT (TIB-202, American Type Culture Collection) and THP-1 ERAP1 KO cells, generated in Dr Doriana Fruci’s lab at Ospedale Pediatrico Bambino Gesù (Król *et al*, manuscript in preparation), were cultured in Rosswell Park Memorial Institute 1640 (Biowest, L0501) with the addition of 10% FBS (Biowest, S1810), 2 mM L-glutamine (Biowest, X0550), and 1% Penicillin-Streptomycin (Biowest, L022) as usual at 37 °C, 5% CO_2._

### Inhibitor Treatment

For the immunopeptidomics, proteomics and surface MHC-I expression analyses, WT cells were treated either with 10 μΜ ((4-methoxy-3-(N-(2-(piperidin-1-yl)- 5-(trifluoromethyl)phenyl)sulfamoyl)benzoic acid)—hereafter named as compound 3 (24)- in complete medium (0.1% dimethyl sulfoxide [DMSO]) or 0.1% DMSO for 6 days at 37 °C, 5% CO_2_. KO cells (A375 KO:1G5 and THP-1 KO) were cultured in the presence of 0.1% DMSO under the same conditions. During the treatment, the medium was refreshed once. At the end of the treatment, cells were harvested and either subjected to flow cytometric analysis or stored at −80 °C until needed for immunopeptidome and proteome isolation. For the rest of the experiments, A375 WT and KO (1B12) were treated similarly but the duration of the treatment was reduced to 48 h.

### Preparation of Immunoaffinity Columns

The preparation of immunoaffinity columns was performed as previously described (18, 19, 37). Briefly, the W6/32 mAb was collected from the HB-95 hybridoma after 5 days of culture in serum-free medium and purified by affinity chromatography using Protein G Sepharose 4 Fast Flow (Cytiva, 17061801). The purified antibody was dialyzed overnight in coupling buffer (NaHCO_3_ 0.1 M, NaCl 0.5 M, pH 8.3). For each column 0.285 g of dry cyanogen bromide activated Sepharose 4B beads (GE Healthcare 17-0430-01) were used. The beads were activated by 1 mM HCl, washed with coupling buffer and mixed with 2 mg of purified antibody per column. The beads with the antibody were rotated overnight at 4 °C for coupling. After coupling, beads were washed with coupling and blocking buffer (Tris–HCl 0.1 M, pH 8.0) and blocked for 3 h at room temperature. Ultimately, coupled beads were washed with three cycles of acidic (CH_3_COONa 0.1 M, NaCl 0.5 M, pH 4.0) and basic (Tris–HCl 0.1 M, NaCl 0.5 M, pH 8.0) buffer and equilibrated with 20 mM Tris–HCl, pH 7.5, 150 mM NaCl. Precolumns were prepared in a similar manner with the omission of the W6/32 coupling step.

### Isolation of MHC-I Immunopeptidome

The isolation of the MHC-I molecules from A375 cells has been previously described (18, 19). Cell pellets (3–5∗10^8^ cells/sample) from WT, inhibitor-treated, and KO cells (two biological replicates) were thawed on ice and mixed with 20 ml lysis buffer each (Tris–HCl, pH 7.5, 150 mM NaCl, 0.5% IGEPAL CA-630, 0.25% sodium deoxycholate, 1 mM EDTA, pH 8.0, complete ULTRA EDTA-free protease inhibitor cocktail tablets (Roche, 4134490)) for 1 h at 4 °C (under rotation). The cell lysate was cleared with ultracentrifugation at 100,000*g* for 1 h at 4 °C and then loaded on the precolumns and the columns. The flow-through from this procedure was loaded on the columns three more times before washing the columns with 20 bed volumes 20 mM Tris–HCl, pH 8.0, 150 mM NaCl, 20 bed volumes 20 mM Tris–HCl, pH 8.0, 400 mM NaCl, 20 bed volumes 20 mM Tris–HCl, pH 8.0, 150 mM NaCl, and finally with 40 bed volumes 20 mM Tris–HCl, pH 8.0. The peptide–MHC-I complexes were eluted by washing with 1% TFA.

Elution fractions 1 to 3 from the above procedure were merged and further purified using reversed-phase C18 disposable spin columns (Pierce, 89870). Briefly, columns were activated with 50% acetonitrile (ACN) and equilibrated with 5% ACN, 0.1% formic acid (FA). Sample composition was adjusted by adding ACN to a final concentration of 5% and samples were loaded sequentially (150 μl/time) followed by centrifugation (1500*g*, 1 min). Each column was washed with 5% ACN, 0.1% FA, and peptides were eluted by washing two times with 30 μl of elution solution (30% ACN, 0.1% FA). Due to the presence of β-2 microglobulin in the flow-through (detected by Western blot), the initial eluates were diluted to 5% ACN and the procedure was repeated. The flow-throughs from the C18 column preparations were subjected to Speed-Vac, reconstituted in 5% ACN, 0.1% FA, and the C-18 purification was repeated as described above. These samples were analyzed by liquid chromatography tandem mass spectrometry (LC-MS/MS) separately.

The purified peptides were further processed by the Sp3 protocol for peptide clean-up (38). Twenty micrograms of beads (1:1 mixture of hydrophilic and hydrophobic Sera-Mag carboxylate-modified beads, GE Life Sciences) were added to each sample in 95% ACN. Peptide clean-up was performed on a magnetic rack. The beads were washed two times with 100% ACN. Peptides were solubilized in the mobile phase A (0.1% FA in water) and sonicated.

### Isolation of the Proteome

Cell pellets were mixed with 150 μl of solution containing 4% SDS, 100 mM Tris/HCl pH 7.6, 0.1 M DTT and incubated at 95 °C for 3 min. DNA was sheared by sonication to reduce the viscosity of the sample and then the lysate was clarified by centrifugation at 13,000*g* for 5 min.

The protein extracts were treated with 200 mM iodoacetamide to alkylate reduced cysteine residues and processed according to the Sp3 protocol (38). Twenty micrograms of beads (1:1 mixture of hydrophilic and hydrophobic SeraMag carboxylate-modified beads; GE Life Sciences) were added to each sample in 50% ethanol. Protein clean-up was performed on a magnetic rack. The beads were washed twice with 80% ethanol followed by one wash with 100% ACN. The beads-captured proteins were digested overnight at 37 °C with 0.5 μg trypsin mix in 25 mM ammonium bicarbonate under vigorous shaking (1200 rpm, Eppendorf Thermomixer). The supernatants were collected, and the peptides were purified by a modified Sp3 clean-up protocol and finally solubilized in the mobile phase A (0.1% FA in water), and sonicated. Peptide concentration was determined through absorbance measurement at 280 nm using a nanodrop instrument.

### Proteomics Experimental Design and Statistical Rationale

Proteomics and immunopeptidomics samples were run on an LC-MS/MS setup consisting of a Dionex Ultimate 3000 nano RSLC online with a Thermo Q Exactive HF-X Orbitrap mass spectrometer. Peptidic samples were directly injected and separated on a 25 cm-long analytical C18 column (PepSep, 1.9 μm^3^ beads, 75 μm ID) using a 30 min long run for immunopeptidomics and a 1-h long run for proteomics, starting with a gradient of 7% buffer B (0.1% FA in 80% ACN). For the immunopeptidomics samples, 35% B was reached in 16.5 min, followed by an increase to 45% in 1.5 min and a second increase to 99% in 0.5 min. Gradient was then kept constant for 1min before returning to 7% over 10.5 minutes. For the proteomics samples the increase to 35% B was achieved over 40 min, followed by a rise to 45% in 5 min, and then to 99% in 0.5 min. The gradient was ultimately maintained at 99% for 14.5 min to allow for equilibration. A full mass spectrum was acquired in profile mode using a Q Exactive HF-X Hybrid Quadropole-Orbitrap mass spectrometer, operating in the scan range of 375 to 1400 *m/z* using 120K resolving power with an automatic gain control of 3 × 10^6^ and max injection time of 60 ms, followed by data-independent analysis using 8 Th windows (39 loop counts) with 15K resolving power with an automatic gain control of 3 × 10^5^, max injection time of 22 ms, and a normalized collision energy of 26.

Immunopeptidomic analysis included two biological replicates per condition (WT, inhibitor-treated, and ERAP1 KO cells), each analyzed three times (technical replicates), while a blank sample was also included to increase robustness of peptide identification. Immunopeptidomics mass spectra files were searched in library-free mode against the nuORFdb v1.0 database (39) (323,848 protein sequences, retrieved: 04-03-2024) in Spectronaut (Biognosys, version 19) using “unspecific” search, with N-terminal acetylation and methionine oxidations as variable modifications and false discovery rate (FDR) at 5% at the peptide spectrum match and peptide level. Spectronaut’s default settings were chosen for the rest of the parameters (Supplemental Methods). In cases where a peptide was matched to multiple proteins, priority was given to canonical proteins. Comparison between the three conditions was performed with an unpaired *t* test in Spectronaut. Two LC-MS/MS runs and searches were performed, as indicated above. Peptides with log2 difference above or equal to 1, q value below or equal to 0.05 and with similar behavior in the two LC-MS/MS runs were considered differentially expressed.

Proteomics analysis included four biological replicates for A375 and three biological replicates for THP-1 cells per experimental condition (*i.e.*, WT, inhibitor-treated, and ERAP1 KO cells). Raw data were analyzed in DIA-NN 1.9.1 (Data-Independent Acquisition by Neural Networks**)** (40) against the reviewed human UniProt protein database (20699 proteins, retrieved 24-11-2023). Search parameters were set to allow up to two possible trypsin enzyme missed cleavages. A spectra library was generated from the DIA runs and used to reanalyze them. Cysteine carbamidomethylation was set as a fixed modification, while N-terminal acetylation and methionine oxidations were set as variable modifications. The match between runs feature was used for all the analyses and the output (precursor) was filtered at 0.01 FDR. The protein inference was performed at the gene level using only proteotypic peptides. The double pass mode of the neural network classifier was also activated. Downstream data analysis was performed with the Perseus software v. 1.6.15 (https://maxquant.net/perseus/) (41). Briefly, data were log transformed, identifications with less than 70% valid values in at least one experimental group were removed and missing values were imputed from a normal distribution. For the determination of differentially expressed proteins, Hawaii plots were generated (Pearson correlation, 100 permutations, class A: S0 = 0.1, FDR = 0.05, class B: S0 = 0.5, FDR = 0.05). Proteins passing the class B classification were considered as differentially expressed. Annotated spectra of differentially expressed proteins with a single peptide identification were visualized with Skyline-daily 23.1.1.520 (https://skyline.ms/project/home/software/Skyline/begin.view) (42).

### Isolation of PBMCs

PBMCs were isolated from a healthy donor according to standard procedures (https://www.bcchr.ca/CAN-ASC/protocols). All authorisations/clearances for the use of PBMCs isolated from healthy donors have been obtained and approved by the ethics committee of Bambino Gesù Children’s Hospital, IRCCS, Rome, Italy. Blood was diluted 1:1 with PBS and laid on top of equal amount of Pancoll density gradient medium (Pan Biotech, P04-60500). Samples were centrifuged for 20 min at 1200*g* without brakes, the buffy coat was collected and washed twice with PBS. Cells were either cryopreserved or cultured in 6-well plates at a density of 2,000,000 cells/ml in complete Roswell Park Memorial Institute Medium medium with 100 units/ml interleukin-2.

### Caspase-3/7 Assay

For the caspase-3/7 apoptosis assay, A375 cells were seeded at a density of 20,000 cells/ml in a 24-well plate and cocultured with PBMCs prestained with CellTracker Deep Red (Invitrogen) at an effector-target ratio 10:1 for 4 h in the presence of inhibitor or DMSO. Next, CellEvent Caspase-3/7 Green Detection Reagent (Thermo Fisher Scientific) was added and apoptotic cells were detected with Leica DMi8 fluorescence microscope (Leica Microsystems) and quantified with ImageJ (https://imagej.net/ij/) (43). Statistical significance was evaluated by one-way ANOVA, followed by Dunnett’s multiple comparisons test in GraphPad Prism v.8 (www.graphpad.com).

### PBMCs Cytotoxicity Assay

A375 cells were cocultured with PBMCs prestained with CellTracker Deep Red (Invitrogen) at an effector-target ratio 10:1 for 16 h in the presence of inhibitor or DMSO. After the coculture, samples were collected, washed, and resuspended in 200 μl medium. Prior to acquisition, 50 μl of Count Bright Absolute Counting Beads (C36950, Thermo Fisher Scientific) along with propidium iodide at a final concentration of 5 μg/ml were added. Data acquisition was performed using a BD LSRFortessa flow cytometer (BD Bioscience), and the data were analyzed with FlowJo software (FlowJo, LLC, version 10.10.0, BD Life Sciences https://www.flowjo.com/). Statistical significance was evaluated by one-way ANOVA, followed by Dunnett’s multiple comparisons test in GraphPad Prism v.8.

### Dichlorofluorescein Assay

For the dichlorofluorescein (DCF) assay A375 cells were seeded in 96-well black wall, transparent flat-bottom plates at a density of 5000 cells/well (100 μl/well). Cells were exposed to either 0.1% DMSO or compound 3, as described above. Forty eight hours later the medium was removed, cells were washed one time with PBS and exposed to either 10 or 25 μΜ 2',7'-dichlorodihydrofluorescein diacetate (H2DCFDA) and Hoechst 33342 1 μg/ml for 45 min at 37 °C, 5% CO_2._ At the end of the incubation period, the fluorescent probes containing medium was removed, cells were washed twice with PBS and once with Fluorobrite DMEM and resuspended in Fluorobrite DMEM. Wells exposed to 25 μΜ H2DCFDA were used for fluorescence measurement with a BioTek Synergy H1 plate reader (DCF: ex. 485 nm, em. 535 nm/Hoechst: ex. 352 nm, em. 454 nm). Ten micromolars were used for taking pictures at a Cytation-5 instrument, equipped with filters for yellow fluorescence protein (ex. 500, em. 542) and 4',6-diamidino-2-phenylindole (DAPI) (ex. 377, em. 477). DCF fluorescence was normalized to Hoechst fluorescence (Synergy H1) or number of nuclei (Cytation 5) and results are expressed as percentage of the WT cells.

### Thioflavin-T Assay

For the thioflavin-T (ThT) assay, A375 cells were seeded in a 6-well flat-bottom transparent plate at a density of 75,000 cells/ml (2 ml/well) and treated as described in the inhibitor treatment paragraph above. At the end of the treatment period, the stock ThT reagent (5 mM) was prepared fresh as described in (44) in Fluorobrite DMEM with 10% FBS, 1% L-glutamine, and 1% sodium pyruvate. The medium was then aspirated; cells were washed one time with PBS and were then treated with 5 μΜ ThT in complete Fluorobrite DMEM with or without 5 mΜ DTT for 40 min at 37 °C, 5% CO_2._ ThT fluorescence was observed with a Nikon Eclipse Ts2R-FL fluorescence microscope, equipped with a C-LED385∗ filter (Ex 390/38, BA 475/90). The obtained pictures were preprocessed using Fiji (background subtracted, thresholded) (https://fiji.sc/) (45). Total intensity was normalized with the area. Results are expressed as percentage of the WT cells without DTT.

### Mitotracker Assay

For the Mitotracker assay A375 cells were seeded in a 96-well black wall, transparent flat-bottom plate at a density of 5000 cells/well (100 μl/well). Cells were exposed to either 0.1% DMSO or compound 3, as described above. At the end of the treatment, cells were washed one time with PBS and exposed to 200 nM Mitotracker Red CMXRos probe (Invitrogen), diluted in Fluorobrite DMEM with 1% L-glutamine and 1% sodium pyruvate for 15 min at 37 °C, 5% CO_2._ Postexposure to the probe, the cells were fixed with 4% paraformaldehyde in PBS, stained with DAPI and fluorescence images were taken at a at a Cytation-5 instrument, equipped with filters for Texas Red (ex. 586, em. 647) and DAPI (ex. 377, em. 477). Mitotracker fluorescence was normalized to number of nuclei, and results are expressed as percentage of the WT cells.

### Extracellular Flux Analysis

For the extracellular flux analysis A375 cells were seeded in a Seahorse XF analyzer 24-well plate and incubated for 48 h with 0.1% DMSO or compound 3 at 37 °C, 5% CO_2_. Subsequently, cells were incubated for 1 h in a non-CO_2_ incubator in DMEM-based assay medium, supplemented with 2 mM glutamine and lacking glucose, pyruvate, sodium bicarbonate, Hepes, and phenol red. The assay included four injections of 10 mM glucose, 1.5 μM oligomycin, 1 μM trifluoromethoxyphenylhydrazone, and 0.5 μM of each of rotenone and antimycin A. Measurements of oxygen consumption rate (OCR) and extracellular acidification rate (ECAR) were collected throughout the assay. Upon completion of the assay, cells were fixed with 4% paraformaldehyde and stained with DAPI. Normalization was performed by counting nuclei stained with DAPI and results are shown for OCR and ECAR as pmol O_2_/min/cells and mpH/min/cells, respectively.

## Results

### The Immunopeptidome of A375 Melanoma Cells is Responsive to Downregulation of ERAP1 Activity

To investigate the changes in the immunopeptidome of cancer cells due to ERAP1 inhibition, we compared MHC-I peptide repertoires in A375 melanoma cells under three conditions: untreated, treated for 6 days with 10 μM of an allosteric ERAP1 inhibitor (compound 3, targeting the B3P site (24), Supplemental Fig. S3), and an ERAP1-deficient clone (19). The inhibitor was used at a noncytotoxic concentration (Supplemental Fig. S4). A375 cells carry the “GG” ERAP2 allele which is known to lead to nonsense-mediated ERAP2 mRNA decay, leading to very low or complete lack of ERAP2 protein production. This makes this cell line a good model to evaluate ERAP1-dependent antigen presentation (Supplemental Fig. S5). Neither the inhibitor treatment nor the ERAP1 KO significantly affected MHC-I cell surface expression as detected by the pan-MHC antibody W6/32 (Supplemental Fig. S6). To isolate the peptide–MHC-I complexes, A375 cells were lysed and the cleared lysate was subjected to immunoaffinity purification with immobilized W6/32 mAb. Elution took place under acidic conditions and the purified peptides were analyzed by LC-MS/MS. Due to the relatively low number of peptides identified, we repeated the purification using the flow-through fractions from the first C-18 column and analyzed them in a separate LC-MS/MS run. Since antitumor responses can also be targeted against noncanonical peptides, we searched against the Riboseq-based nuORFdb v1.0 (39). In total, 734 unique peptides between 8 and 16 amino acids were identified in all the conditions, after removal of peptides detected in a blank run (Supplemental Table A). Due to the relatively low number of identified peptides and to validate the robustness of our isolation procedure, we used MHC_Motif_Decon 1.0 to analyze identified peptides against the expected alleles carried by A375 cells (HLA-A∗01:01, A∗02:01, B∗44:03, B∗57:01, C∗06:02, and C∗16:01). Our analysis confirmed that 95% of the peptides between 9 and 14 amino acids (the range analyzed by the tool) matched the expected motifs (Supplemental Fig. S7) (46).

**Fig. 1:**
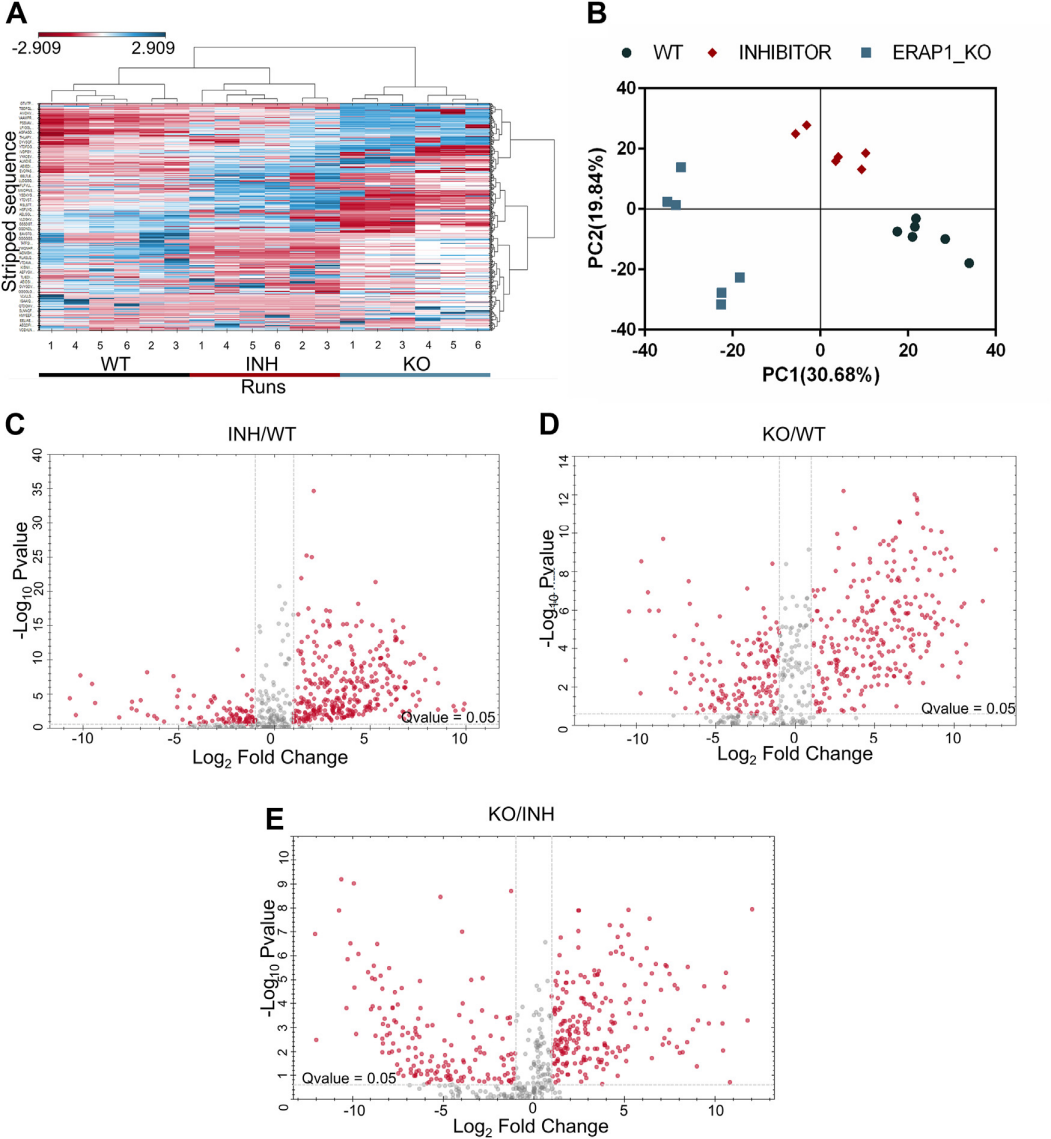
**Analysis of the immunopeptidome of WT, inhibitor-treated, and ERAP1 KO A375 cells.***Panel A*, heat map from LC-MS/MS run 1 showcasing peptide distribution across different experimental conditions. Hierarchical clustering was calculated by Manhattan distance. Colors indicate peptide intensities, ranging from low (*red*) to high (*blue*). (Graph generated with Spectronaut v. 19). *Panel B*, principal component analysis (PCA) from LC-MS/MS run 1. Principal components 1 and 2 contribute to the explanation of 50% of the sample variability. Based on these two PCs, WT samples (*circle*), inhibitor-treated samples (*diamond*), and KO samples (*square*) formed three distinct groups, indicating that the replicates within each group share similarities with each other but there were differences in the immunopeptidomes between different treatments. *Panels C*–*E*, volcano plots from LC-MS/MS run 1 indicating the statistical significance of the differences between (*C*) the inhibitor-treated and the WT A375 cells, (*D*) the genetically modified (KO) and the WT A375 cells, and (*E*) the KO *versus* inhibitor-treated cells. Each *circle* represents a unique peptide sequence. Peptides with a q-value ≤0.05 and a log2 fold change ≥1 are considered statistically significant. ERAP, endoplasmic reticulum aminopeptidase.

Clustering analysis validated the statistical differences between the three conditions (Fig. 1*A* and Supplemental Fig. S8*A*). Principle component analysis indicated discreet clusters for each biological condition, suggesting a good degree of reproducibility between the replicates and discrete patterns for each experimental condition (Fig. 1*B* and Supplemental Fig. S8*B*). Volcano plots comparing the inhibitor-treated A375 cells and ERAP1 KO cells with untreated WT A375 cells are shown in Figure 1, *C* and *D* and Supplemental Fig. S8, *C* and *D*. These results clearly indicate significant immunopeptidome shifts for both methods of ERAP1 functional disruption.

After merging the two sets of identified peptides, 467 peptides were found to be either differentially expressed or unique in the inhibitor-treated cells compared to the control cells (321 peptides significantly upregulated and 146 significantly downregulated, q-value ≤0.05 and log_2_fold change ≥1). Five hundred one peptides were differentially expressed or unique in the KO cells compared to the WT control (263 peptides significantly upregulated and 238 significantly downregulated). In all cases, identified peptide sequences corresponded to sequence motifs consistent with the binding preferences (including anchor residues) of human leukocyte antigen (HLA)-A and HLA-B alleles carried by A375 cells and to a lesser degree for HLA-C alleles (Supplemental Fig. S9).

Interestingly, direct comparison between the inhibitor-treated cells and the ERAP1 KO cells indicated that the immunopeptidome shifts were distinct for each method of functional disruption of ERAP1 activity (Fig. 1*E* and Supplemental Fig. S8*E*). This is likely due to the allosteric nature of the inhibitor and is analogous to previous results obtained with an ERAP1 inhibitor targeting the malate allosteric site (19). This finding reinforces the notion that targeting distinct allosteric sites in ERAP1 may be a viable method for fine-tuning the immunopeptidome for therapeutic applications.

To estimate the binding affinity of the identified peptides to the HLA-I alleles expressed by the A375 cells, we used again MHC_Motif_Decon 1.0 (46) for peptides between 8 and 14 amino acids long, which predicts binding affinity using the NetMHCpan-4.1 server (47).

**Fig. 2:**
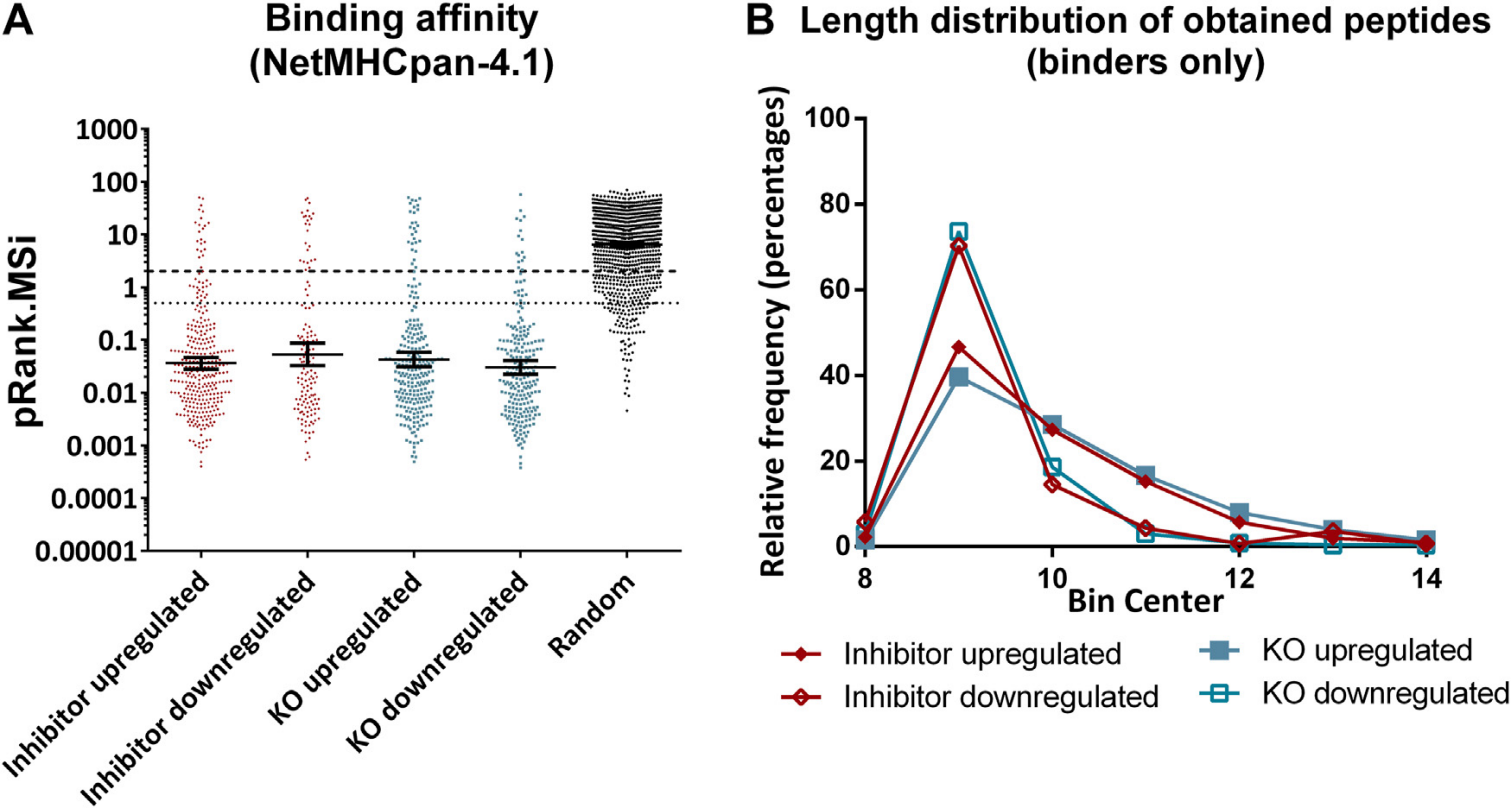
**Binding affinity predictions and length distribution of binders.***Panel A*, distribution of predicted affinities (NetMHCpan-4.1) of the significantly upregulated or downregulated peptides after inhibitor treatment or ERAP1 KO for the HLA alleles present in A375 cells (A∗01:01, A∗02:01, B∗44:03, B∗57:01, C∗06:02, and C∗16:01). Each point on the graph signifies a distinct peptide sequence. Only the score for the top predicted HLA allele is depicted for each peptide. A set of random peptide sequences generated by RandSeq (https://web.expasy.org/randseq/) was also plotted as negative control. Prediction scores below 2 (*dashed line*) indicate binding to at least one of the HLA alleles, with scores below 0.5 (*dotted line*) indicating strong binding. *Panel B*, length distribution of the significantly upregulated or downregulated binding peptides after inhibitor treatment or ERAP1 KO. ERAP, endoplasmic reticulum aminopeptidase; HLA, human leukocyte antigen.

Figure 2*A* shows that most of the peptides are predicted to bind to the HLA-I alleles carried by A375 cells, as opposed to a random set of peptides that was used as a control for predictions. No major differences were observed between conditions, suggesting that ERAP1 functional disruption does not grossly block antigen presentation. However, a substantial shift toward longer length peptides was evident (Fig. 2*B*) in the inhibitor-treated and ERAP1 KO. This is consistent with the inhibition of the peptide-shortening activity of ERAP1. These patterns persisted when analyzing peptides per HLA-allotype, suggesting that effects are not dominated by a specific HLA-allele (Supplemental Figs. S10 and S11). Interestingly, the allosteric inhibitor targeting the B3P site used here affected peptide length almost as much as the ERAP1 KO, in contrast to the effect of a previously analyzed allosteric inhibitor targeting the malate allosteric site (19). This finding suggests that different allosteric sites in the internal cavity of ERAP1 may play distinct roles in length selection (48).

To better evaluate whether the observed immunopeptidome shifts may translate to changes in the immunogenicity of cells, we predicted the immunogenicity score of the differentially expressed 9mers using the DeepImmuno immunogenicity prediction tool (49). The distribution of the obtained immunogenicity scores indicated that a higher percentage of the presented 9mers upregulated in the KO and inhibitor-treated cells had scores above 0.8, when compared to the downregulated peptides (Supplemental Fig. S12). Moreover, we searched the identified peptides to see if they correspond to known antigenic peptides listed in the Immune Epitope Database (50). We identified several known antigenic peptides (including MAGE antigens and antigenic epitopes listed in the Immune Epitope Database), several of which were upregulated by ERAP1 KO or inhibition (Supplemental Tables S1 and S2). Several antigenic peptides were altered in nonidentical ways between KO or inhibitor-treated cells suggesting that for these antigenic epitopes the two ways of functional disruption may not be equivalent. Interestingly, 2.3% of the identified peptides originated from unannotated proteins (Supplemental Table S3), some of which were upregulated by ERAP1 KO or inhibition. These results suggest that blocking the processing of antigenic peptides by ERAP1 can lead to specific immunopeptidome changes that can promote cellular antigenic responses, and this can include unannotated protein ORFs that can be a source of neoantigenic epitopes.

### ERAP1 Functional Disruption can Enhance the Immunogenicity of Tumor Cells

**Fig. 3:**
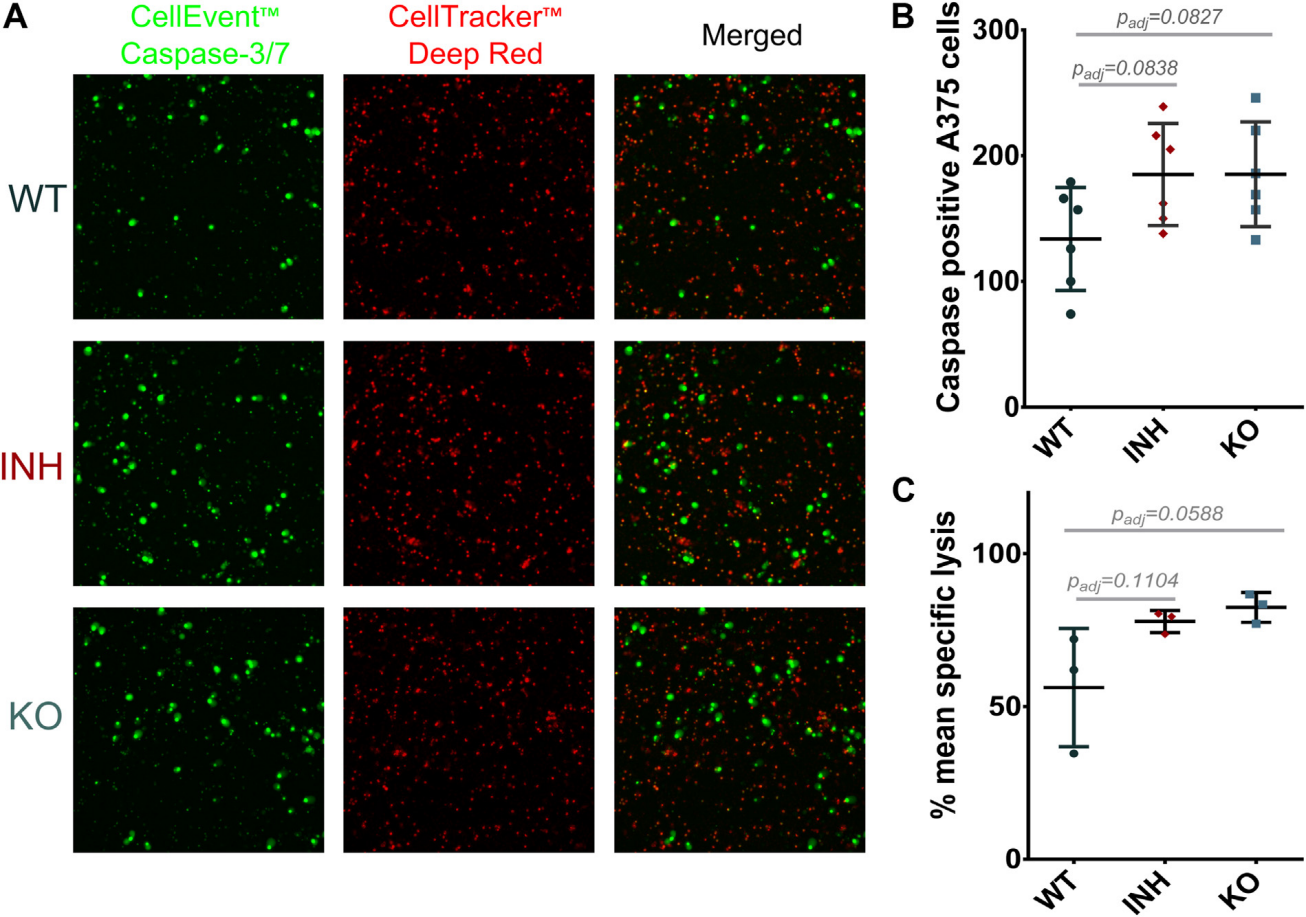
**Immunogenicity analysis of A375 cells.***Panel A*, representative fluorescence microscopy images of activated PBMCs (*red*) and caspase positive cells (*green*) in all conditions. *Panel B*, graph showing the changes in caspase-positive inhibitor-treated (*diamond*) and ERAP1 KO (*square*) A375 cells, compared to A375 WT cells (*circle*). The average, with its respective SD, was calculated from six images per experimental condition. Statistical significance was evaluated by one-way ANOVA, followed by Dunnett’s multiple comparisons test in GraphPad Prism v.8. *Panel C*, graph showing the change in mean-specific lysis in inhibitor-treated and ERAP1 KO cells in the PBMC cytotoxicity assay. The average, with its respective SD, was calculated from three technical replicates per experimental condition. The calculated adjusted *p* values comparing KO cells and inhibitor-treated cells to the WT cells are indicated. Statistical significance was evaluated by one-way ANOVA, followed by Dunnett’s multiple comparisons test in GraphPad Prism v.8. ERAP, endoplasmic reticulum aminopeptidase; PBMC, peripheral blood mononuclear cell.

Immunopeptidome shifts due to ERAP1 inhibition have been theorized to affect the recognition of cancer cells by CD8+ T cells by either enhancing the presentation of rare antigenic epitopes (51), by inducing the presentation of novel epitopes that elicit the generation of new effector T cells (52), or by perturbing the interaction with inhibitory natural killer cell receptors (53, 54, 55). To test whether the immunopeptidome shifts described above can affect cytotoxic responses against this melanoma cell line, we cocultured human PBMCs with WT A375 cells, ERAP1 KO A375 cells, or A375 cells treated with the allosteric inhibitor. Cytotoxic responses of immune cells were evaluated by a fluorescence, microscopy-based, caspase apoptosis imaging assay, and a flow cytometry–based cytotoxicity assay. Compared to WT cells, both assays showed a similar trend of increased killing of inhibitor-treated and ERAP1 KO cells (Fig. 3). Although the cytotoxicity enhancement was mild, it was consistent throughout both methods of ERAP1 functional disruption and both functional assays. The magnitude of functional responses observed was limited, especially compared to previous *in vivo* studies (51, 53). However, it should be noted that this assay can only report on the enhancement of preexisting or cross-reacting cellular responses, as the amplification of T-cell clones is not possible, thus minimizing potential effects. Overall, this result reinforces our confidence that blocking ERAP1 function can contribute to enhanced immunogenicity, even in the absence of combinatorial treatments.

### Functional Disruption of ERAP1 Alters the Proteome of A375 Cells

**Fig. 4:**
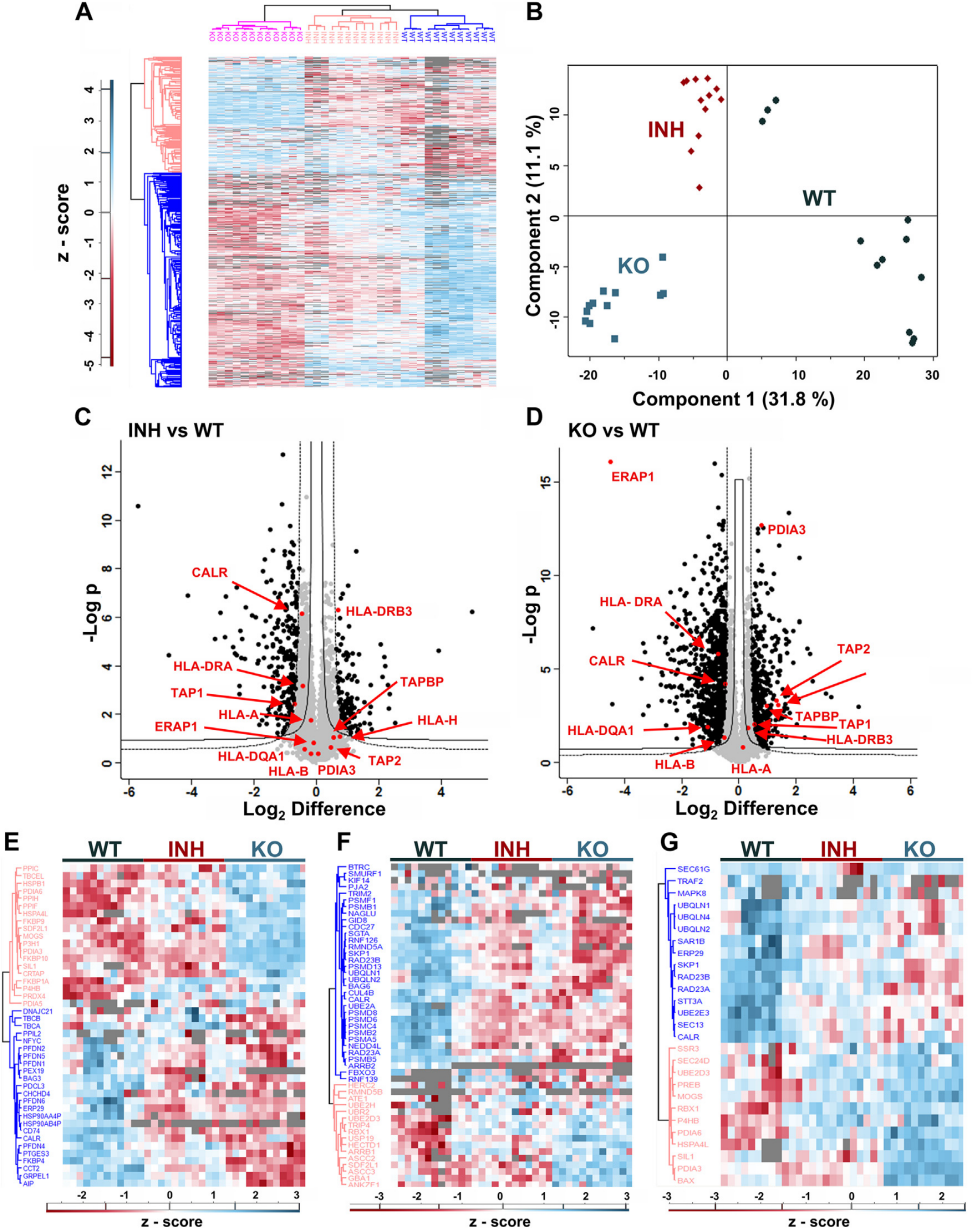
**Proteomic analysis of WT, inhibitor-treated, and ERAP1 KO A375 cells.***Panel A*, heat map showing the cluster analysis and distribution of proteins in the three conditions (WT, inhibitor-treated, and ERAP1 KO A375 cells) for each replicate (four biological replicates, each measured in three technical replicates) (graph generated with Perseus v. 1.6.15). *Panel B*, principal component analysis (PCA) of the three experimental conditions. Each point represents a distinct biological or technical replicate. *Panels C* and *D*, Hawaii plots of identified proteins, indicating the statistical significance of the observed differences in protein abundance between the two treatment conditions (inhibitor-treated and ERAP1 KO) and the WT cells. Three hundred twenty-five proteins in the inhibitor-treated (*C*) and 1095 proteins in the KO cells (*D*) were differentially expressed. Select proteins that participate in antigen presentation are indicated in *red* (graph generated with Perseus v. 1.6.15). *Panels E*, *F*, and *G*, heat maps of proteins participating in protein folding (*E*), proteasomal protein catabolic process (*F*), and protein processing in the endoplasmic reticulum (*G*) (graphs generated with Perseus v. 1.6.15). ERAP, endoplasmic reticulum aminopeptidase.

To examine if ERAP1 activity has significant effects on the proteome of the A375 cell line, we used data-independent analysis to compare the proteomic content of the same conditions we used for the immunopeptidome analysis described above. In total, 12 replicates per experimental condition were analyzed (4 biological replicates, 3 technical replicates for each biological). Overall, we identified 5038 proteins reproduced in at least 70% of the replicates of at least one experimental condition (Supplemental Table B). One thousand one hundred fifty-nine of those proteins were differentially expressed in the inhibitor-treated and ERAP1 KO cells (FDR = 0.05, S0 = 0.5). Based on the identified proteins, the three conditions tested (WT, inhibitor-treated, and KO cells) formed discreet hierarchical clusters (Fig. 4*A*) and grouped in the principal component analysis (Fig. 4*B*), which suggests distinctive features for each experimental condition. Three hundred twenty-five proteins were differentially expressed in the inhibitor-treated cells compared to WT cells (Fig. 4*C*). Seventy nine percent (258 out of 325) of those proteins were also significantly altered similarly in the KO cells. One thousand ninety-five proteins were differentially expressed in the KO cells compared to WT cells (Fig. 4*D*). Although both methods of ERAP1 functional disruption resulted in significant proteome shifts, they were not identical. Indeed, more proteins were both upregulated and downregulated in the KO compared to the inhibitor-treated cells (Supplemental Fig. S13). This could either indicate noncomplete inhibition of ERAP1 by the compound or indirect effects on the proteome due to the lack of the protein in the KO cells. Several components of the antigen presentation pathway were found altered, primarily in the ERAP1 KO cells, such as components of the PLC (PDIA3, TAPBP, CALR, and TAP chain 2), suggesting potential compensatory changes in the antigen loading process due to lack of ERAP1 (Fig. 4, *C* and *D*). Interestingly, we detected changes in abundance for some MHC II alleles (HLA-DRA and HLA-DQA1 for the KO cells and HLA-DRB3 for the inhibitor-treated cells). A potential role for ERAP1 in regulating MHC class II presentation and CD4^+^ responses through the regulation of the quantity of cytoplasmic peptides has been proposed before and could underlie these effects (56). In addition to these, we observed alterations in other related processes, such as protein folding (Fig. 4*E*), proteasomal protein catabolic process (Fig. 4*F*) and protein processing in the ER (Fig. 4*G*).

Changes in the proteome may also affect the composition of the immunopeptidome. Although it is now well established that ERAP1 can regulate the cellular immunopeptidome through its ER trimming activity, its effects on the cellular proteome may also contribute indirectly. Indeed, of the 501 peptides found to be differentially presented in the ERAP1 KO cells, 48 (9.6%) of them belong to proteins that have altered expression levels in the same cells. This suggests that proteomic changes due to ERAP1 KO can affect up to 1/10 of the immunopeptidome. In terms of proteins, however, only 43 of the 1095 proteins (4.0%) that were found to be expressed in different levels in the ERAP1 KO cells, were associated with differentially presented peptides in the immunopeptidome. This may be indicating that the impact of the proteomic changes on the immunopeptidome is limited to a small subset of cellular proteins. These effects were smaller in the case of the ERAP1 inhibitor (3% and 3.4% respectively). Overall, our findings suggest that although ERAP1 activity can influence the cellular proteome and some of those changes can be translated to immunopeptidome shifts, they are likely secondary to the direct effect of ERAP1 on the generation or destruction of antigenic peptides in the ER.

Given the observed proteomic changes upon ERAP1 disruption, we explored if those changes translate to specific biochemical pathways. We used the ShinyGΟ tool (57) to compare between the two treatment conditions. Differentially expressed proteins in each experimental condition were used as input and were compared to all the proteins obtained in our proteomics experiment to identify relevant Kyoto Encyclopedia of Genes and Genomes (58) pathways. The top 25 pathways ordered by fold enrichment are shown in Supplemental Fig. S14. Overall, despite the existence of some unique pathways for each experimental condition, several of the pathways are shared between the inhibitor-treated and KO cells. These include, among others, oxidative phosphorylation, chemical carcinogenesis-reactive oxygen species (ROS), metabolic pathways, and several disease-related pathways.

### ERAP1 Disruption Affects the Proteome of THP-1 Cells

Given the surprisingly large effect of both genetic and pharmacological inhibition of ERAP1 on the proteome of A375 cells, we inquired whether this phenomenon is specific to A375 cells or is more general to other cancer cells. To address this, we analyzed the proteomic changes of THP-1 cells, treated identically to A375. For THP-1 cells we identified 4943 proteins present in at least 70% of the replicates of at least one experimental condition (Supplemental Table B). Eight hundred seventy-seven were differentially expressed in the inhibitor-treated or KO cells, compared to the WT control (FDR = 0.05, S0 = 0.5). Similar to the results obtained for A375 cells, hierarchical clustering and principal component analyses indicated three distinct clusters consistent with distinct proteomic profiles per biological condition (Supplemental Fig. S15, *A* and *B*). Eighty three proteins were differentially expressed in the inhibitor-treated cells and 58 of them were affected in a similar manner in the ERAP1 KO. ERAP1 KO altered the levels of 853 proteins (Supplemental Fig. S15, *C* and *D*). Venn diagrams indicate that despite the smaller effect of the inhibitor compared to the KO cells, there are several shared changes in the significantly upregulated and downregulated proteins between the two treatment conditions (Supplemental Fig. S15, *E* and *F*).

By comparing the proteomic changes observed in the A375 and THP-1 cell lines, we identified common patterns in both cell lines, although the overall magnitude of changes was smaller in THP-1 cells. In particular, some components of the PLC were similarly affected. For example, the TAP chain 2, a component of the TAP complex responsible for the transport of peptides from the cytosol to the ER (59), was found upregulated in both A375 and THP-1 ERAP1 KO cells (Fig. 4*D* and Supplemental Fig. S15*D*). Tapasin (TAPBP) and CALR followed a similar trend. Furthermore, in both cellular systems ERAP1 KO led to significant alterations in the levels of some HLA (HLA-II alleles on both cell lines and HLA-B on THP-1 cells). Finally, a closer look in processes affected in A375 cells (protein folding, proteasomal catabolic processes, protein processing in the ER, oxidative phosphorylation, and response to ROS) indicated that several proteins that belong to these processes were also affected in THP-1 cells (Supplemental Fig. S16). Overall, the observed effect on THP-1 cells was smaller in magnitude than on A375 cells. This may be due to the differences in the ERAP1 alleles carried by the 2 cell lines. Based on our previous analysis, A375 cells carry allotypes 1 and/or 2 (18), whereas THP-1 cells carry one allele with allotype 1 or 2, combined with an allele carrying SNPs that form one of allotypes 4 to 8 (Supplemental Table S4 and Supplemental Fig. S17). Allotypes 1 and 2 have been proposed to be “hyperactive,” suggesting that the basal ERAP1 activity in A375 cells may be higher than in THP-1 cells (60). Taken together, our analyses suggest that the effects on the proteome of cancer cells induced by ERAP1 inhibition or ERAP1 KO are not limited to the A375 cell line but may extend to other cancer cell types as well.

### ERAP1 Inhibition Reduces ROS in A375 Cells

**Fig. 5:**
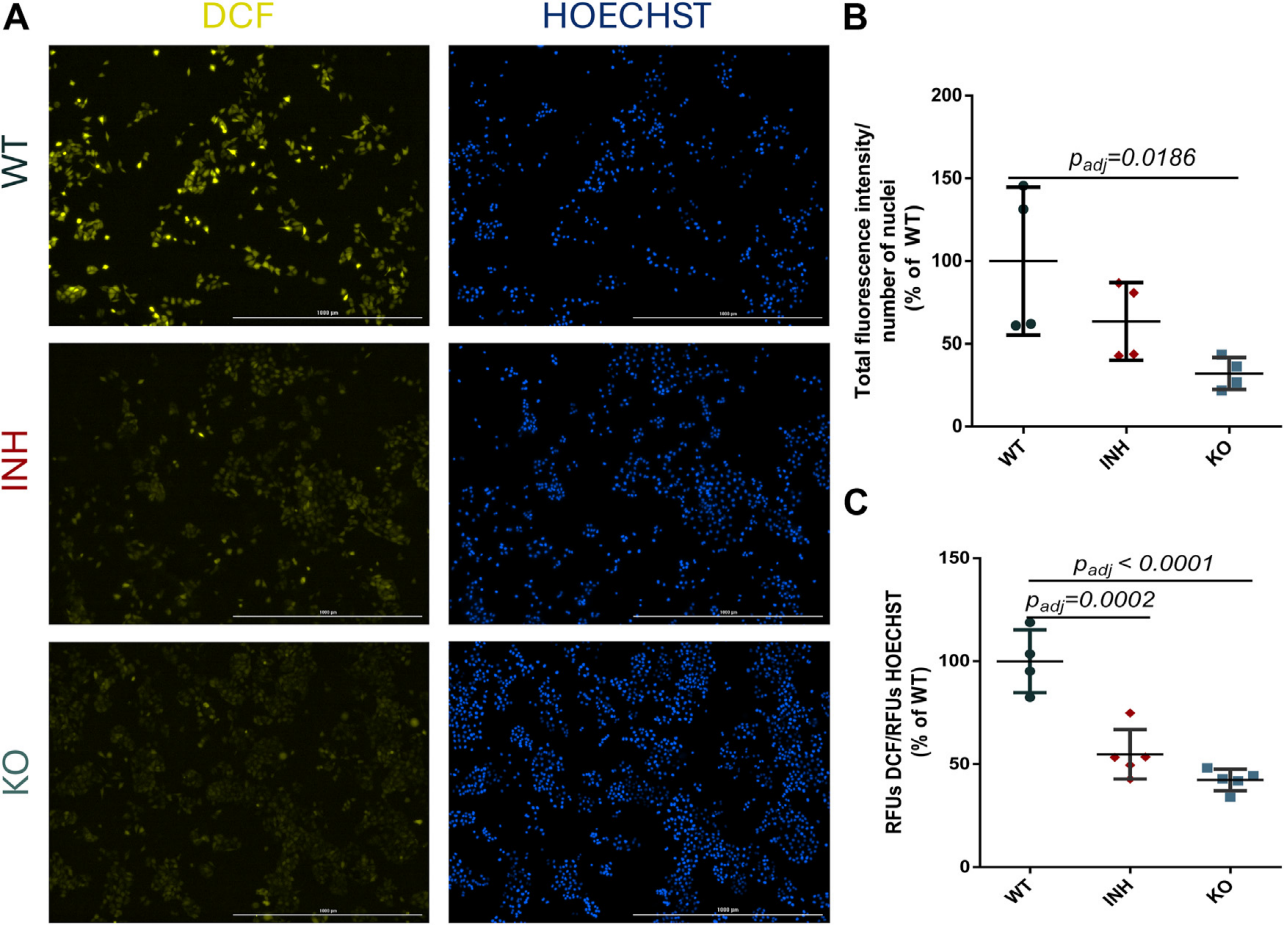
**Dichlorofluorescein assay for measurement of reactive oxygen species.***Panel A*, representative images of WT, inhibitor-treated (INH), and ERAP1 KO A375 cells incubated with H2DCFDA (*left*) or Hoechst 33342 (*right*). *Panel B*, total fluorescence intensity per number of nuclei quantitated by direct cell imaging on a Cytation-5 instrument for the three biological conditions. *Panel C*, relative DCF fluorescence units (RFU) normalized to Hoechst dye fluorescence measured by direct fluorescence measurement on a BioTek Synergy H1 plate reader. The calculated adjusted *p* values comparing KO cells and inhibitor-treated cells to the WT cells are indicated. Statistical significance was evaluated by one-way ANOVA, followed by Dunnett’s multiple comparisons test in GraphPad Prism v.8. ERAP, endoplasmic reticulum aminopeptidase.

Since the proteomics results and the subsequent pathway analysis (Supplemental Figs. S14 and S18) suggested that ERAP1 function may relate to changes in cellular stress and ROS, we used the H2DCFDA dye to assess the activity of ROS in WT A375 cells in comparison to ERAP1 KO and inhibitor-treated cells (Fig. 5). H2DCFDA is a non-fluorescent compound that is transformed in the fluorescent DCF in the presence of ROS. WT A375 cells showed a significant DCF signal indicating substantial ROS activity, a common feature of cancer cells (61). Surprisingly, blocking ERAP1 function either by inhibitor or by genetic silencing resulted in a substantial decrease in DCF signal, indicating a reduction of ROS activity when ERAP1 activity is blocked (Fig. 5, *B* and *C*). To further confirm this finding, the experiment was repeated with three different clones from the ERAP1 KO cells (1B12 also used in Fig. 5, 1C4 and 2F1). The results indicated indeed that a diminished ROS activity is present in all three clones (Supplemental Fig. S19). These findings suggest that in A375 cancer cells, ERAP1 activity may promote ROS formation.

### ERAP1 Inhibition Sensitizes Cells to External ER Stress

**Fig. 6:**
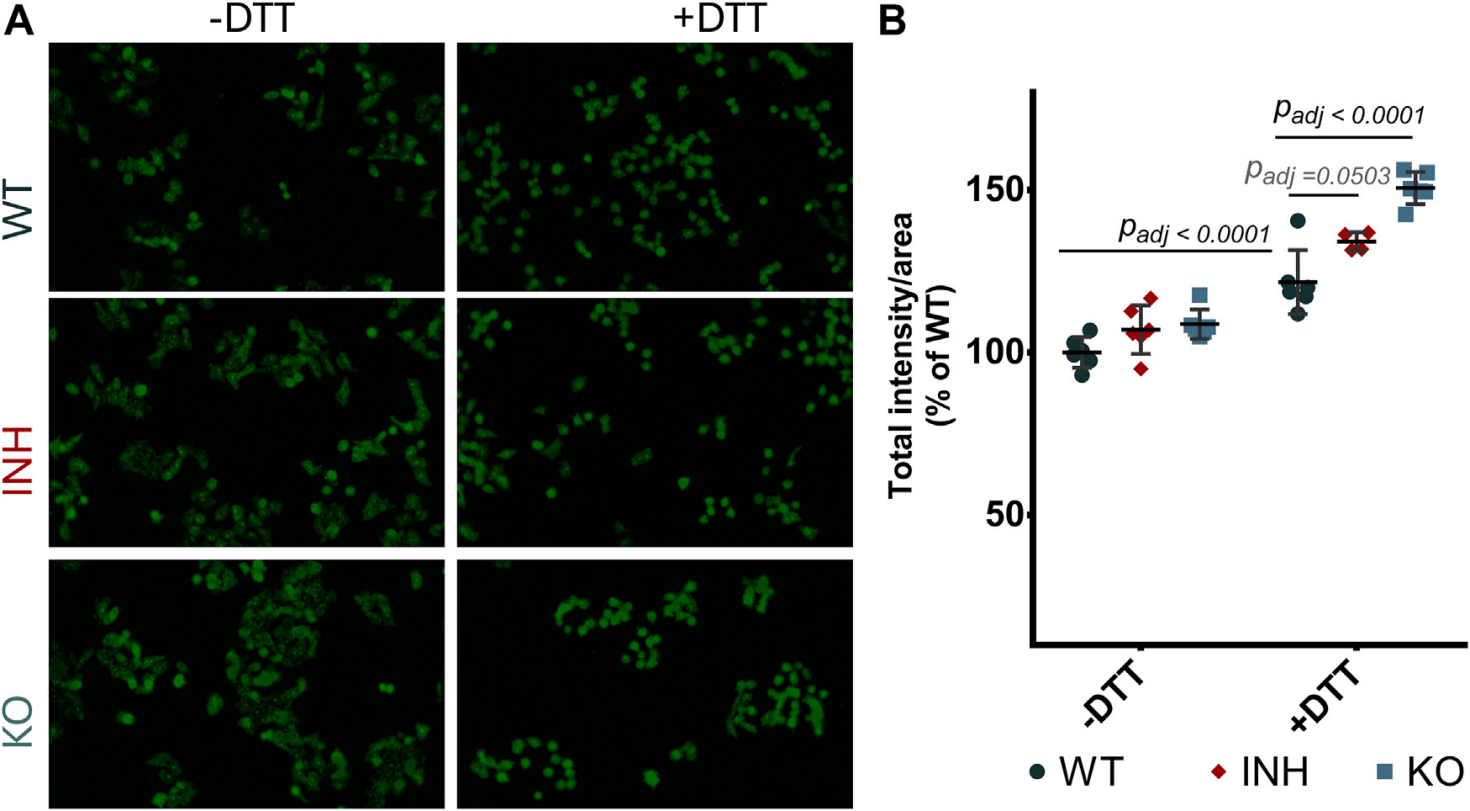
**Thioflavin T assay for ER stress.***Panel A*, representative images of WT, inhibitor-treated (INH), and ERAP1 KO A375 cells incubated with thioflavin T with or without treatment with DTT, as described in the Experimental procedures section. *Panel B*, total fluorescence intensity normalized for cell area for the three biological conditions in the absence or presence of DTT. Statistical significance was evaluated by one-way ANOVA, followed by Dunnett’s multiple comparisons test in GraphPad Prism v.8. ER, endoplasmic reticulum; ERAP, endoplasmic reticulum aminopeptidase.

The observations of the pathway analysis (Supplemental Fig. S14) combined with the effect of ERAP1 inhibition on ROS formation (Fig. 5 and Supplemental Fig. S19), prompted us to investigate the potential effects of ERAP1 activity in ER stress. Indeed, ERAP1 activity in the ER has been associated with ER stress in the case of the MHC-I allele HLA-B∗27. This leads to misfolding and regulates the generation of HLA-B∗27 allele heavy-chain dimers, which induce autoreactive-like immune responses (62, 63, 64). However, due to the unusual folding properties of HLA-B∗27, it is not known if similar effects can occur in different cellular contexts. To investigate this, we used the ThT assay. ThT exhibits increased fluorescence upon binding to protein aggregates and can thus serve as an indicator of ER protein misfolding and ER stress (44). To evaluate the capability of the cells to compensate for external stressors we used DTT, a compound that can interfere with redox potential, disulfide bond formation, and protein folding in the ER. ThT fluorescence was similar between WT, ERAP1-KO, and inhibitor-treated cells (Fig. 6). The addition of DTT, however, led to a significant increase in the signal from WT cells. This increase was much more pronounced in inhibitor-treated and ERAP1-KO cells (Fig. 6 and Supplemental Fig. S20), indicating that the inhibition of ERAP1 activity led to a sensitization of the cells to an external ER stressor.

### ERAP1 can Indirectly Affect Metabolism and Mitochondrial Function

**Fig. 7:**
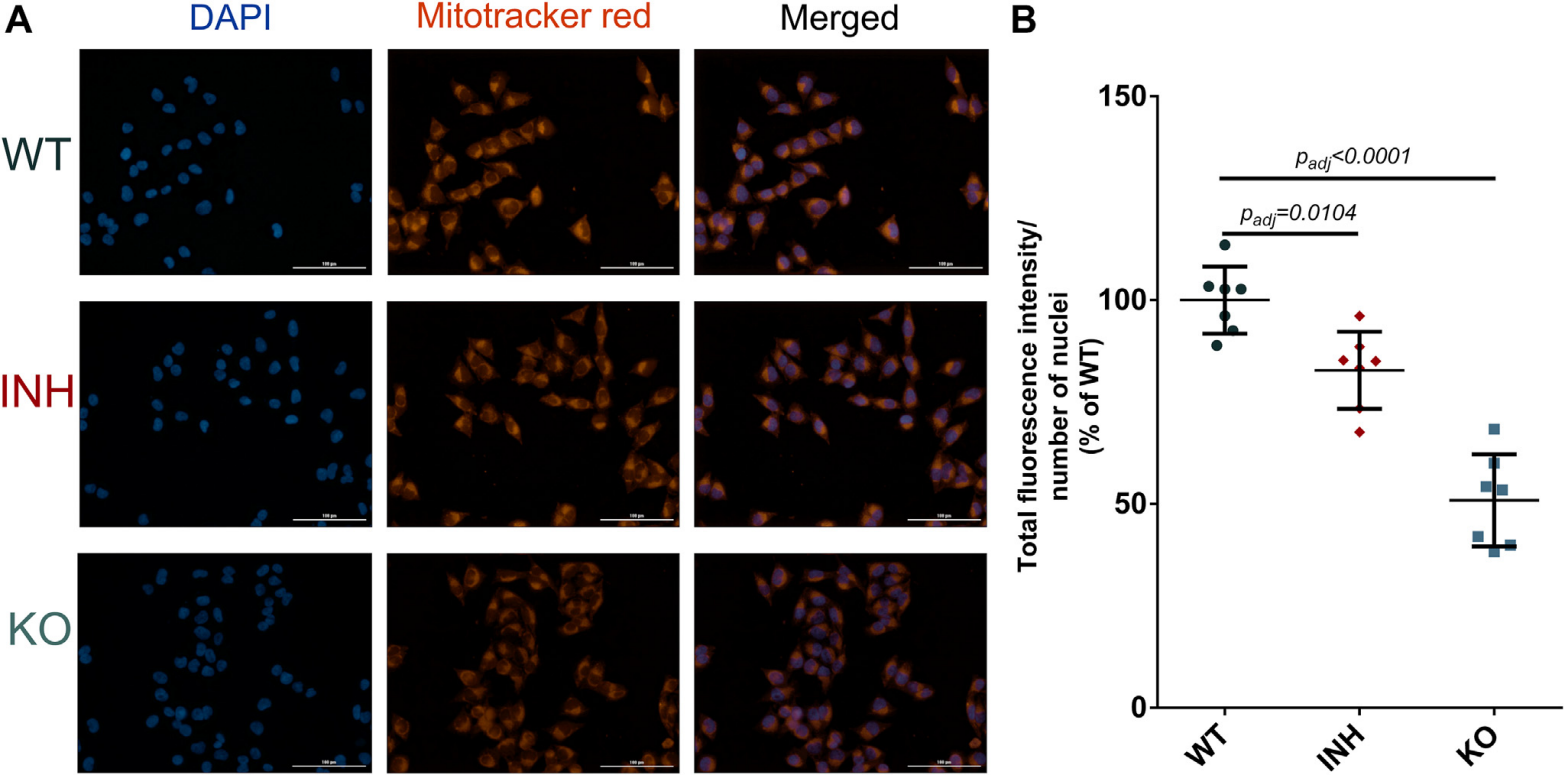
**Mitotracker assay for measurement of mitochondrial membrane potential.***Panel A*, representative fluorescence microscopy images of A375 cells (WT, inhibitor-treated, or ERAP1 KO) visualized after staining with DAPI (*blue*) and Mitotracker (*red*) as well as merged. *Panel B*, total fluorescence intensity of Mitotracker per identified nuclei for the three conditions. The calculated adjusted *p* values comparing KO cells and inhibitor-treated cells to the WT cells are indicated. Statistical significance was evaluated by one-way ANOVA, followed by Dunnett’s multiple comparisons test in GraphPad Prism v.8. ERAP, endoplasmic reticulum aminopeptidase.

Inspired by the pathway analysis which suggested potential changes in oxidative phosphorylation (Supplemental Fig. S14), we identified several relevant proteins in our dataset (Supplemental Fig. S21) and we used the Mitotracker Red CMXRos probe to explore potential changes in mitochondrial membrane potential (65). Mitochondria can communicate and cooperate with the ER to maintain cellular homeostasis (66). We incubated WT, ERAP1 KO, and inhibitor-treated A375 cells with the Mitotracker probe before fixation and DAPI staining and normalized the probe’s fluorescence with the number of nuclei (Fig. 7*A*). Interestingly, both ERAP1 inhibition and ERAP1 KO resulted in a significant decrease of Mitotracker signal (Fig. 7 and Supplemental Fig. S22), suggesting a reduction in mitochondrial membrane potential (Δψ_μ_) upon ERAP1 functional disruption. This finding could indicate that loss of ERAP1 activity in the ER indirectly affects mitochondrial function leading to partial depolarization, which can then affect oxidative phosphorylation.

### Extracellular Flux Analysis

**Fig. 8:**
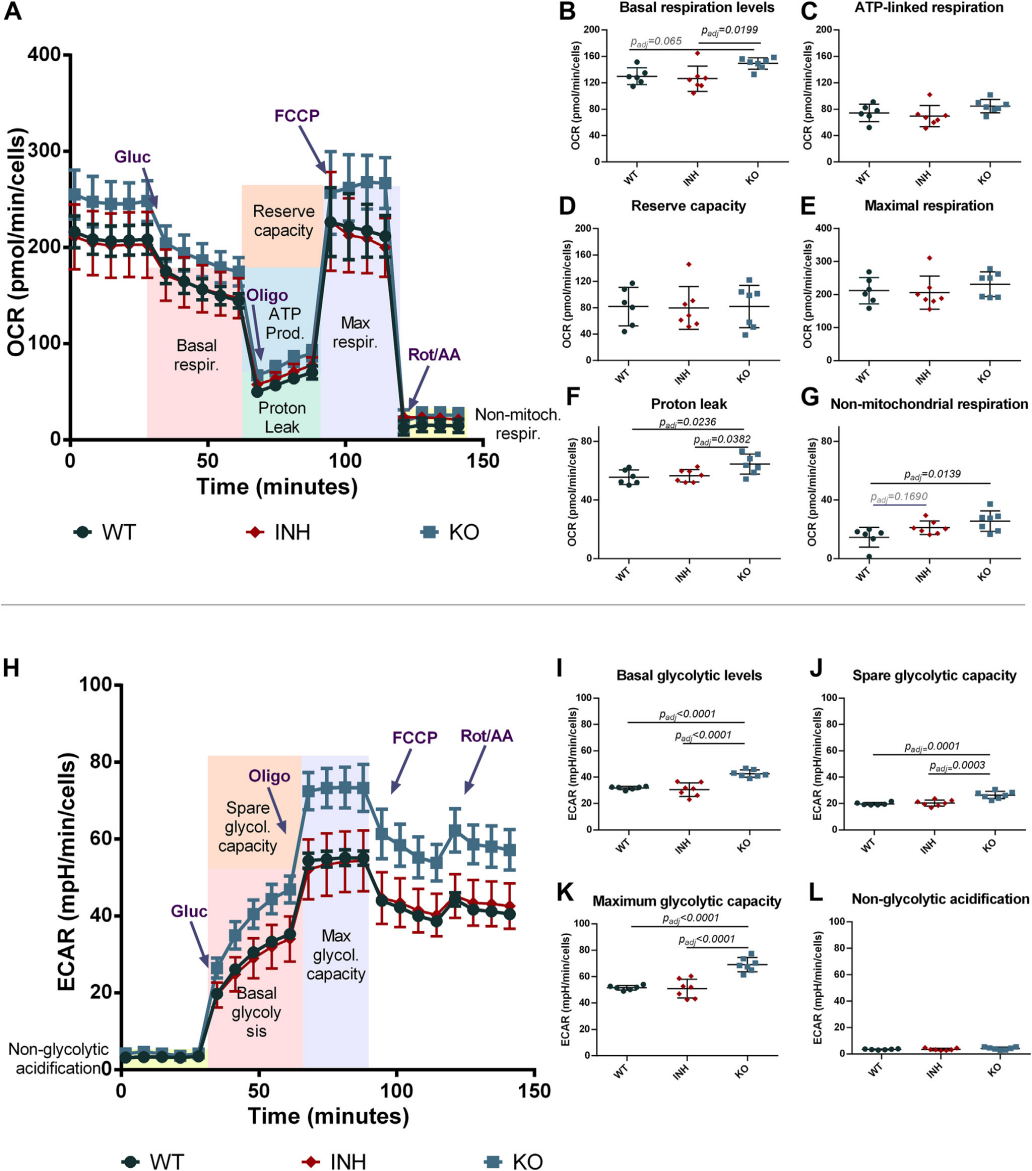
**Seahorse extracellular flux analysis of effects of ERAP1 disruption on cellular metabolism.***Panel A*, Oxygen Consumption Rate (OCR) followed as a function of time during the experiment for WT, inhibitor-treated and ERAP1 KO A375 cells. Arrows at specific time points indicate the addition of glucose, oligomycin, carbonyl cyanide-p-trifluoromethoxyphenylhydrazone (FCCP) and rotenone/antimycin A to probe different metabolic components, as indicated by the colored regions in the figure. *Panels B*–*G*, quantification and comparison of components of mitochondrial metabolism between the three experimental conditions. *Panel H*, extracellular Acidification Rate (ECAR) followed as a function of time as in *panel A. PanelsI*–*L*, quantification and comparison of basal glycolytic levels, spare glycolytic capacity, maximum glycolytic capacity and non-glycolytic acidification as calculated form the ECAR measurements. Statistical significance was evaluated by one-way ANOVA, followed by Dunnett’s multiple comparisons test in GraphPad Prism v.8.

To better understand the potential impact of ERAP1 function on mitochondrial metabolism we used the Seahorse XF analyzer to measure OCR and ECAR in WT, inhibitor-treated, and KO A375 cells. This experiment can evaluate mitochondrial respiration and glycolytic activity under different conditions and metabolic stressors, revealing differences in the mitochondrial metabolic profile when ERAP1 function is disrupted (Fig. 8). When analyzing the OCR profile, while several parameters were not affected (Fig. 8, panels C–E), we observed a statistically significant increase in the basal respiration levels (Fig. 8*B*), proton leak (Fig. 8*F*) and nonmitochondrial respiration (Fig. 8*G*) in the ERAP1 KO cells. A trend toward increased nonmitochondrial respiration was also observed in the inhibitor-treated cells, although much less pronounced. This finding may suggest some limited mitochondrial dysfunction, which is in line with changes in Mitotracker signal described above. Alternatively, this finding may indicate increases in energy demand that cannot be easily satisfied by regular mitochondrial respiration. Accordingly, our analysis revealed some increases in basal glycolytic levels, spare and maximum glycolytic capacity (Fig. 8, panels I–K) also indicating small changes in energy production in the ERAP1 KO cells. Taken together, these changes suggest that loss of ERAP1 may induce some metabolic pressure onto mitochondria that leads to proteomic and metabolic responses.

## Discussion

ERAP1 has been primarily studied for its role in generating antigenic peptides and is considered to have a significant specialization for this role. However other roles have been proposed, albeit less well characterized (25, 26, 27, 28, 29, 30, 31, 32, 33). Indeed, metallo-aminopeptidases, such as ERAP1, often have multiple biological functions depending on the cellular context, tissue, cell type, cellular compartment, and pathophysiological context (67).

Here, we combined immunopeptidomics with proteomic, biochemical, and metabolic analyses to better understand the effect of ERAP1 activity on cancer cells. We find that ERAP1 is a key regulator of the cellular immunopeptidome, and the mechanism of ERAP1 modulation is reflected on the nature of the immunopeptidome, consistent with previous reports (19). We furthermore show that regulation of the immunopeptidome results in modest increases in immunogenicity, which could be a consequence of alterations in the levels of antigenic peptides and potential neoantigens originating from either annotated or unannotated protein ORFs.

To our surprise, we also find that ERAP1 inhibition induces significant shifts in the cellular proteome. Since the immunopeptidome is generated from the sampling of cellular proteins, proteome shifts may contribute to immunopeptidome shifts. However, the extent to which the immunopeptidome represents the cellular proteome is a matter of significant debate. Several studies have argued on whether the immunopeptidome is a real-time representation of all proteins in the cell or a directed sampling of the dynamic changes in protein synthesis (8, 68). Here, we find that ERAP1 genetic silencing or pharmacological inhibition has repercussions on the cellular proteome and that these proteomic changes contribute to the immunopeptidome changes only to a limited extent. Less than 1 out of 10 peptides in the immunopeptidome belong to proteins that are altered by ERAP1 inhibition. Additionally, less than 1 out of 25 proteins that are affected by ERAP1 inhibition carry peptide sequences that are altered in the immunopeptidome. The discrepancy between the proteome and immunopeptidome shifts suggests that some proteins may be preferentially represented in the immunopeptidome. This is consistent with the idea that antigen presentation is not an unbiased constant sampling of the cell proteome but rather a more directed process, reporting select changes in protein content upon infection (8). Spatial compartmentalization of antigen presentation may also contribute to this specialization (69). Moreover, the limited contribution of the proteome to the immunopeptidome indicates that the effects of ERAP1 on antigen presentation are dominated by its direct role in preparing antigenic peptides over indirect effects on the cellular proteome. In this context, these results strengthen the notion that ERAP1 is a specialized enzyme in editing the immunopeptidome.

Despite the small representation of proteomic changes on the cell surface, the scale of some of these changes was surprisingly large, indicating a secondary, but potentially important, role of ERAP1 on cellular homeostasis. A comparable effect was also reproduced to a reasonable extent in another cancer cell line carrying a different combination of ERAP1 haplotypes. This suggests that proteomic changes are not an isolated property of A375 cells but could be a fundamental consequence of the enzyme’s function in maintaining peptide homeostasis in the ER, which could affect protein folding and indirectly contribute to ER stress. Indeed, in the case of the MHCI-allele–dependent inflammatory disease, Ankylosing Spondylitis, the pathogenetic role of ERAP1 has also been proposed to be linked to ER misfolding of HLA-B∗27, the formation of heavy chain dimers and general ER stress (63, 64).

It should be noted, however, that the cellular systems explored here (A375 and THP-1 cells) are both cancer cell lines which may not represent normal cell metabolism. Although our observations may not extend to noncancer cell lines, ERAP1 is a pharmacological target in cancer therapy and any effects of its inhibition on cancer cell function may have to be validated and evaluated separately to its effect on the immune responses. For this reason, we built on proteomic findings with additional biochemical and metabolic analyses. We find that in the A375 melanoma cell line, ERAP1 functional disruption appears to affect the capability of the ER to respond to redox challenges. These effects are accompanied by changes in the levels of ROS and metabolic changes focused on mitochondrial membrane polarization and extending to glycolytic potential and nonmitochondrial respiration. Specifically, we observed a mild increase in proton leak in the ERAP1 KO cells, in addition to a decrease in mitochondrial membrane potential (Δψ_μ_) and ROS in both treatment conditions. Mild proton leak has been suggested as a protective mechanism against oxidative damage by reducing ROS formation (70, 71, 72, 73) due to a lower mitochondrial membrane potential (Δψ_μ_) (73). This mechanism may prevent excessive electron supply to the respiratory chain, thereby lowering the chance of electron leakage and subsequent superoxide production. Moreover, a trend toward increased nonmitochondrial respiration was observed upon ERAP1 inhibition. Although nonmitochondrial respiration has not been adequately defined yet, several researchers have linked it to processes including nitric oxide–mediated metabolic effects (74), cell surface oxygen consumption (75), and peroxisomal respiration (76). The latter might be particularly relevant here since emerging evidence suggests that peroxisomes, besides generating ROS, can also diminish them, depending on the cellular context (76). Besides peroxisomal respiration, diminishment of ROS can also occur indirectly as a result of increased glycolysis in cancer cells (71), also known as the Warburg effect (77), due to diversion of substrates from the electron transport chain, which we also observed in the ERAP1 KO cells.

Although the exact mechanism is not clear, the metabolic changes described above may be related to the role of ERAP1 in peptide homeostasis in the ER. Lack of ERAP1 peptide trimming may lead to an increase in peptide concentration inside the ER, an effect that could interfere with proper protein folding. In the presence of an ER folding stressor, DTT, ERAP1 inhibition significantly affected protein folding in the ER. This may be revealing an underlying folding problem, which was not observed in the absence of DTT, likely due to the buffering capacity of the ER environment or the sensitivity of the used method. Induction of ER stress could then be communicated to the mitochondria, given the established relationship between the two compartments, and induce metabolic changes that would help the cell adapt to the new conditions (66). Overall, these responses may underlie the proteome changes reported here and represent a homeostatic role for ERAP1. Indeed, this aspect of ERAP1 function has been hypothesized as a pathogenetic mechanism in HLA-associated autoimmunity (64, 78).

Overall, our results support the proposed dominant role of ERAP1 as an immunopeptidome editor, while revealing secondary, but potentially important effects in metabolic homeostasis. While the importance of these effects in cancer progression is unclear, it is possible that cancer cells may have to strike a balance between promoting immune evasion and maintaining viable cellular homeostasis. Still, the breadth of most described effects is modest, which highlights, rather than negates, the specialized role of ERAP1 in regulating adaptive immune responses. Since ERAP1 is a target molecule for cancer immunotherapy, it may be worth exploring whether its secondary effects on the proteome and metabolism of cancer may be exploited pharmacologically to synergize with the effects on adaptive immunity to enhance antitumor therapies.

## Data Availability

All the data described are available in the article and associated supporting information. Numerical values used for the generation of graphs are available upon request to the corresponding author (Efstratios Stratikos; E-mail: estratikos@chem.uoa.gr or stratos@rrp.demokritos.gr). The MS proteomics raw data have been deposited to the ProteomeXchange Consortium via the PRIDE (79) partner repository with the dataset identifiers PXD054491 and PXD054494 (immunopeptidomics), PXD060572 (A375 proteomics), and PXD060575 (THP-1 proteomics) (http://www.ebi.ac.uk/pride/archive/).

## Supporting Information

This article contains supporting information (49, 57, 58, 80).

## Conflict of Interest

The authors declare no competing interests.

## References

[1] Jensen P.E. (2007). Recent advances in antigen processing and presentation. Nat. Immunol..

[2] York I.A., Rock K.L. (1996). Antigen processing and presentation by the class I major histocompatibility complex. Annu. Rev. Immunol..

[3] Rock K.L., Goldberg A.L. (1999). Degradation of cell proteins and the generation of MHC class I-presented peptides. Annu. Rev. Immunol..

[4] Pishesha N., Harmand T.J., Ploegh H.L. (2022). A guide to antigen processing and presentation. Nat. Rev. Immunol..

[5] Shastri N., Schwab S., Serwold T. (2002). Producing nature’s gene-chips: the generation of peptides for display by MHC class I molecules. Annu. Rev. Immunol..

[6] Lanier L.L. (2008). Up on the tightrope: natural killer cell activation and inhibition. Nat. Immunol..

[7] Admon A., Bassani-Sternberg M. (2011). The human immunopeptidome project, a suggestion for yet another postgenome next big thing. Mol. Cell. Proteomics.

[8] Yewdell J.W., Hollý J. (2020). DRiPs get molecular. Curr. Opin. Immunol..

[9] Vyas J.M., Van Der Veen A.G., Ploegh H.L. (2008). The known unknowns of antigen processing and presentation. Nat. Rev. Immunol..

[10] Kisselev A.F., Akopian T.N., Woo K.M., Goldberg A.L. (1999). The sizes of peptides generated from protein by mammalian 26 and 20 S proteasomes. J. Biol. Chem..

[11] Saric T., Chang S.-C., Hattori A., York I.A., Markant S., Rock K.L. (2002). An IFN-γ–Induced aminopeptidase in the ER, ERAP1, trims precursors to MHC class I–presented peptides. Nat. Immunol..

[12] Tanioka T., Hattori A., Masuda S., Nomura Y., Nakayama H., Mizutani S. (2003). Human leukocyte-derived arginine aminopeptidase. J. Biol. Chem..

[13] Tanioka T., Hattori A., Mizutani S., Tsujimoto M. (2005). Regulation of the human leukocyte-derived arginine aminopeptidase endoplasmic reticulum-aminopeptidase 2 gene by interferon-γ. FEBS J..

[14] Stratikos E., Stern L.J. (2013). Antigenic peptide trimming by ER aminopeptidases—insights from structural studies. Mol. Immunol..

[15] Blees A., Januliene D., Hofmann T., Koller N., Schmidt C., Trowitzsch S. (2017). Structure of the human MHC-I peptide-loading complex. Nature.

[16] Shapiro I.E., Bassani-Sternberg M. (2023). The impact of immunopeptidomics: from basic Research to clinical implementation. Semin. Immunol..

[17] Nagarajan N.A., de Verteuil D.A., Sriranganadane D., Yahyaoui W., Thibault P., Perreault C. (2016). ERAAP shapes the peptidome associated with classical and nonclassical MHC class I molecules. J. Immunol..

[18] Koumantou D., Barnea E., Martin-Esteban A., Maben Z., Papakyriakou A., Mpakali A. (2019). Editing the immunopeptidome of melanoma cells using a potent inhibitor of endoplasmic reticulum aminopeptidase 1 (ERAP1). Cancer Immunol. Immunother..

[19] Temponeras I., Samiotaki M., Koumantou D., Nikopaschou M., Kuiper J.J.W., Panayotou G. (2023). Distinct modulation of cellular immunopeptidome by the allosteric regulatory site of ER aminopeptidase 1. Eur. J. Immunol..

[20] Barnea E., Melamed Kadosh D., Haimovich Y., Satumtira N., Dorris M.L., Nguyen M.T. (2017). The human leukocyte antigen (HLA)-B27 peptidome *in Vivo*, in spondyloarthritis-susceptible HLA-B27 transgenic rats and the effect of Erap1 deletion. Mol. Cell. Proteomics.

[21] Georgiadis D., Mpakali A., Koumantou D., Stratikos E. (2019). Inhibitors of ER aminopeptidase 1 and 2: from design to clinical application. Curr. Med. Chem..

[22] Fougiaxis V., He B., Khan T., Vatinel R., Koutroumpa N.M., Afantitis A. (2024). ERAP inhibitors in autoimmunity and immuno-oncology: medicinal chemistry insights. J. Med. Chem..

[23] Liddle J., Hutchinson J.P., Kitchen S., Rowland P., Neu M., Cecconie T. (2020). Targeting the regulatory site of ER aminopeptidase 1 leads to the Discovery of a natural product modulator of antigen presentation. J. Med. Chem..

[24] Maben Z., Arya R., Rane D., An W.F., Metkar S., Hickey M. (2020). Discovery of selective inhibitors of endoplasmic reticulum aminopeptidase 1. J. Med. Chem..

[25] Hattori A., Kitatani K., Matsumoto H., Miyazawa S., Rogi T., Tsuruoka N. (2000). Characterization of recombinant human adipocyte-derived leucine aminopeptidase expressed in Chinese hamster ovary cells. J. Biochem. (Tokyo).

[26] Hisatsune C., Ebisui E., Usui M., Ogawa N., Suzuki A., Mataga N. (2015). ERp44 exerts redox-dependent control of blood pressure at the ER. Mol. Cell.

[27] Watanabe Y., Shibata K., Kikkawa F., Kajiyama H., Ino K., Hattori A. (2003). Adipocyte-Derived leucine aminopeptidase suppresses angiogenesis in human endometrial carcinoma via renin-angiotensin system. Clin. Cancer Res..

[28] Cui X., Hawari F., Alsaaty S., Lawrence M., Combs C.A., Geng W. (2002). Identification of ARTS-1 as a novel TNFR1-binding protein that promotes TNFR1 ectodomain shedding. J. Clin. Invest..

[29] Cui X., Rouhani F.N., Hawari F., Levine S.J. (2003). An aminopeptidase, ARTS-1, is required for interleukin-6 receptor shedding. J. Biol. Chem..

[30] Bufalieri F., Infante P., Bernardi F., Caimano M., Romania P., Moretti M. (2019). ERAP1 promotes hedgehog-dependent tumorigenesis by controlling USP47-mediated degradation of βTrCP. Nat. Commun..

[31] Aldhamen Y.A., Seregin S.S., Rastall D.P.W., Aylsworth C.F., Pepelyayeva Y., Busuito C.J. (2013). Endoplasmic reticulum aminopeptidase-1 functions regulate key aspects of the innate immune response. PLoS One.

[32] Aldhamen Y.A., Pepelyayeva Y., Rastall D.P.W., Seregin S.S., Zervoudi E., Koumantou D. (2015). Autoimmune disease-associated variants of extracellular endoplasmic reticulum aminopeptidase 1 induce altered innate immune responses by human immune cells. J. Innate Immun..

[33] Blake M.K., O’Connell P., Pepelyayeva Y., Godbehere S., Aldhamen Y.A., Amalfitano A. (2022). ERAP1 is a critical regulator of inflammasome-mediated proinflammatory and ER stress responses. BMC Immunol..

[34] Caron E., Vincent K., Fortier M., Laverdure J., Bramoullé A., Hardy M. (2011). The MHC I immunopeptidome conveys to the cell surface an integrative view of cellular regulation. Mol. Syst. Biol..

[35] Cox J., Mann M. (2007). Is proteomics the new genomics?. Cell.

[36] Venema W.J., Hiddingh S., Van Loosdregt J., Bowes J., Balliu B., De Boer J.H. (2024). A cis-regulatory element regulates ERAP2 expression through autoimmune disease risk SNPs. Cell Genomics.

[37] Temponeras I., Stamatakis G., Samiotaki M., Georgiadis D., Pratsinis H., Panayotou G. (2022). ERAP2 inhibition induces cell-surface presentation by MOLT-4 leukemia cancer cells of many novel and potentially antigenic peptides. Int. J. Mol. Sci..

[38] Hughes C.S., Moggridge S., Müller T., Sorensen P.H., Morin G.B., Krijgsveld J. (2019). Single-pot, solid-phase-enhanced sample preparation for proteomics experiments. Nat. Protoc..

[39] Ouspenskaia T., Law T., Clauser K.R., Klaeger S., Sarkizova S., Aguet F. (2022). Unannotated proteins expand the MHC-I-restricted immunopeptidome in cancer. Nat. Biotechnol..

[40] Demichev V., Messner C.B., Vernardis S.I., Lilley K.S., Ralser M. (2020). DIA-NN: Neural networks and interference correction enable Deep proteome coverage in high throughput. Nat. Methods.

[41] Tyanova S., Temu T., Sinitcyn P., Carlson A., Hein M.Y., Geiger T. (2016). The Perseus computational platform for comprehensive analysis of (Prote)Omics data. Nat. Methods.

[42] Pino L.K., Searle B.C., Bollinger J.G., Nunn B., MacLean B., MacCoss M.J. (2020). The skyline ecosystem: informatics for quantitative mass spectrometry proteomics. Mass Spectrom. Rev..

[43] Schneider C., Rasband W., Eliceiri K. (2012). NIH image to ImageJ: 25 years of image analysis. Nat. Methods.

[44] Beriault D.R., Werstuck G.H. (2013). Detection and quantification of endoplasmic reticulum stress in living cells using the fluorescent compound, Thioflavin T. Biochim. Biophys. Acta.

[45] Schindelin J., Arganda-Carreras I., Frise E. (2012). Fiji: an open-source platform for biological-image analysis. Nat. Methods.

[46] Kaabinejadian S., Barra C., Alvarez B., Yari H., Hildebrand W.H., Nielsen M. (2022). Accurate MHC motif deconvolution of immunopeptidomics data reveals a significant contribution of DRB3, 4 and 5 to the total DR immunopeptidome. Front. Immunol..

[47] Reynisson B., Alvarez B., Paul S., Peters B., Nielsen M. (2020). NetMHCpan-4.1 and NetMHCIIpan-4.0: improved predictions of MHC antigen presentation by concurrent motif deconvolution and integration of MS MHC eluted ligand data. Nucleic Acids Res..

[48] Giastas P., Mpakali A., Papakyriakou A., Lelis A., Kokkala P., Neu M. (2019). Mechanism for antigenic peptide selection by endoplasmic reticulum aminopeptidase 1. Proc. Natl. Acad. Sci. U. S. A..

[49] Li G., Iyer B., Prasath V.B.S., Ni Y., Salomonis N. (2021). DeepImmuno: Deep learning-empowered prediction and generation of immunogenic peptides for T-cell immunity. Brief. Bioinform.

[50] Vita R, Blazeska N, Marrama D, Curation Team Members, Duesing S, Bennett J (2025). The immune epitope database (IEDB): 2024 update. Nucleic Acids Res..

[51] James E., Bailey I., Sugiyarto G., Elliott T. (2013). Induction of protective antitumor immunity through attenuation of ERAAP function. J. Immunol..

[52] Stratikos E. (2014). Regulating adaptive immune responses using small molecule modulators of aminopeptidases that process antigenic peptides. Curr. Opin. Chem. Biol..

[53] Cifaldi L., Romania P., Falco M., Lorenzi S., Meazza R., Petrini S. (2015). ERAP1 regulates natural killer cell function by controlling the engagement of inhibitory receptors. Cancer Res..

[54] D’Amico S., D’Alicandro V., Compagnone M., Tempora P., Guida G., Romania P. (2021). ERAP1 controls the interaction of the inhibitory receptor KIR3DL1 with HLA-B51:01 by affecting natural killer cell function. Front. Immunol..

[55] Cifaldi L., Lo Monaco E., Forloni M., Giorda E., Lorenzi S., Petrini S. (2011). Natural killer cells efficiently reject lymphoma silenced for the endoplasmic reticulum aminopeptidase associated with antigen processing. Cancer Res..

[56] Dragovic S.M., Hill T., Christianson G.J., Kim S., Elliott T., Scott D. (2011). Proteasomes, TAP, and endoplasmic reticulum-associated aminopeptidase associated with antigen processing control CD4+ Th cell responses by regulating indirect presentation of MHC class II-restricted cytoplasmic antigens. J. Immunol. Baltim. Md..

[57] Ge S.X., Jung D., Yao R. (2020). ShinyGO: A graphical gene-set enrichment tool for animals and plants. Bioinformatics.

[58] Kanehisa M., Furumichi M., Sato Y., Kawashima M., Ishiguro-Watanabe M. (2023). KEGG for taxonomy-based analysis of pathways and genomes. Nucleic Acids Res..

[59] Velarde G., Ford R.C., Rosenberg M.F., Powis S.J. (2001). Three-dimensional structure of transporter associated with antigen processing (TAP) obtained by single particle image analysis. J. Biol. Chem..

[60] Reeves E., Edwards C.J., Elliott T., James E. (2013). Naturally occurring ERAP1 haplotypes encode functionally distinct alleles with fine substrate specificity. J. Immunol..

[61] Zhao Y., Ye X., Xiong Z., Ihsan A., Ares I., Martínez M. (2023). Cancer metabolism: the role of ROS in DNA damage and induction of apoptosis in cancer cells. Metabolites.

[62] Chen L., Ridley A., Hammitzsch A., Al-Mossawi M.H., Bunting H., Georgiadis D. (2016). Silencing or inhibition of endoplasmic reticulum aminopeptidase 1 (ERAP1) suppresses free heavy chain expression and Th17 responses in ankylosing spondylitis. Ann. Rheum. Dis..

[63] Babaie F., Mohammadi H., Salimi S., Ghanavatinegad A., Abbasifard M., Yousefi M. (2023). Inhibition of ERAP1 represses HLA-B27 free heavy chains expression on polarized macrophages and interrupts NK cells activation and function from ankylosing spondylitis. Clin. Immunol. Orlando Fla..

[64] Tran T.M., Gill T., Bennett J., Hong S., Holt V., Lindstedt A.J. (2023). Paradoxical effects of endoplasmic reticulum aminopeptidase 1 deficiency on HLA-B27 and its role as an epistatic modifier in experimental spondyloarthritis. Arthritis Rheumatol. Hoboken NJ.

[65] Kholmukhamedov A., Schwartz J.M., Lemasters J.J. (2013). MitoTracker probes and mitochondrial membrane potential. Shock Augusta Ga..

[66] Malhotra J.D., Kaufman R.J. (2011). ER stress and its functional link to mitochondria: role in cell survival and death. Cold Spring Harb. Perspect. Biol..

[67] Chen A.Y., Adamek R.N., Dick B.L., Credille C.V., Morrison C.N., Cohen S.M. (2019). Targeting metalloenzymes for therapeutic intervention. Chem. Rev..

[68] Rock K.L., Farfán-Arribas D.J., Colbert J.D., Goldberg A.L. (2014). Re-examining class-I presentation and the DRiP hypothesis. Trends Immunol..

[69] Koller N., Höllthaler P., Barends M., Döring M., Spahn C., Durán V. (2022). Nanoscale organization of the MHC I peptide-loading complex in human dendritic cells. Cell. Mol. Life Sci. CMLS.

[70] Korshunov S., Skulachev V., Starkov A. (1997). High protonic potential actuates a mechanism of production of reactive oxygen species in mitochondria. FEBBS Lett..

[71] Baffy G., Derdak Z., Robson S.C. (2011). Mitochondrial recoupling: a novel therapeutic strategy for cancer?. Br. J. Cancer.

[72] Divakaruni A.S., Brand M.D. (2011). The regulation and physiology of mitochondrial proton leak. Physiology.

[73] Cadenas S. (2018). Mitochondrial uncoupling, ROS generation and cardioprotection. Biochim. Biophys. Acta BBA - Bioenerg..

[74] Vagher B., Amiel E. (2024). Detection of nitric oxide-mediated metabolic effects using real-time extracellular flux analysis. PLoS One.

[75] Herst P.M., Berridge M.V. (2007). Cell surface oxygen consumption: a major contributor to cellular oxygen consumption in glycolytic cancer cell lines. Biochim. Biophys. Acta BBA - Bioenerg..

[76] He A., Dean J.M., Lodhi I.J. (2021). Peroxisomes as cellular adaptors to metabolic and environmental stress. Trends Cell Biol..

[77] Warburg O. (1956). On the origin of cancer cells. Science.

[78] Kuiper J.J., Prinz J.C., Stratikos E., Kuśnierczyk P., Arakawa A., Springer S. (2023). EULAR study group on “MHC-I-opathy”: identifying disease-overarching mechanisms across disciplines and borders. Ann. Rheum. Dis..

[79] Perez-Riverol Y., Csordas A., Bai J., Bernal-Llinares M., Hewapathirana S., Kundu D.J. (2019). The PRIDE database and related tools and Resources in 2019: improving support for quantification data. Nucleic Acids Res..

[80] Korotkevich G., Sukhov V., Budin N., Shpak B., Artyomov M.N., Sergushichev A. (2016). Fast gene set enrichment analysis. bioRxiv.

