# Supplementary material for: ERAP1 Activity Modulates the Immunopeptidome but Also Affects the Proteome, Metabolism, and Stress Responses in Cancer Cells
Source: Mol Cell Proteomics. 2025 Apr 4;24(5):100964. doi: 10.1016/j.mcpro.2025.100964 (PMC12136889; doi:10.1016/j.mcpro.2025.100964)
Supplement: Supplemental Information [file mmc1.pdf]

Supplemental information

ERAP1 activity modulates the immunopeptidome but also affects the proteome, metabolism and stress responses in cancer cells

Martha Nikopaschou[1,2], Martina Samiotaki[3], Elli-Anna Stylianaki[4], Kamila Król[5], Paula Gragera[5], Aroosha Raja[6], Vassilis Aidinis[4], Angeliki Chroni[1], Doriana Fruci[4], George Panayotou[3] and Efstratios Stratikos[1,2.*]

[1] National Centre for Scientific Research Demokritos, Agia Paraskevi, Greece
[2] Department of Chemistry, National and Kapodistrian University of Athens, 15784 Zografou, Greece
[3] Biomedical Sciences Research Center "Alexander Fleming", Institute for Bioinnovation, 16672 Vari, Greece
[4] Biomedical Sciences Research Center "Alexander Fleming", Institute for Fundamental Biomedical Research, Vari, Greece
[5] Division of Pediatric Hematology and Oncology, Bambino Gesù Children's Hospital, IRCCS, Rome, Italy
[6] Center for Translational Immunology, University Medical Center Utrecht, Utrecht University, Utrecht, The Netherlands

*Corresponding author: Efstratios Stratikos, estratikos@chem.uoa.gr

SUPPLEMENTAL FIGURES

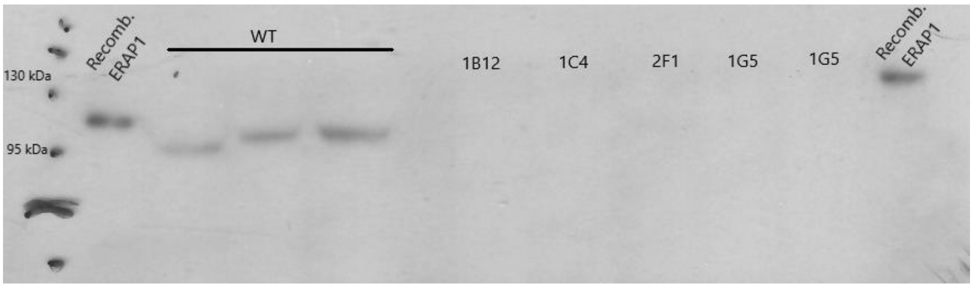

Figure S1. Western blot analysis showing the presence of ERAP1 in the wild-type cells and its absence in the obtained clones.

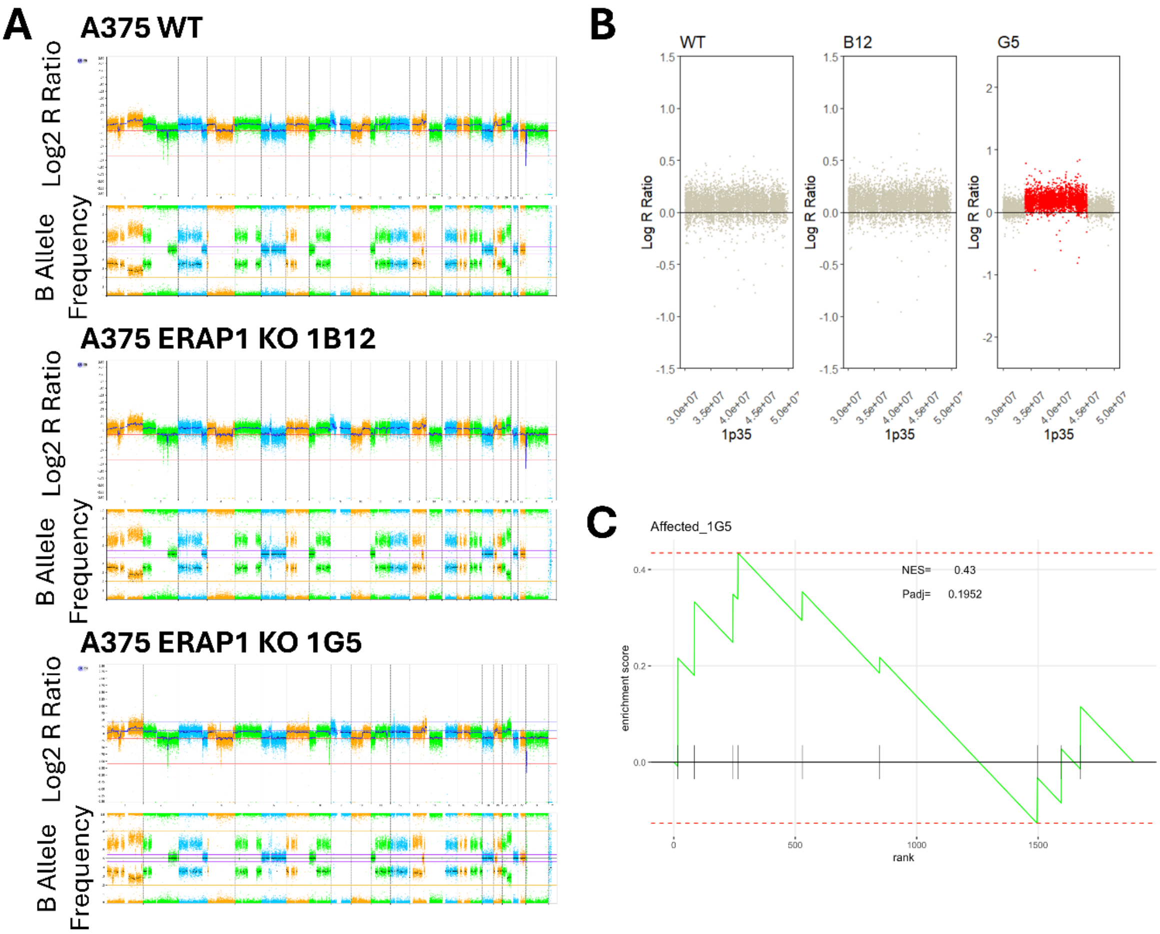

Figure S2. Genome-wide SNP array analysis results. Panel A, *Whole genome copy number profiling and analysis of regions of homozygosity using SNP-arrays of the A375 cells used in this study. SNP-array based copy number profiling and analysis of regions of homozygosity using the Infinium Human CytoSNP-850K v1.2 BeadChip (Illumina, San Diego, CA, USA) showed multiple chromosomal abnormalities, as expected for cancer cells, consistent between the 3 tested clones. The only exception was the area of 1p35 for clone 1G5, in which ~20% of cells showed a partial duplication.* ***Panel B,*** *zoom in 1p35 area, partially affected in 1G5 cells. C. Enrichment analysis was performed with the fgsea package in R.Studio v. 4.3.3 to check whether the differentially expressed proteins in the proteomics experiment were enriched with proteins from the affected area in the 1G5 clone. The results of the analysis indicated that there is no enrichment for proteins in the affected area (Padj = 0.1952).*

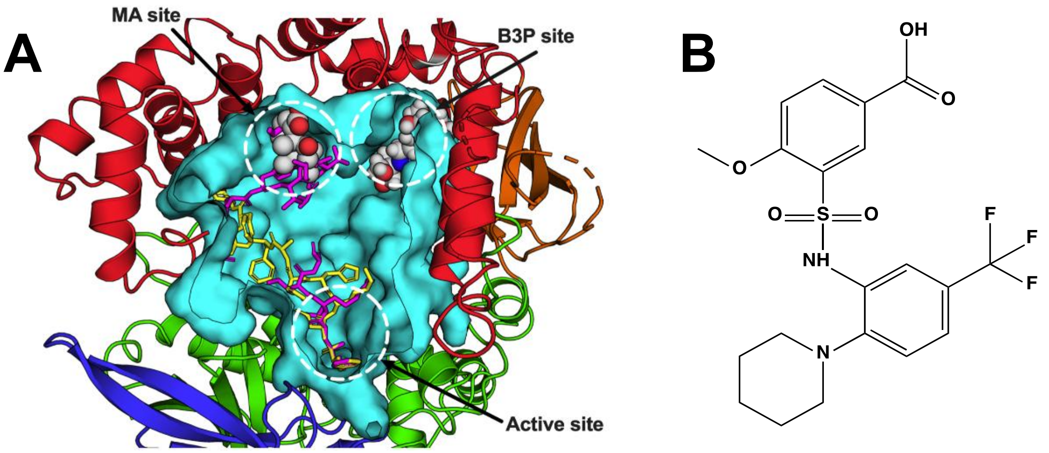

Figure S3. Panel A, *Schematic representation of the internal cavity of ERAP1 (in cyan cutaway view) indicating the active site of the enzyme as well as two allosteric sites found to accommodate small MW compounds in a high-resolution crystal structure (MA = malate, B3P = bis-tris-propane). Two peptide substrates crystallized with ERAP1 are shown in stick representation (10 mer peptide in yellow and 15 mer in magenta).* ***Panel B,*** *the chemical structure of compound 3 used in this study.*

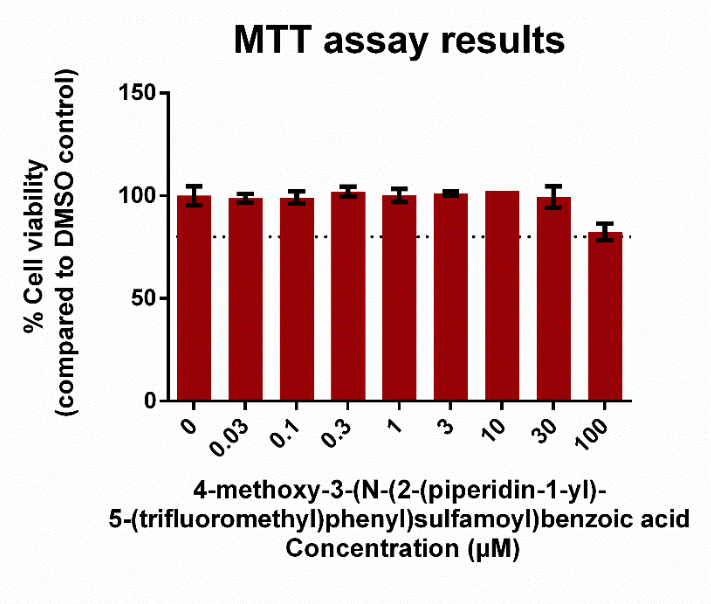

Figure S4. MTT assay results. *The viability of A375 cells was evaluated by the MTT assay after 48hrs exposure to different concentrations of the ERAP1 inhibitor. The dotted line at 80% indicates the threshold for adequate viability.*

A375 cells: exon10 ERAP2 sequencing

FV:
ARRMMRAGKSCYCTTTTTTGTTCTTGTTTTGTARAGKTGTTTAGAAAGTGATTTTACATCTGGTGGAKTWTGT
CATTCGGATCCCRAGATGACAAGTAACATGGKGAGGATAAAGAGAGTCACAGAGTAKAAGAGATCTGTGGA
ATAGCCTGACCTAGAGTGAGKATGACATACAGAGTAGCCCACCTGTCCCTTATWTA

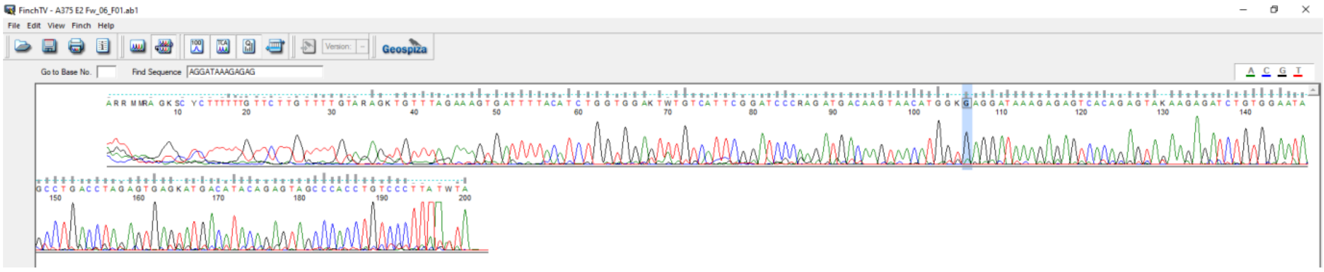

RV:
CTCCGGCTAYCTWAGGTCRGMTATTCCACAGATCTCTTCTACTCTGTGACTCTCTTTATCCTCACCATGTWACT
TGTCATCTTGGGATCCGAATGACAAACTCCACCAGATGTAAAATCACTTTCTAAACAACTCTACAAAACAAGA
ACAAAAGAGCACTGCAATGTAGGCATCATTAGTTGGCTGAACGTGCACCA

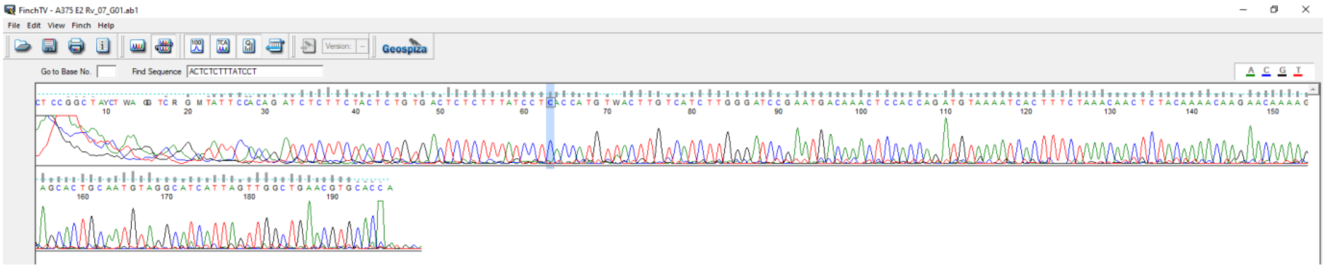

***Figure S5. Sequencing results for ERAP2 exon 10 in A375 cells**, indicating that A375 are homozygous for the unfunctional "GG" allele.*

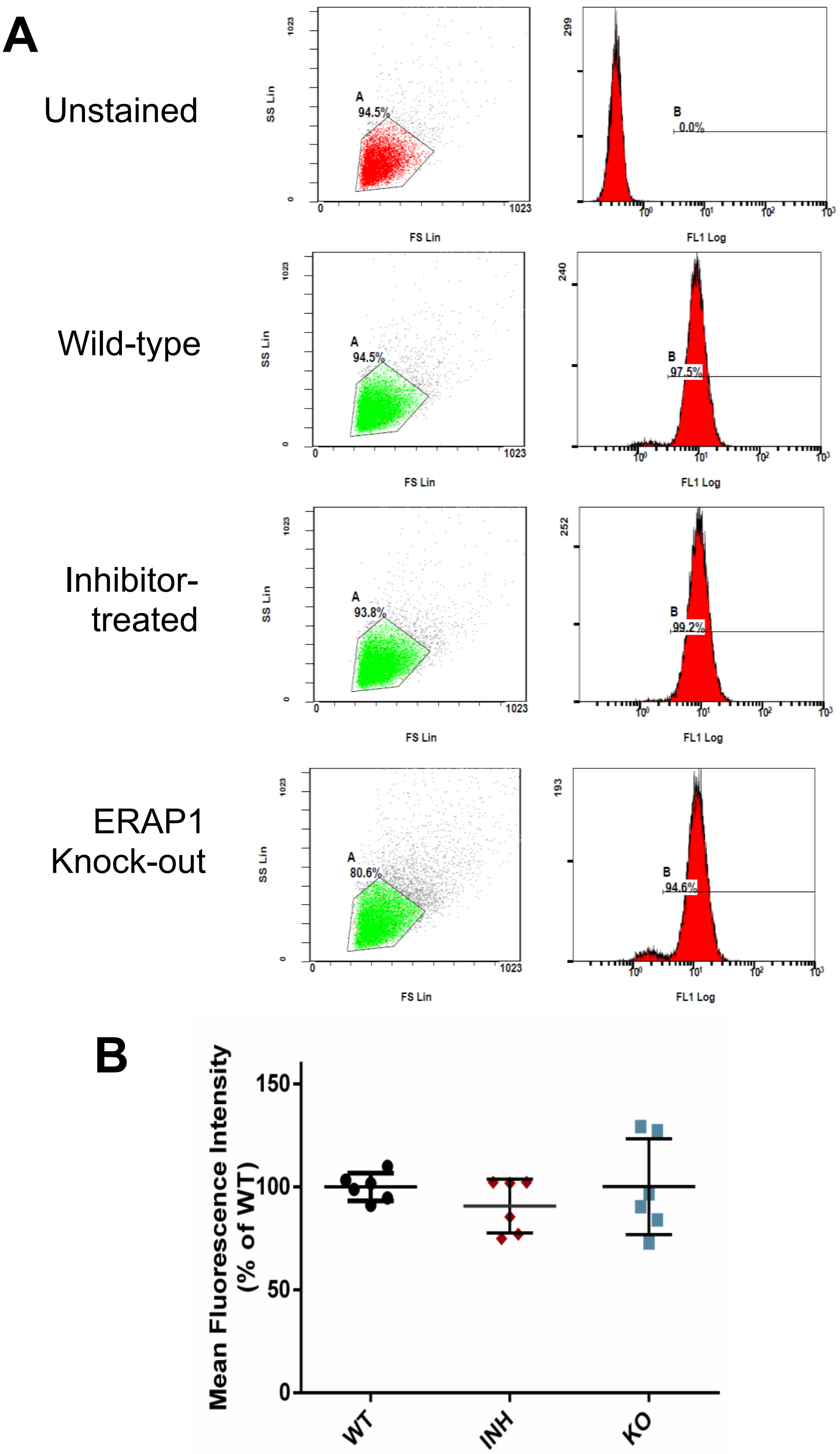

***Figure S6**: **Cell-surface levels of MHC-I in A375 cells detected by FACS. Panel A,** Representative data from each condition indicating gating strategy and signal distribution. **Panel B,** Normalized mean fluorescence intensity (% of Wild-type, DMSO treated cells) from 2 independent experiments with n=3 technical replicates each.*

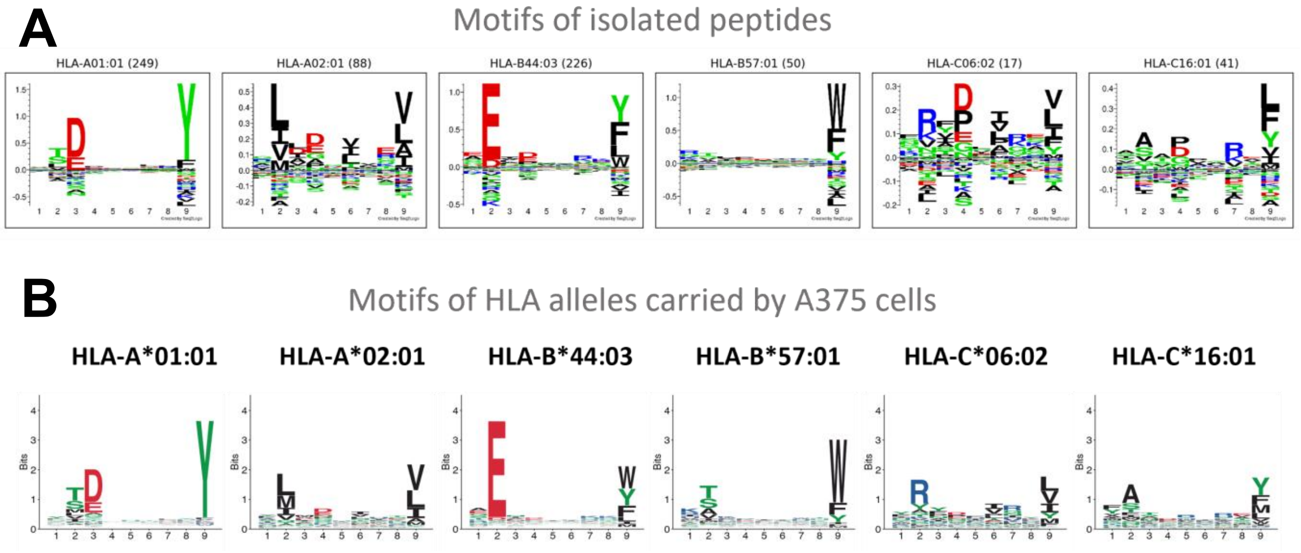

Figure S7: Panel A, *Motifs of isolated peptides between 9 and 14 amino acids for the HLA alleles carried by A375 cells, as predicted by the MHC_Motif_Decon 1.0 server. The motifs contain 95% of the isolated 9-14mers (671 out of 704 peptides).* ***Panel B****, Motifs of HLA alleles carried by A375, downloaded from MHC motif atlas (http://mhcmotifatlas.org/class1).*

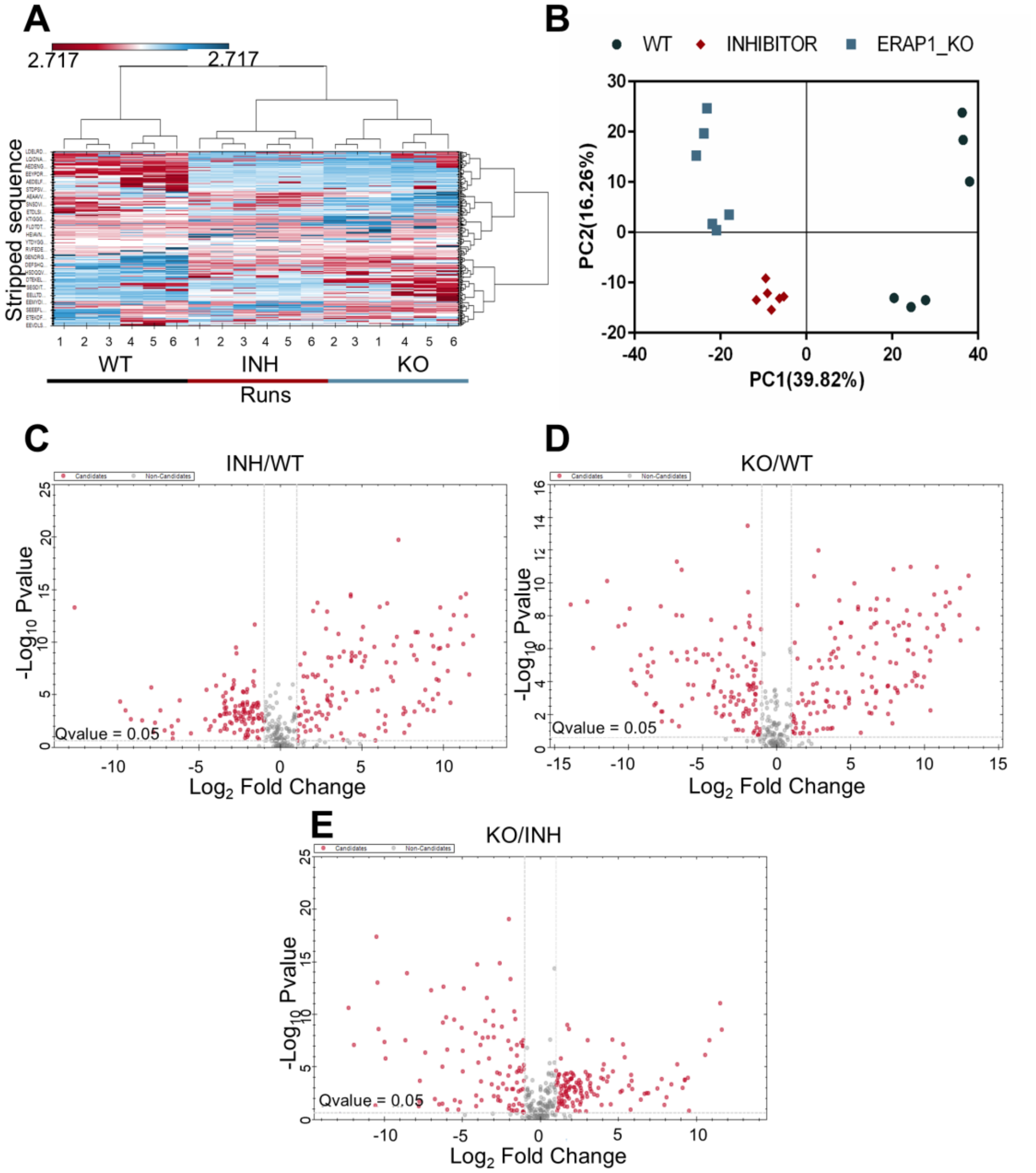

Figure S8: Analysis of the immunopeptidome of wild-type, inhibitor-treated & ERAP1 KO A375 cells (run 2). Panel A, *Heatmap from LC-MS/MS run 2 showcasing peptide distribution across different experimental conditions (bottom). Hierarchical clustering was calculated by Manhattan Distance. Colors indicate peptide intensities, ranging from low (red) to high (blue). (Graph generated with Spectronaut® v. 19)* ***Panel B,*** *Principal Component analysis (PCA) from LC-MS/MS run 2. PCs 1 & 2 contribute to the explanation of 56.08% of sample variability. Based on these two PCs, wild-type samples (circle) inhibitor- treated samples (diamond) and KO samples (square) formed 3 distinct groups, indicating that the replicates within each group share similarities with each other but there are significant differences in the immunopeptidomes between different treatments.* ***Panels C-E,*** *Volcano plots from LC-MS/MS run 2 indicating the statistical significance of the differences between (C) the inhibitor-treated and the wild type A375 cells, (D) the genetically modified (KO) and the wild type A375 cells and (E) the KO versus inhibitor-treated cells. Each circle represents a unique peptide sequence. Peptides with a q-value ≤0.05 and a log2 fold change ≥1 are considered statistically significant.*

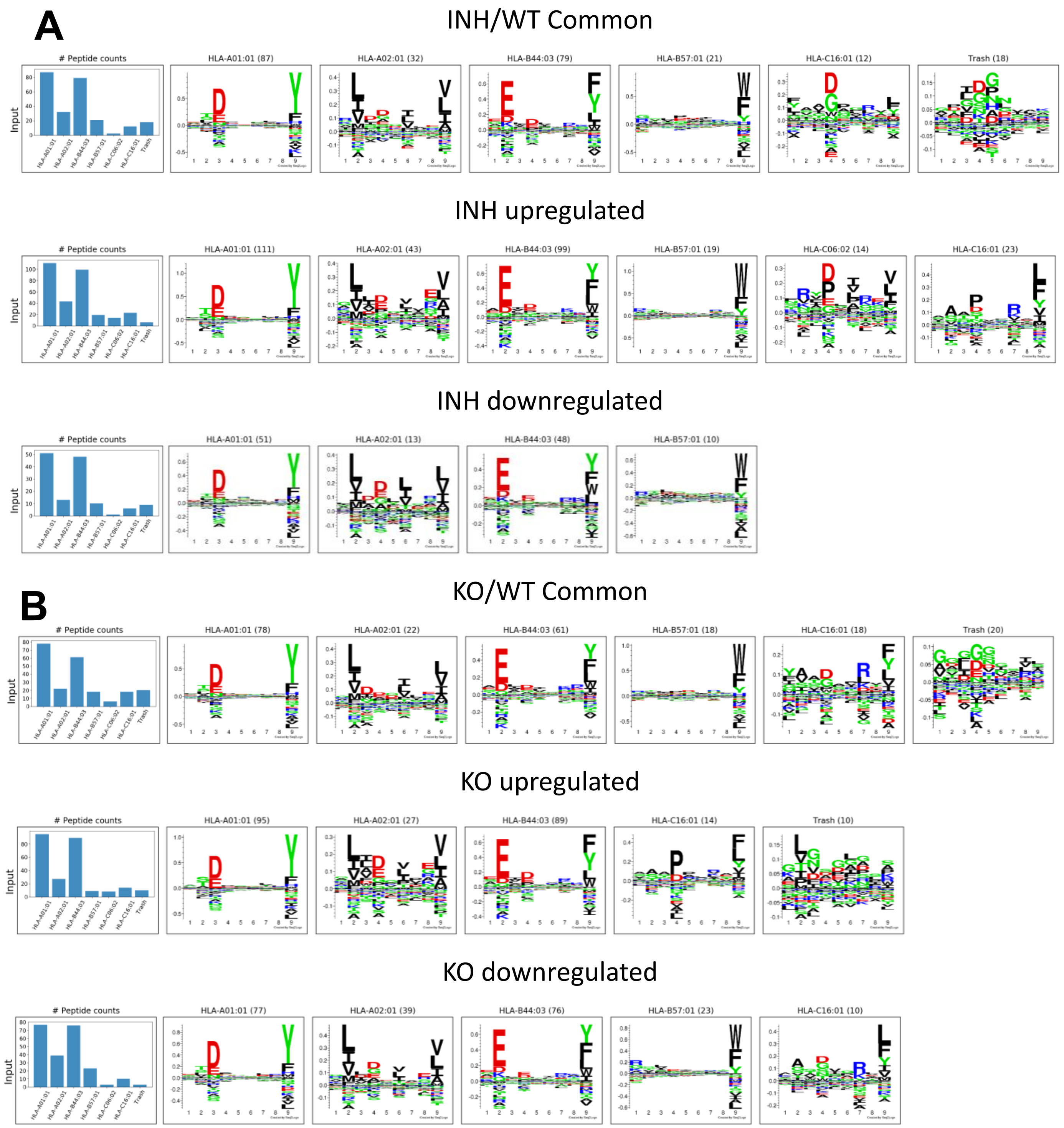

Figure S9: ***Panel A,*** *Motifs of the common, inhibitor upregulated and inhibitor downregulated peptides for the HLA alleles carried by A375 cells, as predicted by the MHC_Motif_Decon 1.0 server.* ***Panel B,*** *Motifs of the common, KO upregulated and KO downregulated peptides for the HLA alleles carried by A375 cells, as predicted by the MHC_Motif_Decon 1.0 server.*

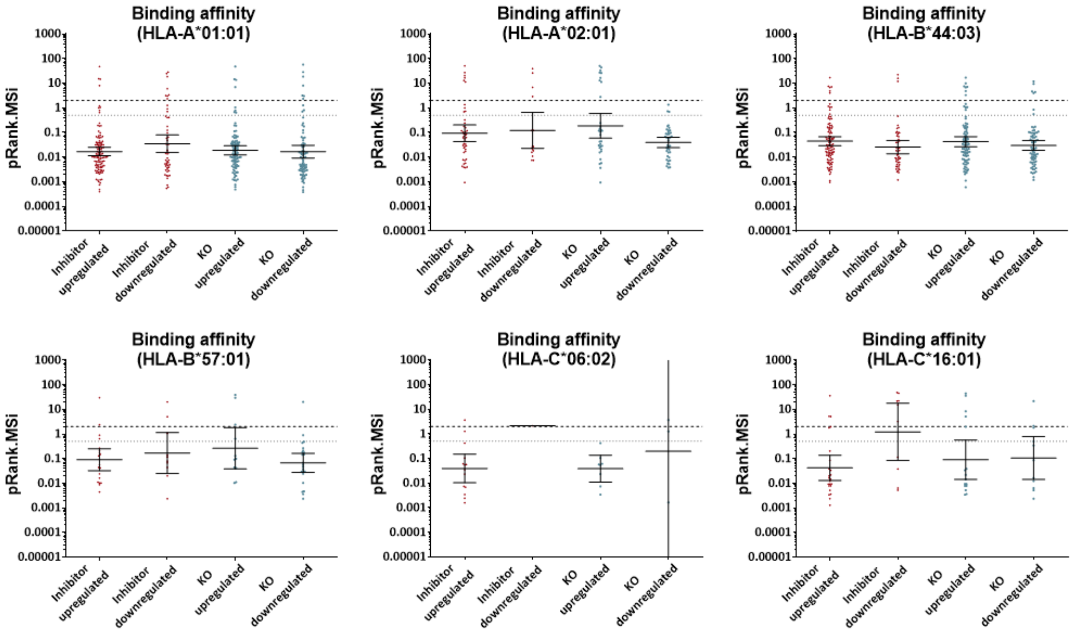

Figure S10: Distribution of predicted affinities (NetMHCpan-4.1) of the significantly up- or downregulated peptides after inhibitor treatment or ERAP1 KO for the HLA alleles present in A375 cells separately *(A*01:01, A*02:01, B*44:03, B*57:01, C*06:02, C*16:01). Each point on the graph signifies a distinct peptide sequence. Only the score for the top predicted HLA allele is depicted for each peptide.*

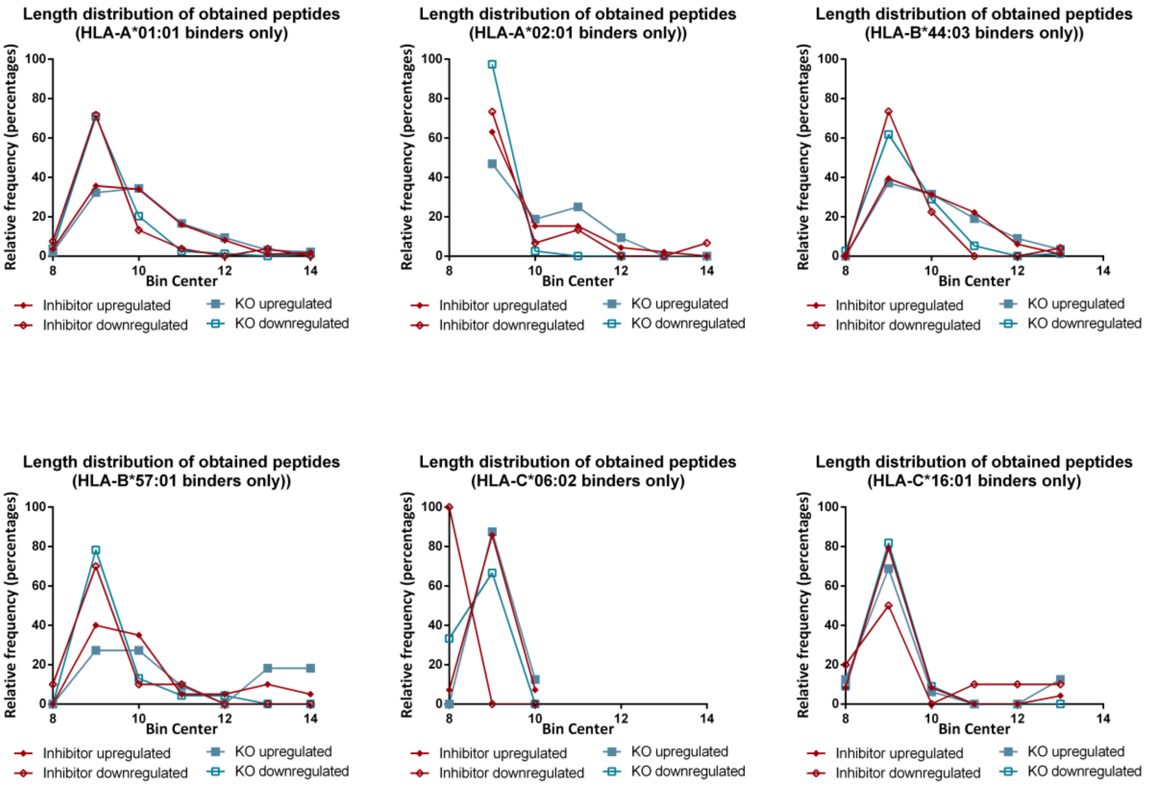

Figure S11: ***Length distribution per HLA allele of the significantly up- or downregulated binding peptides after inhibitor treatment or ERAP1 KO.***

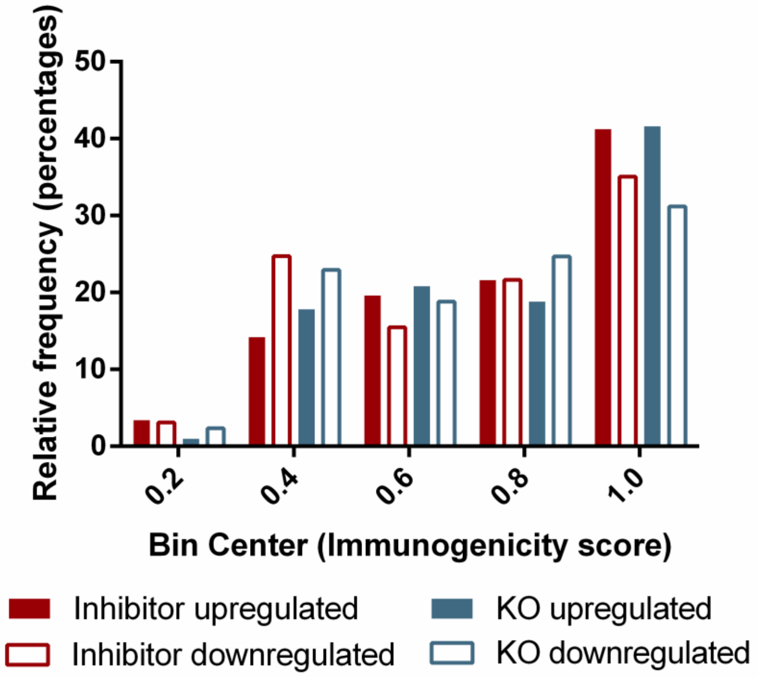

Figure S12: Distribution of the immunogenicity scores of the differentially expressed 9mers, obtained with the DeepImmuno algorithm.

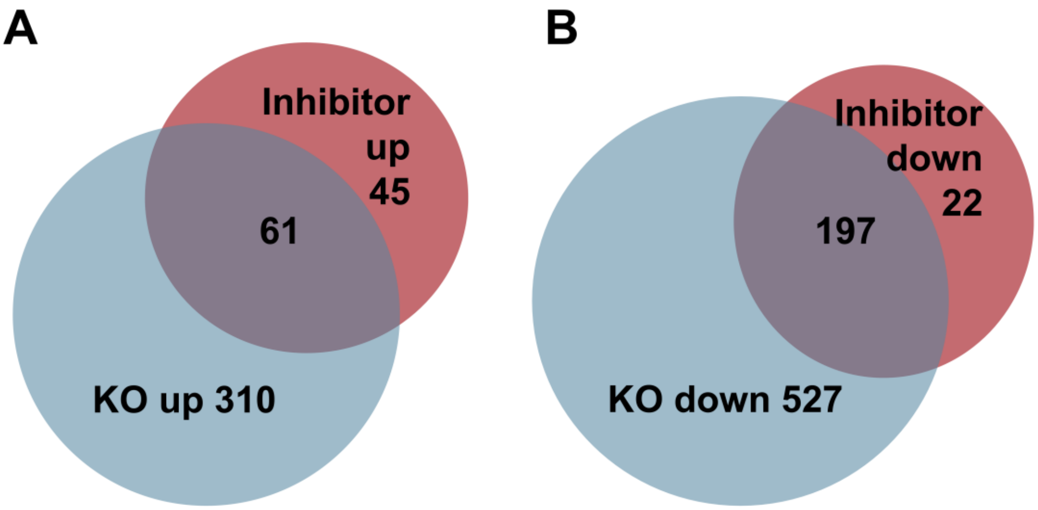

Figure S13: Venn diagrams, indicating the overlap of the significantly up-regulated (A) and down-regulated (B) proteins between the inhibitor-treated and the ERAP1 KO cells.

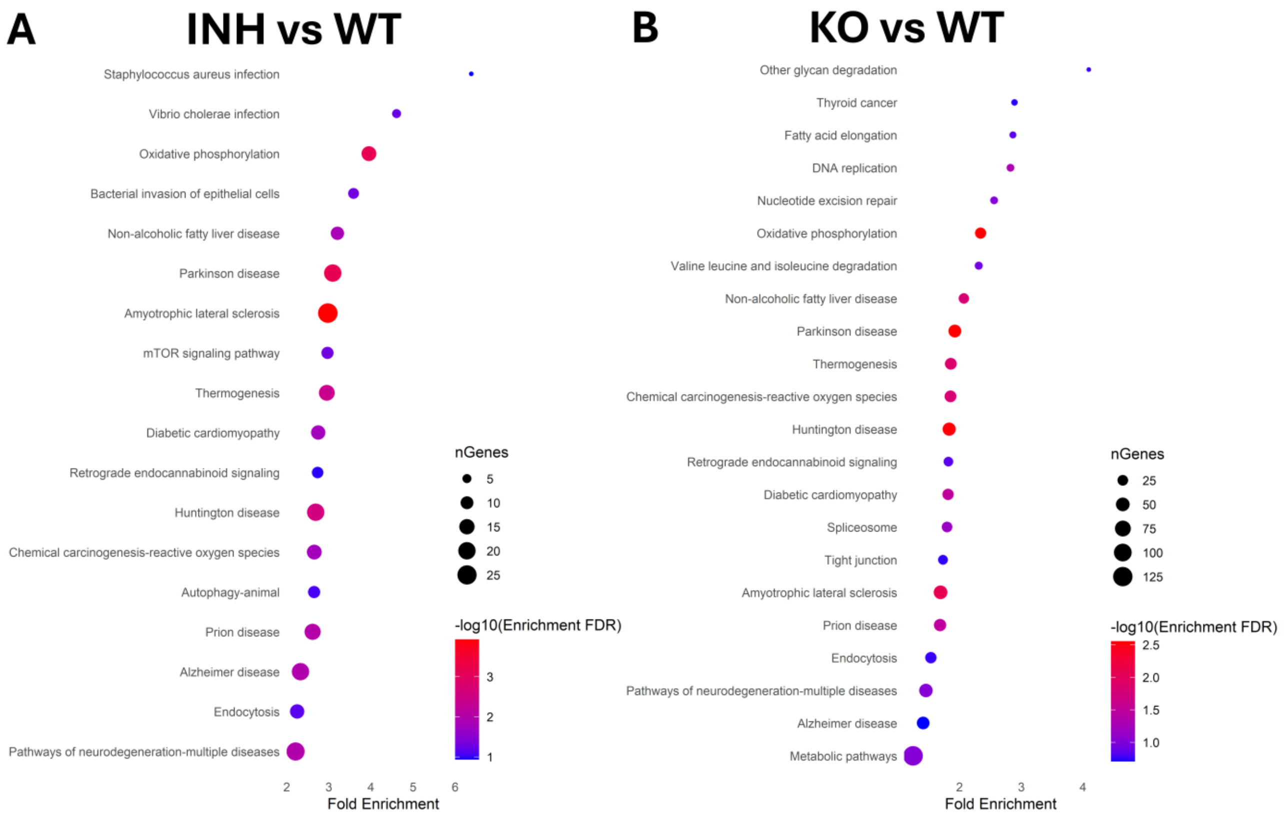

Figure S14: ShinyGo Pathway enrichment analysis using the KEGG database for A375 cells. *Enriched pathways from inhibitor affected (A) and KO affected (B) proteins ordered by fold-enrichment. Color indicates significance (FDR), while dot size indicates the number of affected genes in the given pathway.*

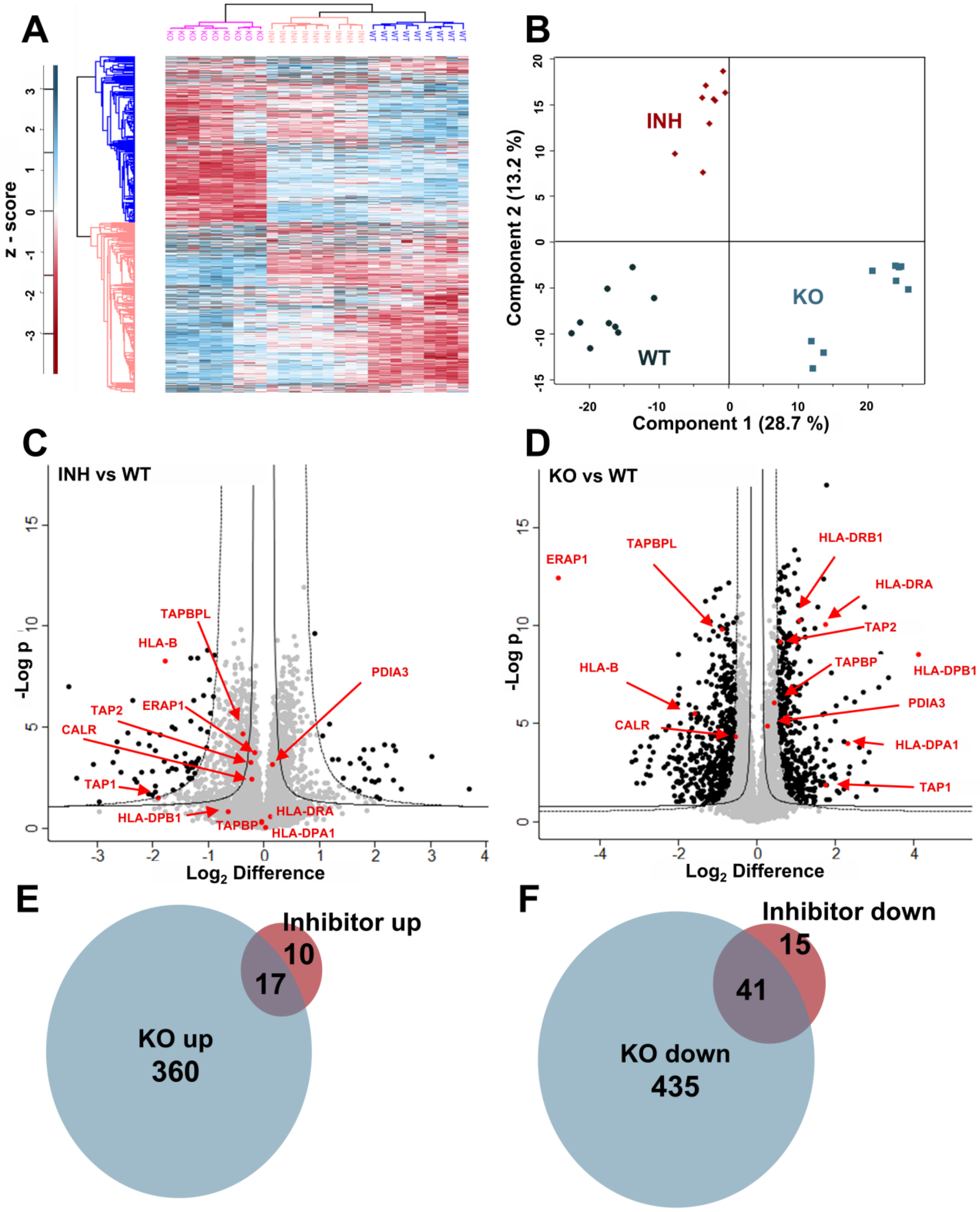

Figure S15: Proteomic analysis of wild-type, inhibitor-treated & ERAP1 KO THP-1 cells. Panel A, *Heatmap showing the distribution of proteins in the three conditions (WT, inhibitor-treated & ERAP1 KO THP-1 cells) for each replicate (three biological replicates, each measured in three technical replicates).* ***Panel B,*** *Principal Component Analysis (PCA) of the three experimental conditions. Each point represents an injection in the LC-MS/MS. All experimental conditions can be discerned from each other.* ***Panels C & D,*** *Hawaii plots, indicating the statistical significance of the observed differences in protein abundance between the two treatment conditions (inhibitor-treated & ERAP1 KO) and the WT*

cells. 83 proteins were differentially expressed in the inhibitor-treated (C) cells and 877 proteins were differentially expressed in the KO cells (D). Select proteins that participate in antigen presentation are indicated in red. ***Panels E & F,*** *Venn diagrams, indicating the overlap of the significantly up-regulated (E) and down-regulated (F) proteins between the inhibitor -treated and the ERAP1 KO cells.*

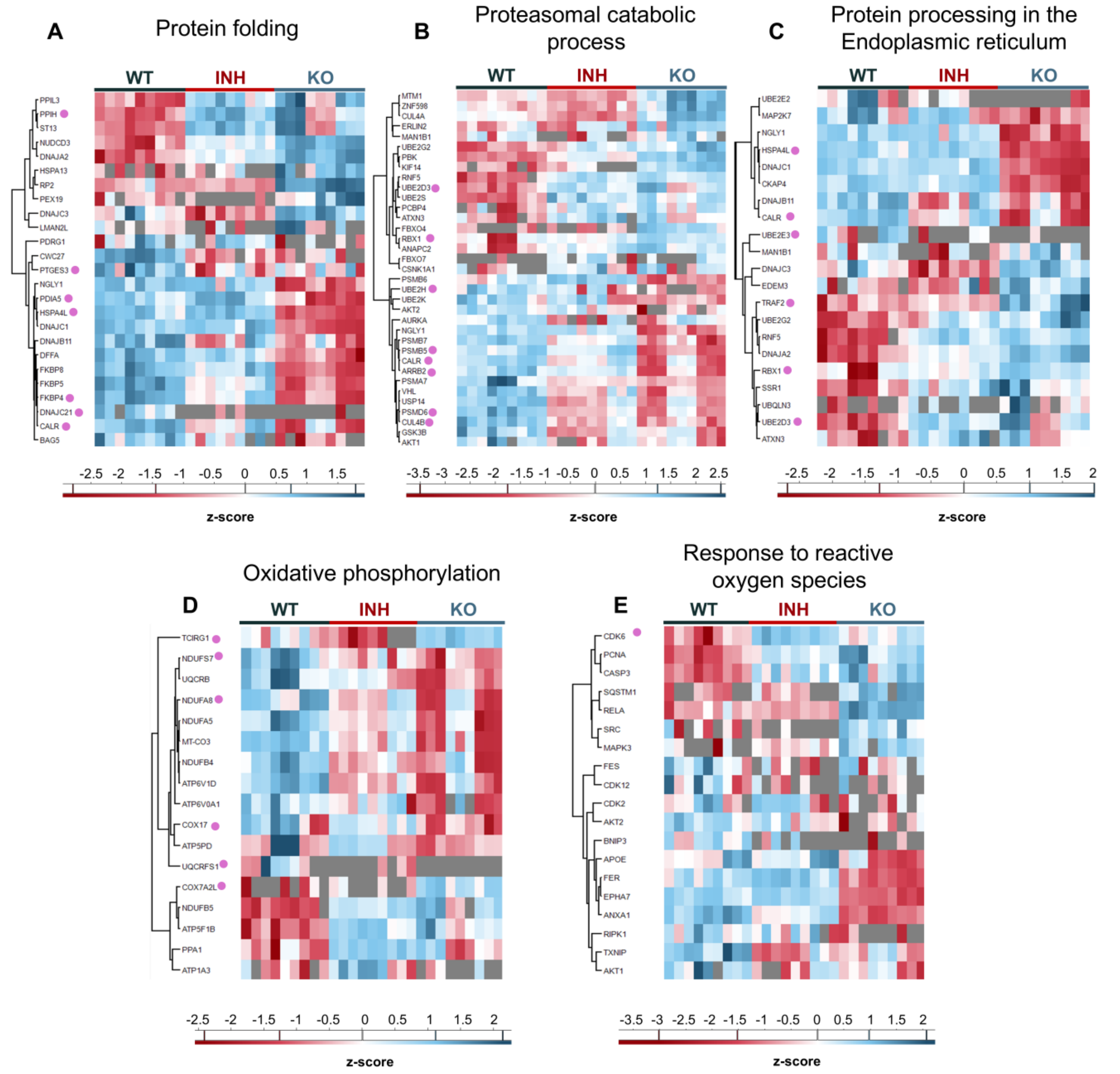

***Figure S16**: Heatmaps of selected processes in THP-1 cells, that were also affected in A375 cells.* *Proteins affected in both cell lines are highlighted with a pink dot (●).*

A   A375 cells Single Nucleotide Polymorphisms (SNPs) for ERAP1

rs2287987	
**Organism**	*Homo sapiens*
**Position**	chr5:96793832 (GRCh38.p14)
**Alleles**	T>A / T>C

t/M

Fv-T   Rv-A

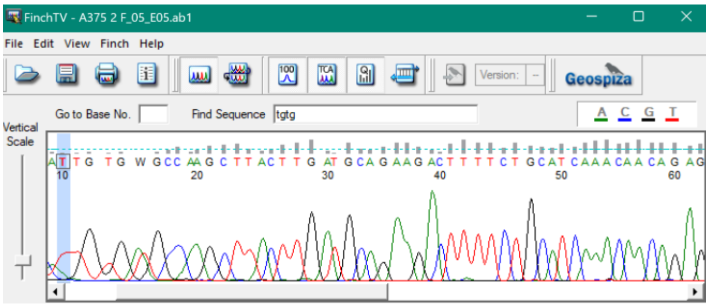

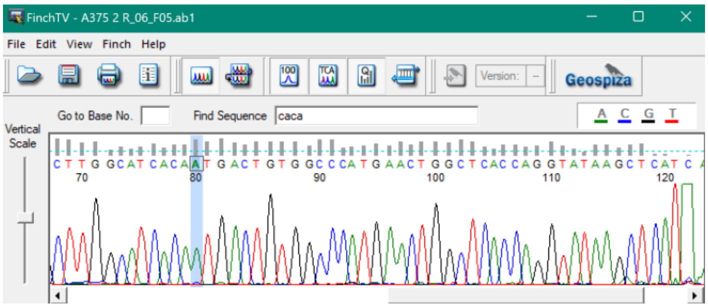

rs30187	
**Organism**	*Homo sapiens*
**Position**	chr5:96788627 (GRCh38.p14)
**Alleles**	T>A / T>C

t/K

Fv-T   Rv-A

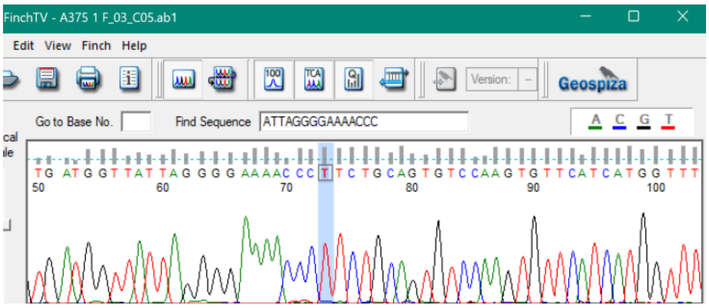

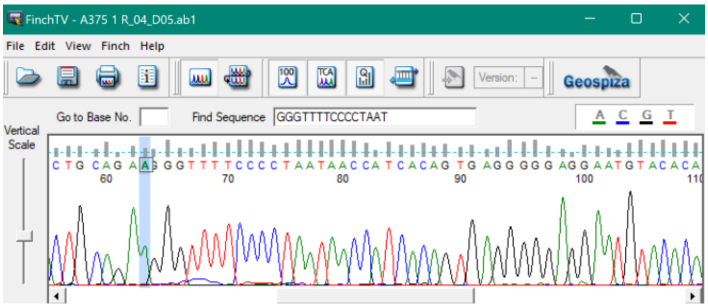

rs10050860	
**Organism**	*Homo sapiens*
**Position**	chr5:96786506 (GRCh38.p14)
**Alleles**	C>T

c/D

Fv-C   Rv-G

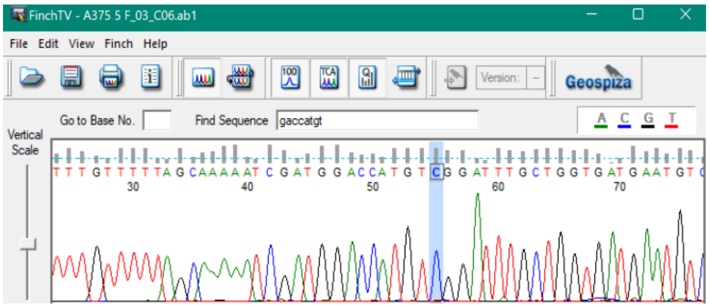

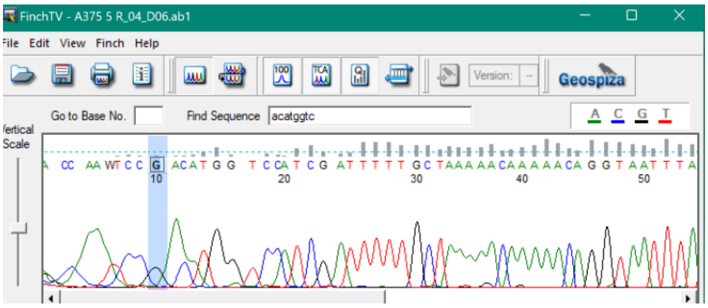

rs17482078

**Organism**	*Homo sapiens*
**Position**	chr5:96783162 (GRCh38.p14)
**Alleles**	C>G / C>T

c/R

Fv-C

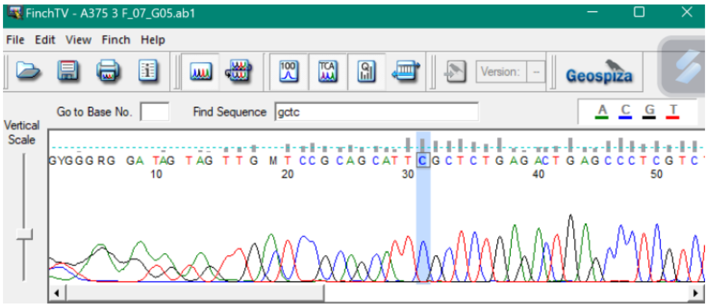

Rv-G

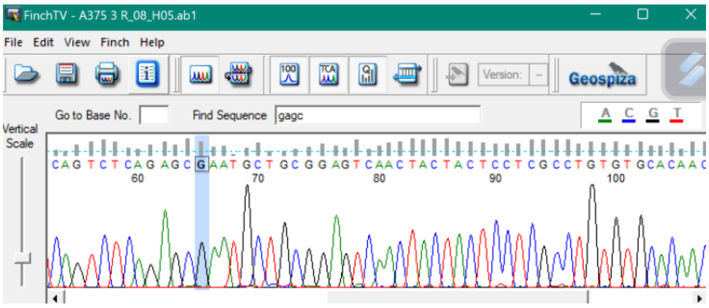

rs27044

**Organism**	*Homo sapiens*
**Position**	chr5:96783148 (GRCh38.p14)
**Alleles**	G>A / G>C

g/Q

Fv-G

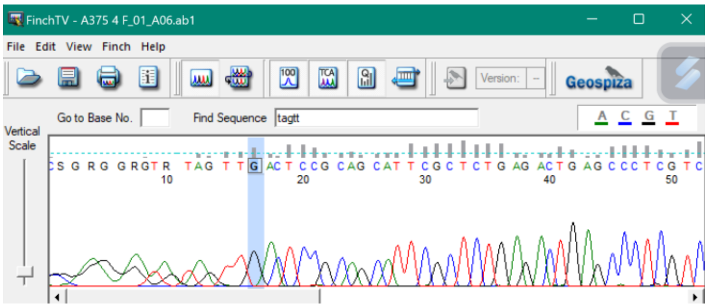

Rv-C

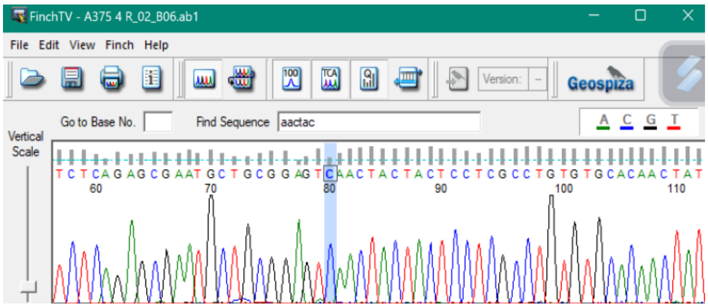

B THP-1 cells Single Nucleotide Polymorphisms (SNPs) for ERAP1

rs2287987

**Organism**	*Homo sapiens*
**Position**	chr5:96793832 (GRCh38.p14)
**Alleles**	T>A / T>C

t/M

Fv-T

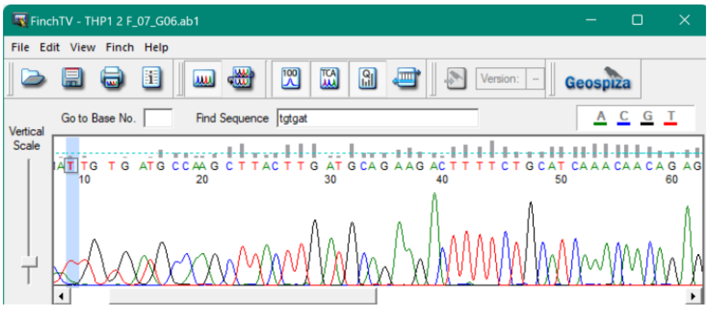

Rv-A

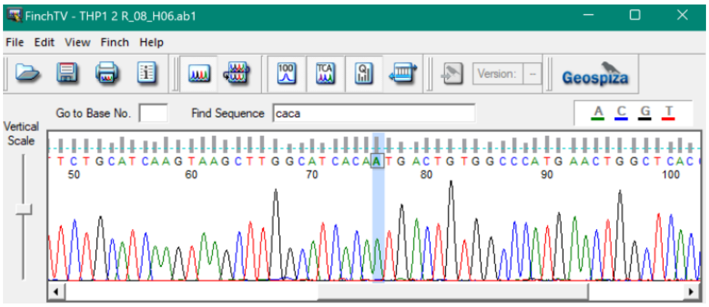

rs30187

**Organism**	*Homo sapiens*
**Position**	chr5:96788627 (GRCh38.p14)
**Alleles**	T>A / T>C

t/K & R

Fv-C/T

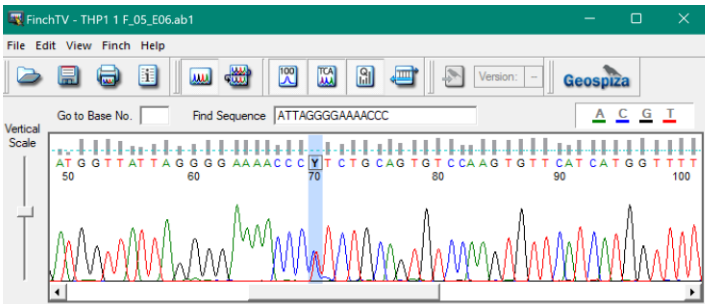

Rv-A/G

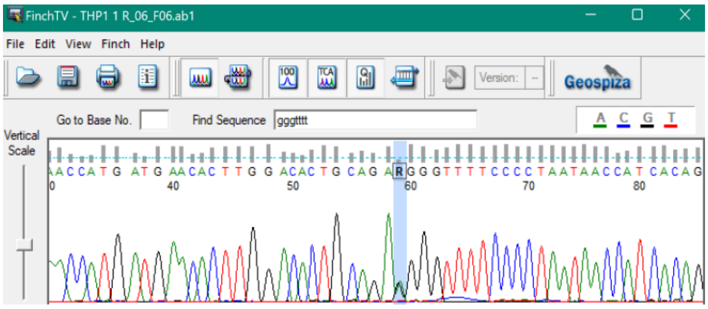

rs10050860

**Organism**	*Homo sapiens*
**Position**	chr5:96786506 (GRCh38.p14)
**Alleles**	C>T

c/D

Fv-C

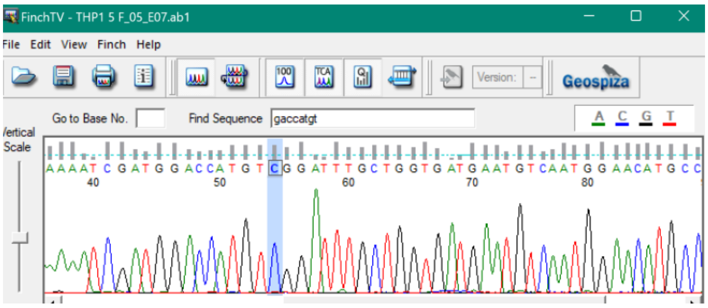

Rv-?

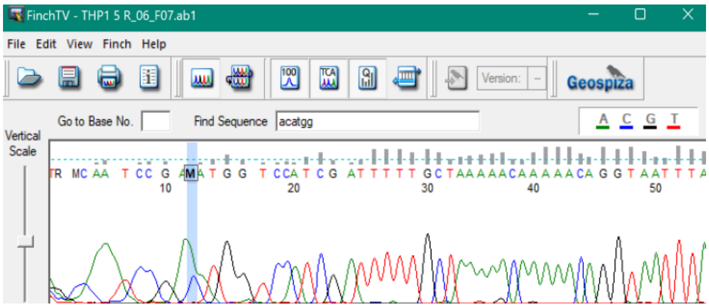

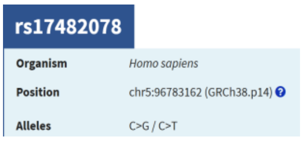

c/R

Fv-C

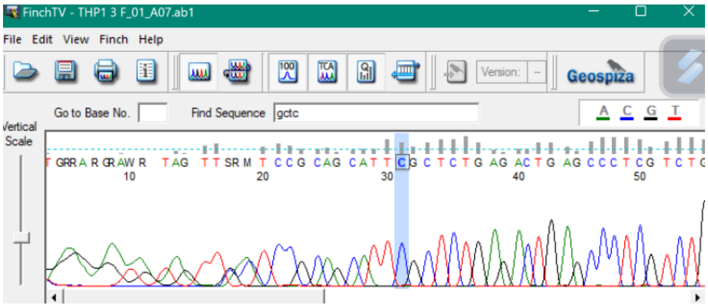

Rv-G

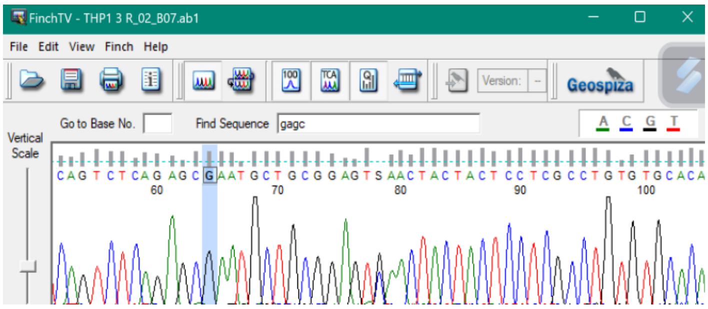

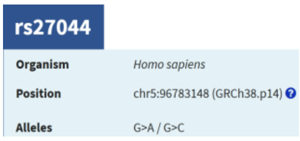

g/Q & E

Fv-G/C

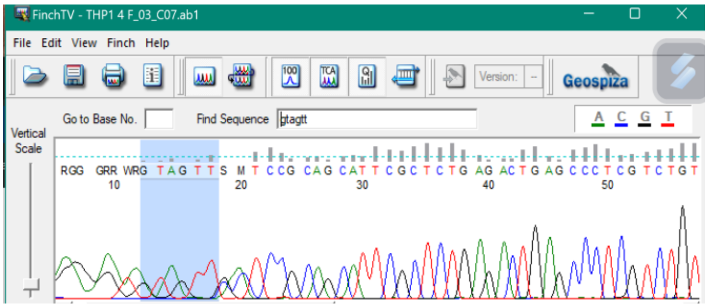

Rv-C/G

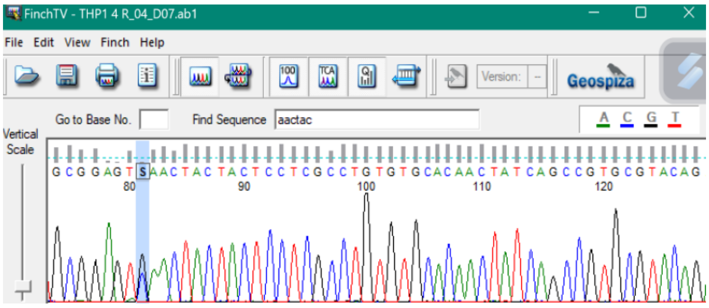

Figure S17: Sequencing results for ERAP1 Single Nucleotide polymorphisms in A375 (A) and THP-1 (B)

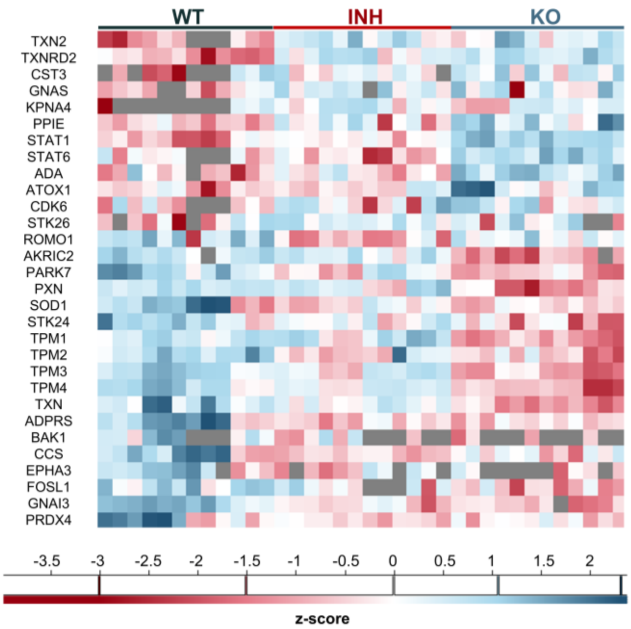

Figure S18: Heatmap of proteins related to response to reactive oxygen species (A375 cells).

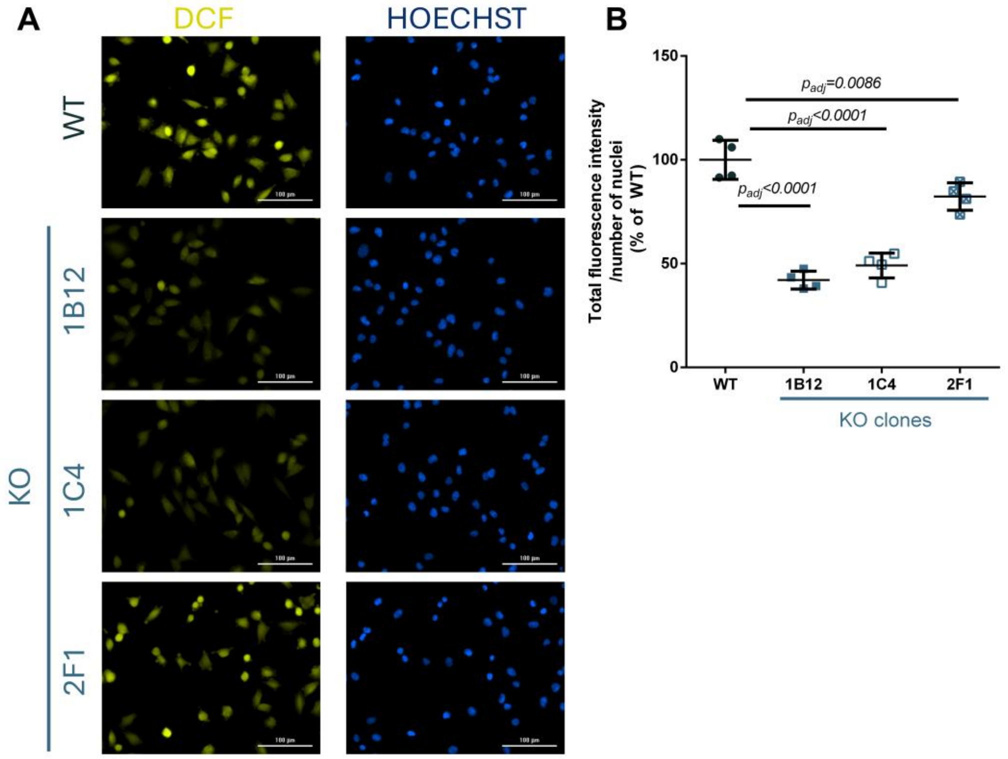

***Figure S19**: **Dichlorofluorescein (DCF) assay for measurement of reactive oxygen species (ROS). Panel A,** representative images of wild-type (WT) and three clones of ERAP1 KO (KO) A375 cells incubated with H2DCFDA (left) and Hoechst 33342 (right). **Panel B,** total fluorescence intensity per number of nuclei quantitated by direct cell imaging on a Cytation-5 instrument for the four biological conditions. The calculated adjusted p values comparing KO cells to the wild-type cells are indicated. Statistical significance was evaluated by one-way ANOVA, followed by Dunnett's multiple comparisons test in GraphPad Prism v.8. For this experiment A375 cells were seeded in black wall, transparent flat-bottom plates at a density of 15,000 cells/well. 20 hours later the medium was removed and 25 μM DCF together with 1 μg/ml Hoechst 33342 were added as described in the methods section. Each dot represents the average of four images obtained per well.*

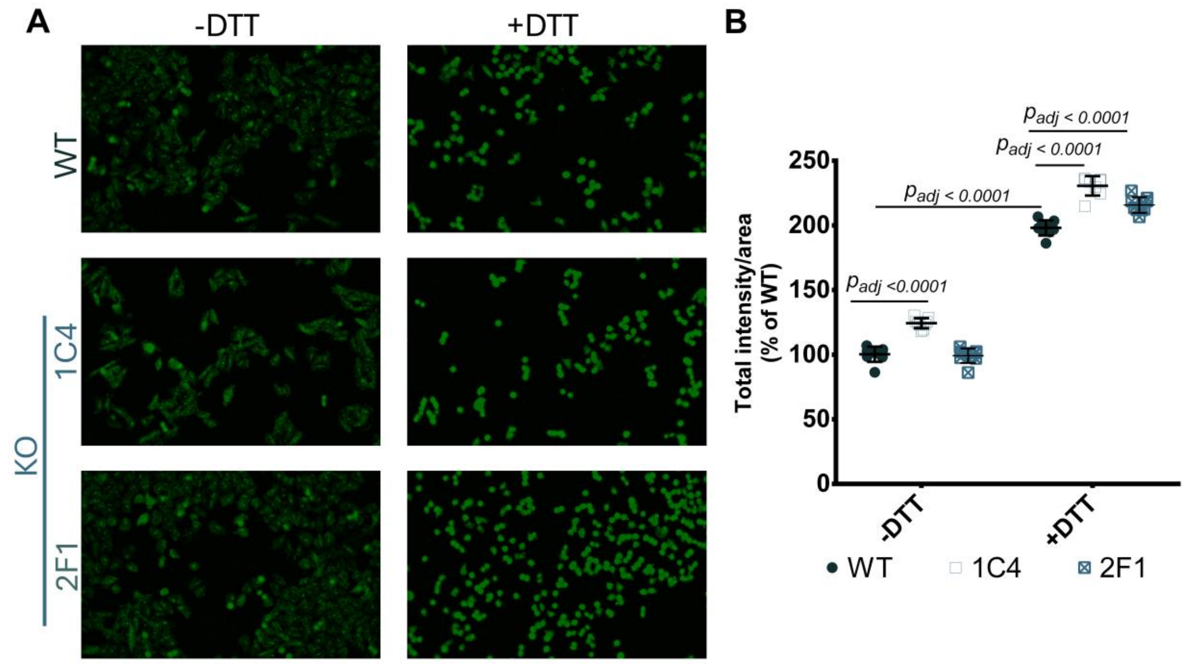

Figure S20: Thioflavin T assay for ER stress*. Panel A,** *representative images of wild-type (WT), and ERAP1 KO (KO) A375 cells (clones 1C4 and 2F1) incubated with Thioflavin T, with and without treatment with DTT, as described in the Experimental Methods section.* ***Panel B, *total fluorescence intensity normalized for cell area for the three biological conditions in the absence or presence of DTT. Statistical significance was evaluated by one-way ANOVA, followed by Dunnett's multiple comparisons test in GraphPad Prism v.8.*

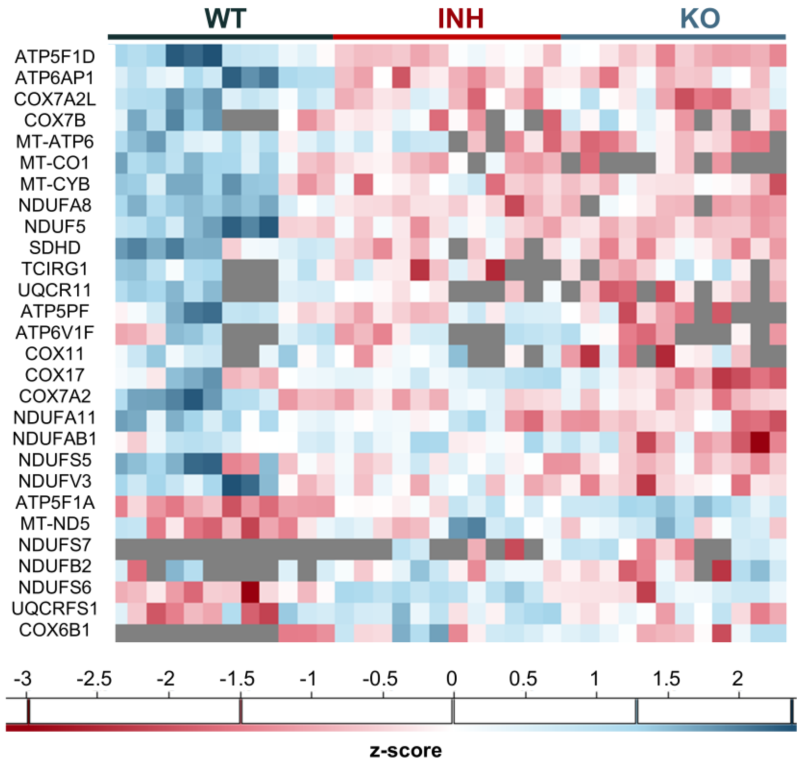

Figure S21: *Heatmap of proteins related to oxidative phosphorylation (A375 cells).*

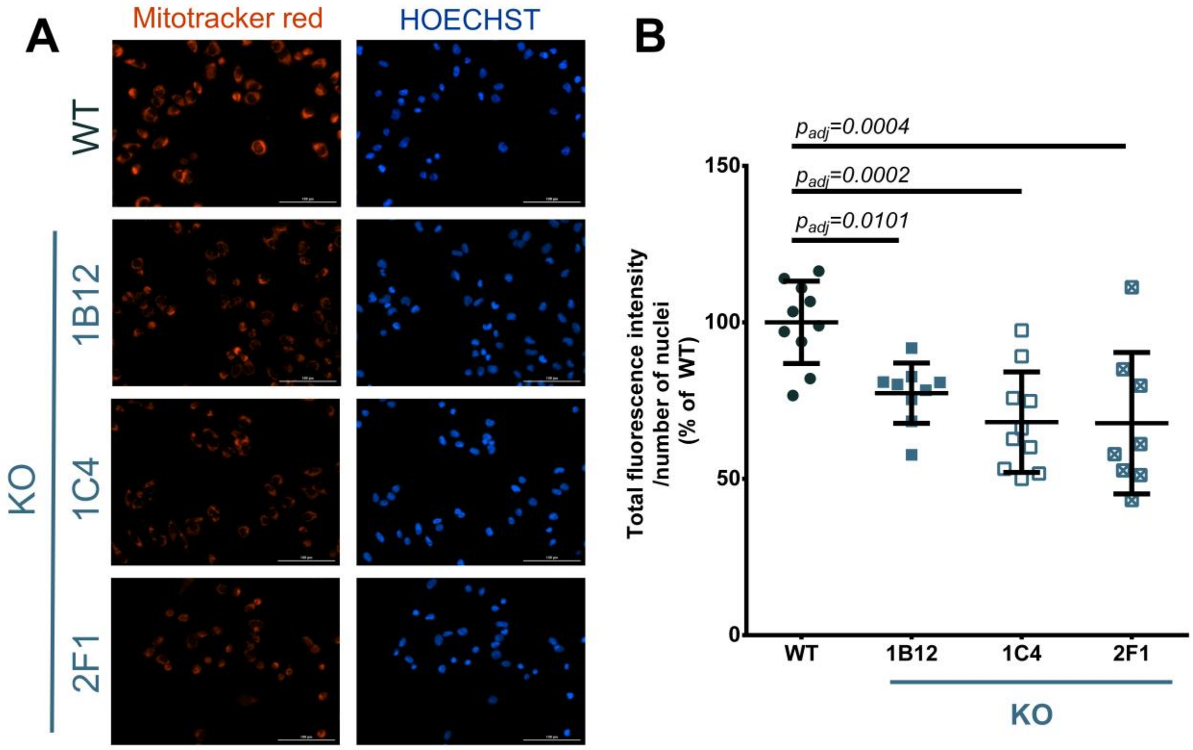

***Figure S22: Mitotracker assay for mitochondrial membrane potential. Panel A**, representative images of wild-type (WT) and three clones of ERAP1 KO (KO) A375 cells incubated with Mitotracker (red) and Hoechst 33342 (right). **Panel B**, total fluorescence intensity of Mitotracker per identified nuclei for the four conditions. The calculated adjusted p values comparing KO cells to the wild-type cells are indicated. Statistical significance was evaluated by one-way ANOVA, followed by Dunnett's multiple comparisons test in GraphPad Prism v.8. For this experiment, A375 cells were seeded in black wall, transparent flat-bottom plates at a density of 15,000 cells/well. 20 hours later the medium was removed and 250 nM Mitotracker together with 1 µg/ml Hoechst 33342 were added. Measurements were obtained in live cells prior to fixation. Each dot represents the average of four images obtained per well.*

Supplemental Table S1: Identified peptides corresponding to known MAGE antigens. Annotated spectra of the peptides are included in the respective section below.

MAGE antigens			
MAGE	Sequence	KO	INH
A3	EVDPIGHLY		
A4	AETSYVKVL	Upregulated	Upregulated
A4	EVDPASNTY		
A4	GSNPARYEF	Upregulated	Upregulated
A4	KEVDPASNTY	Downregulated	
A4	TVYGEPRKL	Upregulated	Upregulated
A11	EVDPTSHSY	Downregulated	Downregulated
C2	EEVPSGVIPNL	Upregulated	Upregulated
C2	FVYGEPREL	Upregulated	Upregulated
(red)	Downregulated		
(blue)	Upregulated		

Supplemental Table S2: Identified peptides corresponding to known antigenic epitopes in the IEDB database. Annotated spectra of the peptides are included in the respective section below.

Epitopes from IEDB			
Sequence	Protein Name	KO	INH
LLDVPTAAV	Gamma-interferon-inducible lysosomal thiol reductase precursor	Upregulated	
KLDVGNAEV	B-cell receptor-associated protein 31	Downregulated	Upregulated
RLFDEPQLA	BTB/POZ domain-containing protein 2	Downregulated	
TLWVDPYEV	Protein BTG1		
TMLARLASA	Chondroitin sulfate proteoglycan 4	Downregulated	Upregulated
GLIEKNIEL	DNA (cytosine-5)-methyltransferase 1	Downregulated	Upregulated
LLDLDEELRY	Chromosome 14 open reading frame 179	Upregulated	Upregulated
EVDPIGHLY	MAGEA3		
FVYGEPREL	MAGEC2	Upregulated	Upregulated
SIIGRLLEV	serine/threonine-protein phosphatase PP1-alpha catalytic subunit isoform 1		
IMLEALERV	Small nuclear ribonucleoprotein G (snRNP-G) (Sm protein G)	Downregulated	Downregulated
SEEEFLRTY	Beta-catenin-interacting protein 1	Downregulated	
(red)	Downregulated		
(blue)	Upregulated		

Supplemental Table S3: Identified peptides originating from unannotated proteins. Annotated spectra of the peptides are included in the respective section below.

Unannotated proteins				
Sequence	Length	PG.ProteinGroups	KO	INH
AEEPLAGRTW	10	GN=POLD4 3' Overlap dORF	Downregulated	Downregulated
ALAAVVTEV	9	GN=DDX3X 3' dORF	Upregulated	Upregulated
EEAVVLRGL	9	GN=WBP1 5' uORF	Downregulated	
EEYRGGNNW	9	GN=TTC37 5' uORF		Downregulated
FSNDALKTY	9	GN=FAM127B 3' Overlap dORF	Upregulated	
IEVDGGRDW	9	GN=SLC39A7 5' uORF		
IFSQRSYSY	9	GN=NFAT5 Out-of-Frame	Downregulated	Downregulated
ISDPGVQGY	9	GN=IKBKAP 5' Overlap uORF	Downregulated	Downregulated
LVSNGVLVV	9	GN=PLA2G4B 3' Overlap dORF		
RTAAAAAGAASW	12	GN=URI1 5' uORF	Downregulated	Upregulated
RTDQFYVVY	9	GN=PTGES3 ncRNA Processed Transcript		
SAAWDRPPL	9	GN=MPG Out-of-Frame		Upregulated
STSTILRSF	9	GN=GLCE ncRNA Processed Transcript	Downregulated	
TDGVSLLLP	9	GN=TFB1M 5' Overlap uORF		Downregulated
TDNRTDIFY	9	GN=nan lincRNA		
VEDPIAEGGR	10	GN=RUBCN Truncated	Upregulated	Upregulated
VTEKVYADTGLY	12	GN=TMEM168 5' uORF	Upregulated	Upregulated
(red)	Downregulated			
(blue)	Upregulated			

Supplemental Table S4: Amino acids at SNPs for ERAP1

Cell line \ SNP	349	528	575	725	730
**A375**	M	K	D	R	Q
**THP-1**	M	R-K	D	R	Q-E

SUPPLEMENTAL METHODS

Isolation of genomic DNA

A375 wild type and two clones of ERAP1 silenced cells (1G5 and 1B12) were washed twice with ice-cold PBS and incubated overnight at 37 °C with lysis buffer (10 mM NaCl, 10 mM Tris–HCl pH 7.5, 10 mM EDTA, 0.5% SDS and 100 µg/ml RNAse A and 100 µg/ml proteinase K). Genomic DNA was isolated by phenol/$CHCl_3$ extraction and precipitated with 1/10 vol of 3 M sodium acetate, pH 5.2, and 2 vol of 100% ethanol. The pellet was washed with 70% ethanol, re-suspended in sterile milli-Q H_2O and quantitated based on absorption at 260/280 nm.

High-density SNP-array analysis

Genomic DNA from A375 wild type and ERAP1-silenced cells was used for SNP-array copy number profiling and analysis of regions of homozygosity with the Infinium Human CytoSNP-850K v1.2 BeadChip (Illumina, San Diego, CA, USA). This array has approximately 850,000 single nucleotide polymorphism (SNP) markers spanning the genome and can detect genomic insertions, deletions, and regions of homozygosity. Data analysis was performed with NxClinical software v6.0 (Bionano genomics, San Diego, CA, USA). Human genome build Feb. 2009 GRCh37/hg19 was used and results were classified with BENCH Lab CNV software (Agilent, Santa Clara, CA, USA).

MTT cytotoxicity assay

Wild-type A375 cells were seeded in a 96-well plate at a density of 50,000 cells/ml. 24 hours later, cells were treated with the inhibitor at a concentration range between 0 and 100 µM in the presence of 0.5% DMSO. Cells were incubated for 48 hours at 37 °C, 5% CO_2. The medium was replaced with 100 µl fresh medium containing 1 mg/ml 3-(4,5-Dimethylthiazol-2-yl)-2,5-Diphenyltetrazolium Bromide (MTT) and the plate was incubated for 4 hours. MTT containing medium was removed, 100 µl dimethylsulfoxide (DMSO) per well were added and the plate was shaken for 15 minutes prior to measurement of absorbance at 540 and 620 nm.

Analysis of surface MHC I expression

On treatment day 6, ca. 10^6 cells were detached from the wells of a 12-well plate with 10 mM Ethylenediamine tetraacetic acid (EDTA) in Phosphate-buffered saline (PBS). Cells were washed with PBS and FACS buffer (1% Bovine serum albumin, 0.02% Sodium azide in PBS) prior to incubation with the Mouse, anti-human HLA-ABC FITC labelled antibody, diluted 1:25 (Biorad, MCA81F). A negative control was also prepared by incubating with FACS buffer without the addition of the antibody. The incubation took place for 30 minutes on ice and the cells were protected from light. Subsequently, cells were washed twice with FACS buffer, resuspended in 100 µl FACS buffer- 1% Formic Acid 1:1 and transferred to FACS tubes for the analysis with a Cytomics™ FC 500 (Beckman Coulter) cytometer working with the CXP Analysis software (v 2.2.).

Identification of Single Nucleotide Polymorphisms (SNP) by sequencing ERAP1 and ERAP2

Primers for sequencing ERAP1 and ERAP2 SNPs were designed using the NCBI Primer-BLAST tool based on ERAP1 GeneBank RefSeq (NG_027839.2) and ERAP2 (NG_051092.1) (Krol et al. in preparation). The reaction conditions include denaturation (95°C/2 min), thermocycling (92°C/30 sec; 60°C/1 min; 72°C/1 min for 35 cycles), extension (72°C/1 min). The annealing temperature for rs2248374, rs2287987, rs17482078 was 60°C and for rs30187, rs27044 and rs10050860 was 64°C. PCR products were analyzed by gel electrophoresis, purified from the gel and sequenced. Sequences were visualized and analyzed using the FinchTV 1.4 chromatogram viewer.

Gene	SNP variant	Primer-Fv (5'->3')	Primer-Rv (5'->3')
**ERAP1**	rs2287987	ATGAGCTTATACCTGGTGAGC	CCGACTTTCAGTCTGGTGCT
**ERAP1**	rs30187	CATTACCCAGTGTCCGGGG	AAATGGGTGATGTGTCTGCC
**ERAP1**	rs10050860	ACACATTTTCACATTCCTCCTTGAA	GGTACCTGTGGCATGTTCCAT
**ERAP1**	rs17482078	CTGTACGCACGGCTGATAGT	GTTTCCCTGTACAACGCCCT
**ERAP1**	rs27044	CTCTGTACGCACGGCTGATA	CTGTTTCCCTGTACAACGCC
**ERAP2**	rs2248374	CATTCGGATCCCAAGATGAC	GGAGTGAACACCCGTCTTGT

Spectronaut settings

Settings Used: BGS Factory Settings

```
 ├─ DIA Analysis\Calibration
 |  ├─ MZ Extraction Strategy:        Maximum Intensity
 |  ├─ Precision iRT:  True
 |  |  ├─ Exclude De-amidated Peptides:     True
 |  |  └─ iRT <-> RT Regression Type: Local (Non-Linear) Regression
 |  ├─ MS1 Mass Tolerance Strategy:          System Default
 |  └─ MS2 Mass Tolerance Strategy:System Default
 ├─ DIA Analysis\Identification
 |  ├─ Precursor Qvalue Cutoff:       0.01
 |  ├─ Precursor PEP Cutoff: 0.2
 |  ├─ Protein Qvalue Cutoff (Experiment):  0.01
 |  ├─ Protein Qvalue Cutoff (Run):  0.05
 |  ├─ Protein PEP Cutoff:     0.75
 |  ├─ Single Hit Definition:  By Stripped Sequence
 |  ├─ Exclude Single Hit Proteins:    False
 |  ├─ Exclude Duplicate Assays:       True
 |  ├─ Exclude Predicted Fragment Scores:  False
 |  ├─ Generate Decoys:       True
 |  |  ├─ Decoy Generation Method:           Mutated
 |  |  |  └─ Preferred Fragment Source:       NN Predicted Fragments
 |  |  └─ Decoy Limit Strategy:         Dynamic
 |  |     └─ Library Size Fraction:        0.1
```

```
|  └─ Pvalue Estimator:        Kernel Density Estimator
├─ DIA Analysis\Pipeline Mode
|  ├─ Export All XICs:         False
|  ├─ Generate SNE File:       True
|  |  └─ Store Ion traces in SNE:       True
|  ├─ Post Analysis Reports:
|  |  ├─ Binned CVs: False
|  |  ├─ Binned Identification:         False
|  |  ├─ CV Density Line Chart:         False
|  |  ├─ CVs Below X Bar Chart:         False
|  |  ├─ Data Completeness Bar Chart:          False
|  |  ├─ Run Identifications Bar Chart:        False
|  |  └─ Scoring Histograms: False
|  ├─ Report Schema:           BGS Factory Report (Normal)
|  └─ Reporting Unit:          Across Experiment
├─ DIA Analysis\Post Analysis
|  ├─ Differential Abundance Testing:          Unpaired t-test
|  |  ├─ Assume Equal Variance:         False
|  |  ├─ Group-Wise Testing Correction:        False
|  |  ├─ Log2 Ratio Candidate Filter:          0.58
|  |  └─ Confidence Candidate Filter:          Qvalue
|  |     └─ Confidence:        0.05
|  ├─ Differential Abundance Grouping:         Minor Group (Quantification Settings)
|  |  └─ Smallest Quantitative Unit: Minor Group (Quantification Settings)
|  |     └─ Use All MS-Level Quantities:       True
|  ├─ Calculate Explained TIC:          None
|  ├─ Calculate Sample Correlation Matrix:False
|  └─ Hierarchical Clustering:          True
|     ├─ Distance Metric:      Manhattan Distance
|     ├─ Linkage Strategy:     Ward's Method
|     ├─ Order Runs by Clustering:      True
|     └─ Z-score Transformation:        True
├─ DIA Analysis\Protein Inference
|  └─ Protein Inference Workflow:       Automatic
```

```
|    └─ Inference Algorithm: IDPicker
├─ DIA Analysis\PTM Workflow
|  ├─ [Beta] Input Normalization Strategy:  None
|  └─ PTM Localization:        False
├─ DIA Analysis\Quantification
|  ├─ Precursor Filtering:     Identified (Qvalue)
|  |  ├─ Imputation Strategy:          None
|  |  └─ Multi Channel Qvalue Filter:          Group Qvalue
|  ├─ Proteotypicity Filter:  None
|  ├─ Protein LFQ Method:  Automatic
|  ├─ Quantity MS Level:      MS2
|  ├─ Quantity Type:          Area
|  ├─ Cross-Run Normalization:      True
|  |  ├─ Normalization Filter Type:  None
|  |  ├─ Normalization Strategy:     Automatic
|  |  └─ Row Selection:        Automatic
|  |     └─ Multi Channel Qvalue Filter:          Group Qvalue
|  ├─ Quantification window:        Synchronized
|  ├─ Interference Correction:      True
|  |  ├─ Only Identified Peptides:   True
|  |  ├─ Exclude All Multi-Channel Interferences:   True
|  |  ├─ MS1 Min:    2
|  |  └─ MS2 Min:    3
|  ├─ Major (Protein) Grouping:     by Protein Group Id
|  ├─ Minor (Peptide) Grouping:     by Stripped Sequence
|  ├─ Major Group Quantity:         Mean peptide quantity
|  ├─ Major Group Top N:   True
|  |  ├─ Max:          3
|  |  └─ Min: 1
|  ├─ Minor Group Quantity:         Mean precursor quantity
|  ├─ Minor Group Top N:   True
|  |  ├─ Max:          3
|  |  └─ Min: 1
|  └─ DeepQuant Correction [Beta]: False
```

```
├─ DIA Analysis\Workflow
|  ├─ Method Evaluation:    False
|  ├─ Profiling Strategy:       None
|  ├─ Run Limit for directDIA Library:          -1
|  ├─ Hybrid (DDA + DIA) Library:    False
|  └─ Unify Peptide Peaks Strategy: None
├─ DIA Analysis\XIC Extraction
|  ├─ XIC IM Extraction Window:    Dynamic
|  |  └─ Correction Factor:    1
|  ├─ XIC RT Extraction Window:    Dynamic
|  |  └─ Correction Factor:    1
|  ├─ MS1 Mass Tolerance Strategy:           Dynamic
|  |  └─ Correction Factor:    1
|  └─ MS2 Mass Tolerance Strategy:Dynamic
|     └─ Correction Factor:    1
├─ Pulsar Search\Identification
|  ├─ PSM FDR:        0.05
|  ├─ Peptide FDR:   0.05
|  ├─ Protein Group FDR:    1
|  ├─ directDIA Workflow:   directDIA+ (Deep)
|  |  └─ RT Sampling Reduction:       1
|  └─ PTM Localization Filter:          False
├─ Pulsar Search\Labeling
|  └─ Channels:
|     ├─ Channel 1:    False
|     ├─ Channel 2:    False
|     └─ Channel 3:    False
├─ Pulsar Search\Modifications
|  ├─ Max Variable Modifications:   5
|  └─ Select Modifications:
|     ├─ Fixed Modifications::
|     └─ Variable Modifications::        Acetyl (Protein N-term), Oxidation (M)
├─ Pulsar Search\Peptides
|  ├─ Enzymes / Cleavage Rules:
```

```
| ├─ Digest Type:    Unspecific
| ├─ Max Peptide Length:  25
| ├─ Min Peptide Length:  7
| ├─ Missed Cleavages:    2
| └─ Toggle N-terminal M:  True
├─ Pulsar Search\Result Filters
| ├─ Fragment Ions:
| | ├─ Ion AA Length:     True
| | | └─ N: 3
| | ├─ Ion Charge: False
| | ├─ Ion Loss Type:     False
| | ├─ Ion Type:    False
| | ├─ m/z :        True
| | | ├─ Max:       3000
| | | └─ Min:       200
| | ├─ Overlapping between Channels:    False
| | └─ Relative Intensity:   True
| |    └─ Min:       1
| └─ Precursors:
|   ├─ Amino Acids:        False
|   ├─ Best N Fragments per Peptide:      True
|   | ├─ Max:       6
|   | └─ Min:       3
|   ├─ Best N Peptides per Protein Group: False
|   ├─ Channel Count:      False
|   ├─ FASTA Matched:      False
|   ├─ Missed Cleavage:    False
|   ├─ Modifications:      None
|   ├─ Peptide Charge:     False
|   └─ Proteotypicity:     False
├─ Pulsar Search\Speed-Up
| ├─ MS2 Index:    Automatic
| └─ diaPASEF Pre-Processing:      Fast (Spectronaut 19)
├─ Pulsar Search\Tolerances
```

```
| └─ Tolerance Parameters:
|    ├─ Thermo IonTrap:
|    |  ├─ Calibration Search:        Dynamic
|    |  |  ├─ MS1 Correction Factor:1
|    |  |  └─ MS2 Correction Factor: 1
|    |  └─ Main Search:       Dynamic
|    |     ├─ MS1 Correction Factor: 1
|    |     └─ MS2 Correction Factor:  1
|    ├─ Thermo Orbitrap:
|    |  ├─ Calibration Search:        Dynamic
|    |  |  ├─ MS1 Correction Factor:1
|    |  |  └─ MS2 Correction Factor: 1
|    |  └─ Main Search:       Dynamic
|    |     ├─ MS1 Correction Factor: 1
|    |     └─ MS2 Correction Factor:  1
|    └─ TOF:
|       ├─ Calibration Search:        Dynamic
|       |  ├─ MS1 Correction Factor: 1
|       |  └─ MS2 Correction Factor:  1
|       └─ Main Search:       Dynamic
|          ├─ MS1 Correction Factor:  1
|          └─ MS2 Correction Factor:   1
└─ Pulsar Search\Workflow
   ├─ Fragment Ion Selection Strategy:       Intensity Based
   ├─ In-Silico Generate Missing Channels:  False
   └─ Use DNN Predicted Ion Mobility:        Auto
[END-SETTINGS]
```

DIANN settings

DIA-NN 1.9.1 (Data-Independent Acquisition by Neural Networks)
Compiled on Jul 15 2024 15:40:36
Current date and time: Tue Dec 17 15:31:24 2024
CPU: GenuineIntel Intel(R) Core(TM) i9-10900 CPU @ 2.80GHz
SIMD instructions: AVX AVX2 FMA SSE4.1 SSE4.2
Logical CPU cores: 20

diann.exe --f D:\.... --lib --threads 20 --verbose 1 --out D:\...\report.tsv --qvalue 0.01 --matrices --out-lib D:\....parquet --gen-spec-lib --predictor --reannotate --xic --fasta camprotR_240512_cRAP_20190401_full_tags.fasta --cont-quant-exclude cRAP- --fasta Y:\Fasta\HUMAN_UP000005640_9606.fasta --fasta-search --min-fr-mz 200 --max-fr-mz 1800 --met-excision --min-pep-len 7 --max-pep-len 30 --min-pr-mz 300 --max-pr-mz 1800 --min-pr-charge 2 --max-pr-charge 4 --cut K*,R* --missed-cleavages 2 --unimod4 --var-mods 3 --var-mod UniMod:35,15.994915,M --var-mod UniMod:1,42.010565,*n --individual-mass-acc --individual-windows --peptidoforms --reanalyse --rt-profiling

Thread number set to 20
Output will be filtered at 0.01 FDR
Precursor/protein x samples expression level matrices will be saved along with the main report
A spectral library will be generated
Deep learning will be used to generate a new in silico spectral library from peptides provided
Library precursors will be reannotated using the FASTA database
XICs within 10 seconds from the apex will be extracted for each precursor and saved in .parquet format, a folder will be created next to the main report for the XICs storage
Peptides corresponding to protein sequence IDs tagged with cRAP- will be excluded from normalisation as well as quantification of protein groups that do not include proteins bearing the tag
DIA-NN will carry out FASTA digest for in silico lib generation
Min fragment m/z set to 200
Max fragment m/z set to 1800
N-terminal methionine excision enabled
Min peptide length set to 7
Max peptide length set to 30
Min precursor m/z set to 300
Max precursor m/z set to 1800
Min precursor charge set to 2
Max precursor charge set to 4
In silico digest will involve cuts at K*,R*
Maximum number of missed cleavages set to 2
Cysteine carbamidomethylation enabled as a fixed modification
Maximum number of variable modifications set to 3
Modification UniMod:35 with mass delta 15.9949 at M will be considered as variable
Modification UniMod:1 with mass delta 42.0106 at *n will be considered as variable
Mass accuracy will be determined separately for different runs
Scan windows will be inferred separately for different runs
Peptidoform scoring enabled
A spectral library will be created from the DIA runs and used to reanalyse them; .quant files will only be saved to disk during the first step
The spectral library (if generated) will retain the original spectra but will include empirically-aligned RTs
DIA-NN will optimise the mass accuracy separately for each run in the experiment. This is useful primarily for quick initial analyses, when it is not yet known which mass accuracy setting works best for a particular acquisition scheme.
The following variable modifications will be scored: UniMod:35 UniMod:1

ANNOTATED SPECTRA OF PEPTIDES FROM TABLES S1-S3

MAGE antigens		
MAGE	Sequence	Annotated Spectra
A3	EVDPIGHLY	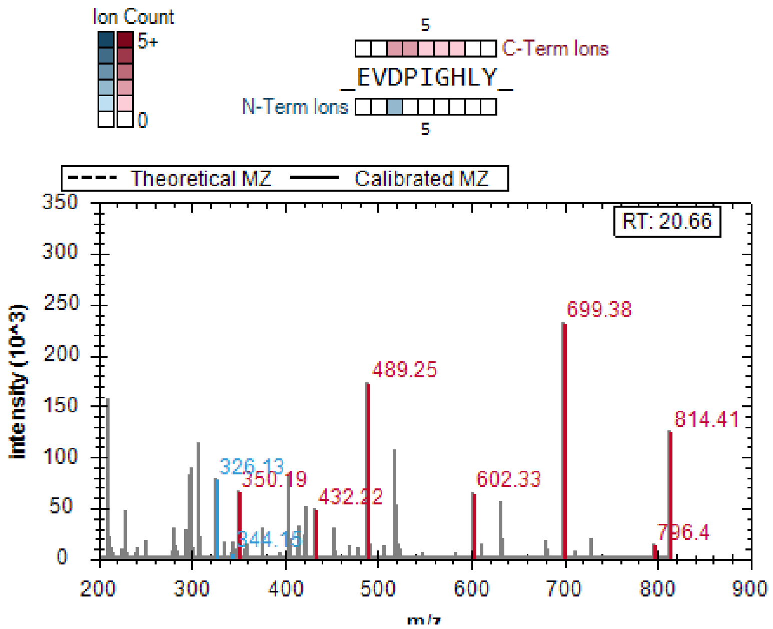
A4	AETSYVKVL	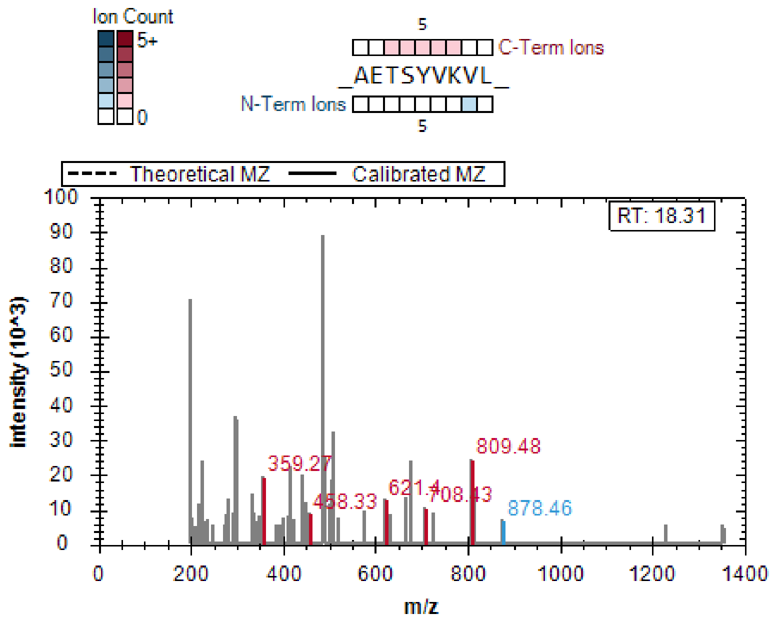

A4	EVDPASNTY	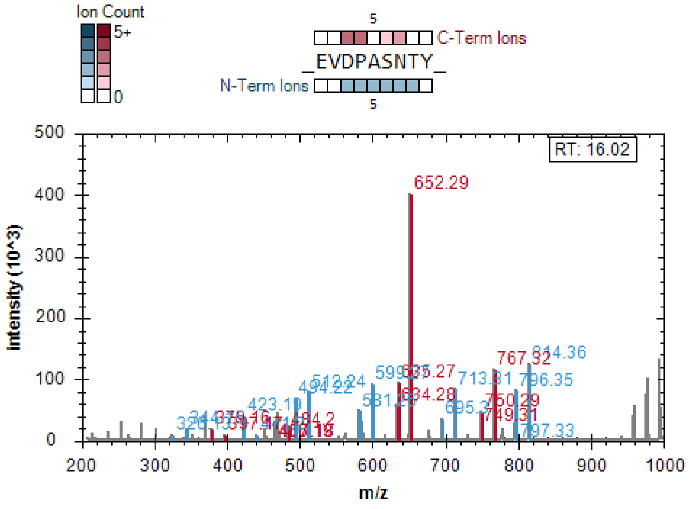
A4	GSNPARYEF	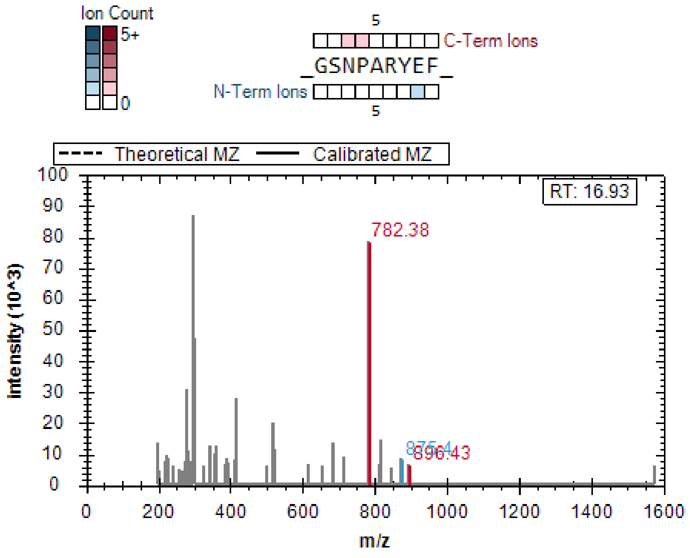
A4	KEVDPASNTY	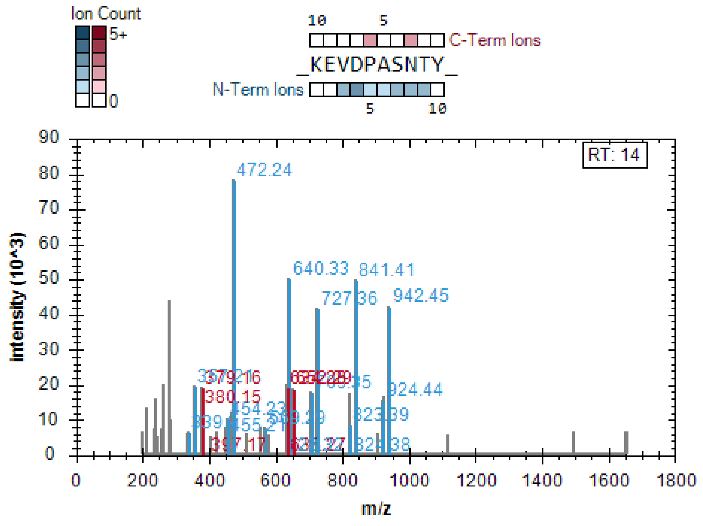

<table>
<tr><td>A4</td><td>TVYGEPRKL</td><td>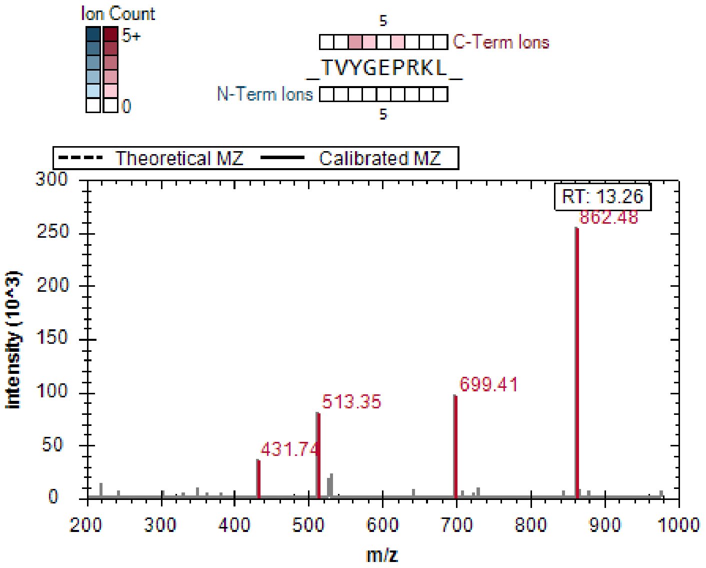
</td></tr>
<tr><td>A11</td><td>EVDPTSHSY</td><td>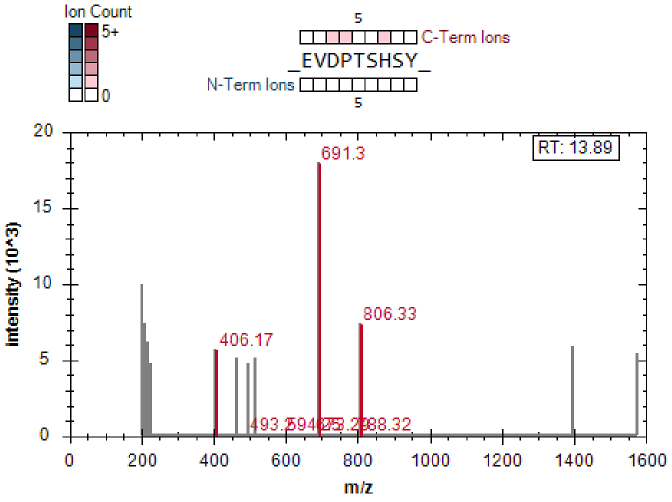
</td></tr>
<tr><td>C2</td><td>EEVPSGVIPNL</td><td>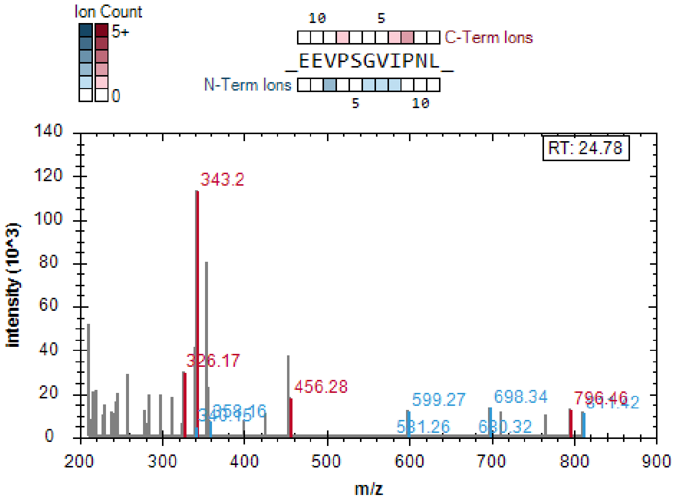
</td></tr>
</table>

C2	FVYGEPREL	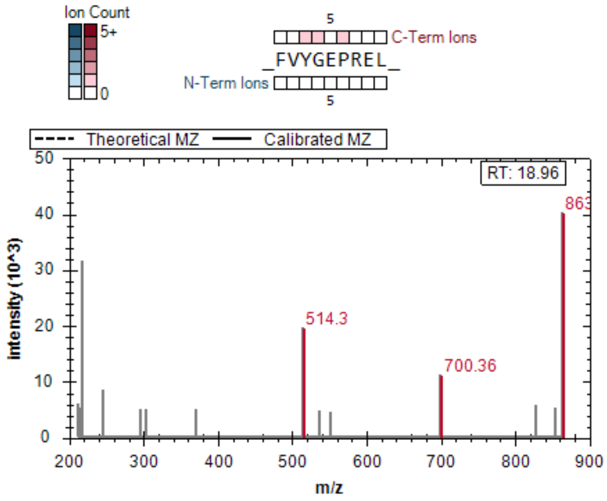

Epitopes from IEDB	
**Sequence**	**Annotated Spectra**
LLDVPTAAV	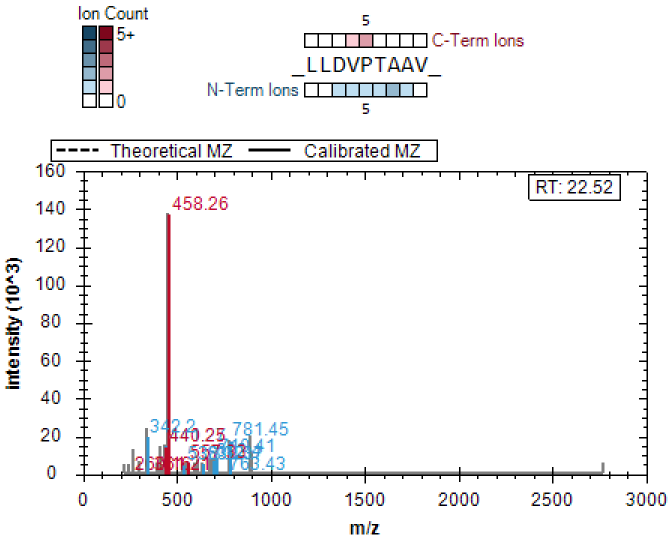
KLDVGNAEV	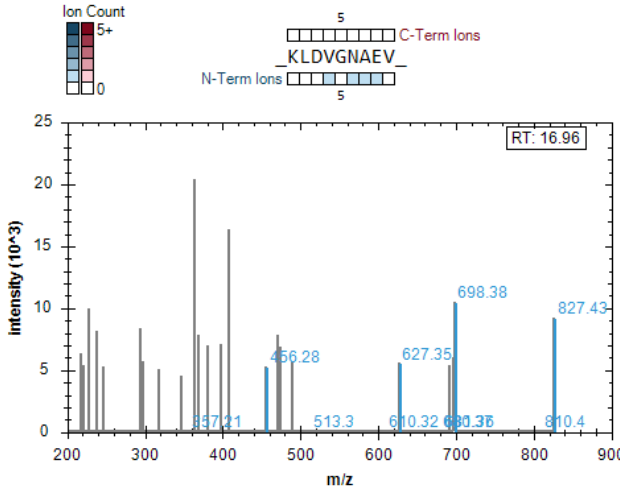
RLFDEPQLA	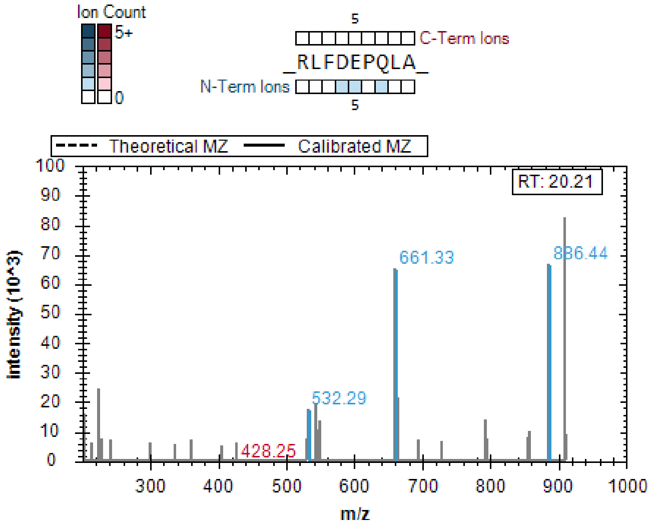

<table>
<tr><td>TLWVDPYEV</td><td>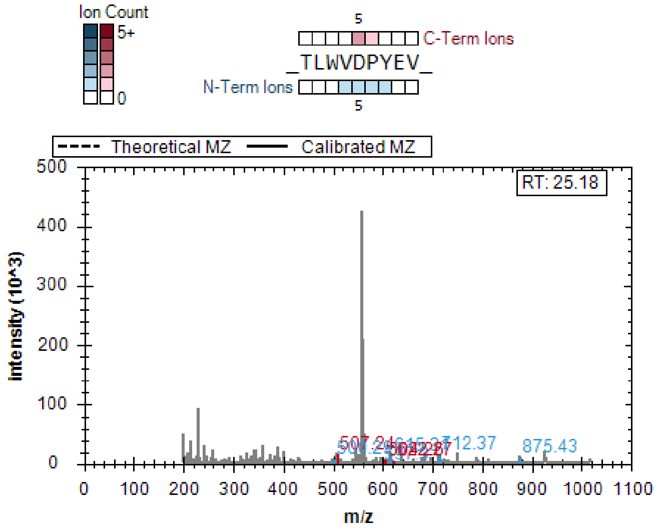
</td></tr>
<tr><td>TMLARLASA</td><td>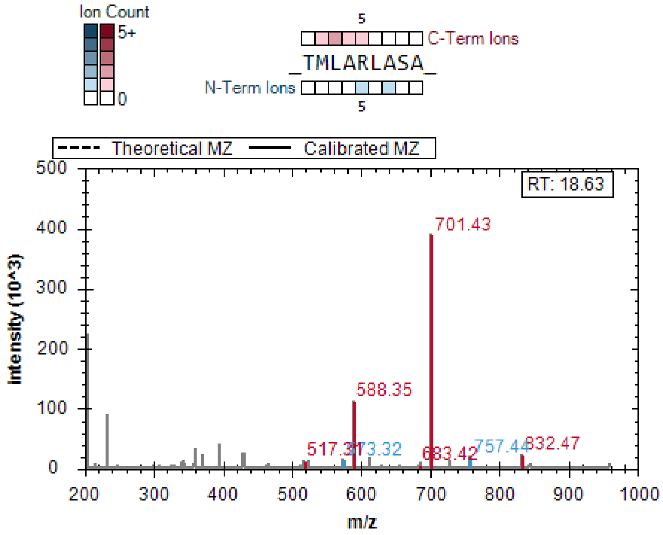
</td></tr>
<tr><td>GLIEKNIEL</td><td>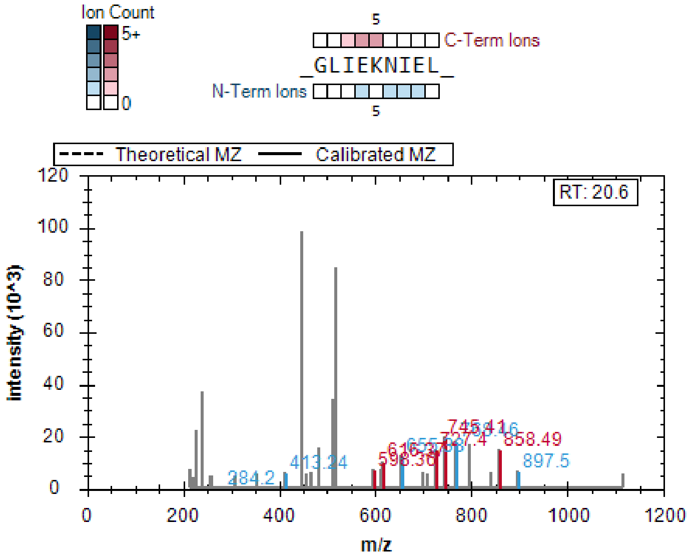
</td></tr>
</table>

<table>
<tr>
<td>LLDLDEELRY</td>
<td>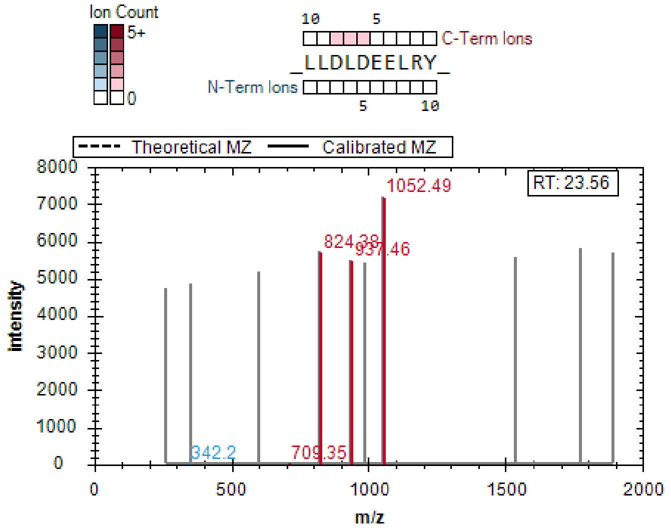
</td>
</tr>
<tr>
<td>EVDPIGHLY</td>
<td>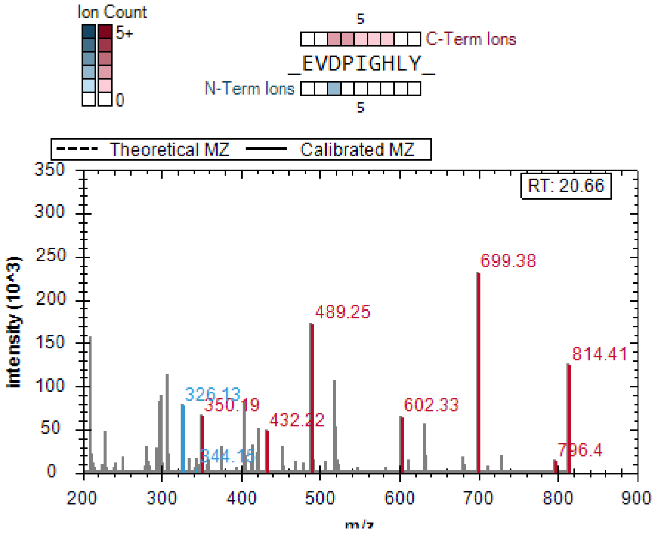
</td>
</tr>
<tr>
<td>FVYGEPREL</td>
<td>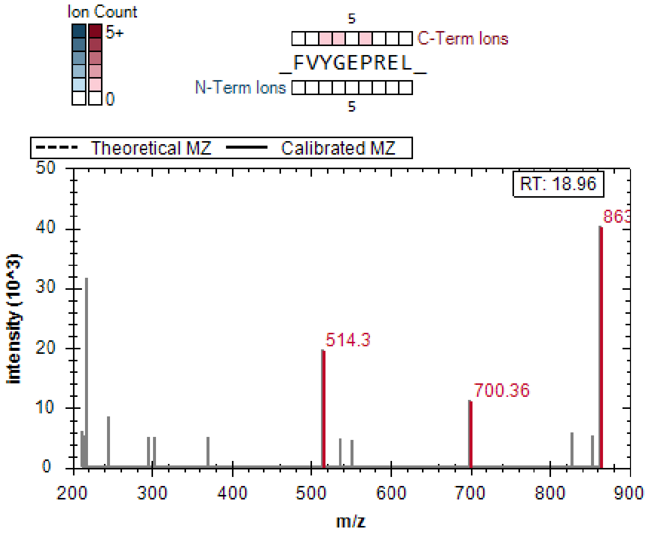
</td>
</tr>
</table>

SIIGRLLEV

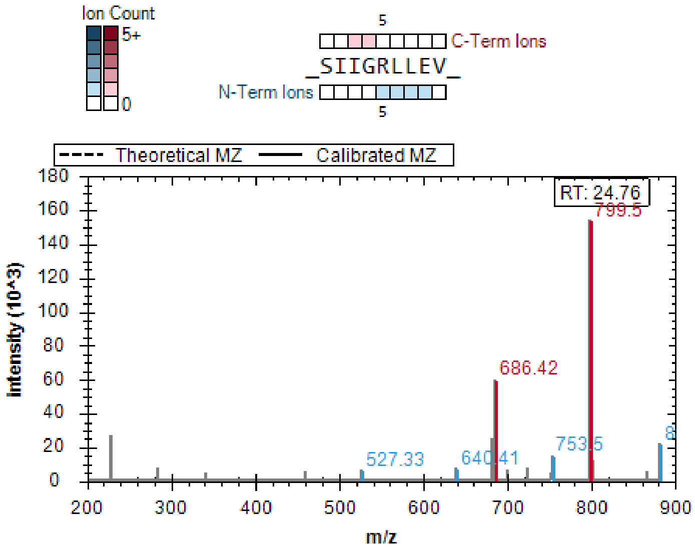

IMLEALERV

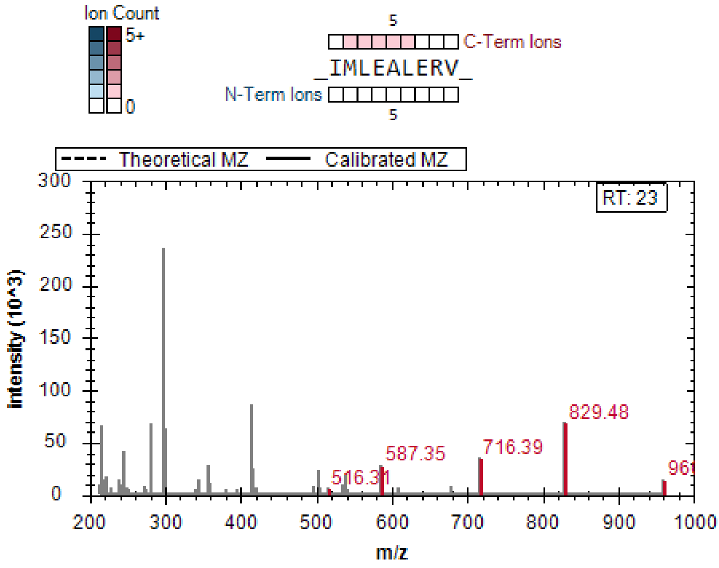

SEEEFLRTY

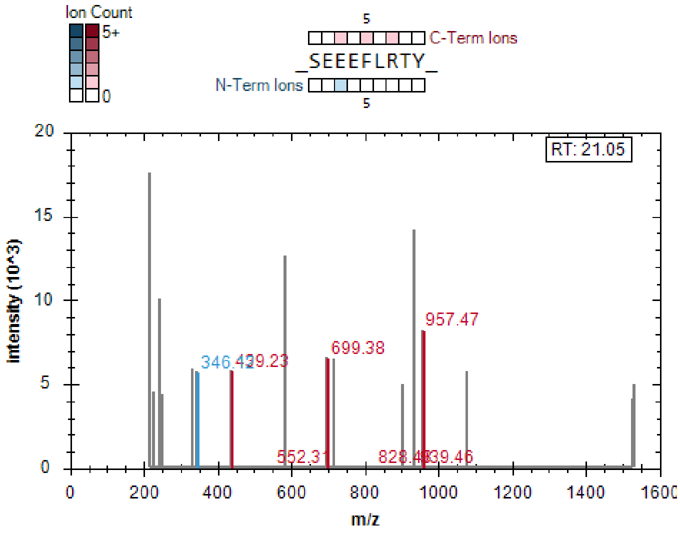

<table>
<tr><th colspan="2">Unannotated proteins</th></tr>
<tr><th>Sequence</th><th>Annotated Spectra</th></tr>
<tr><td>AEEPLAGRTW</td><td>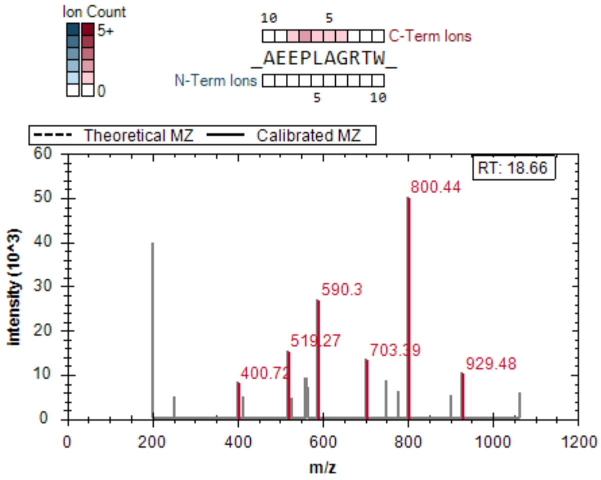
</td></tr>
<tr><td>ALAAVVTEV</td><td>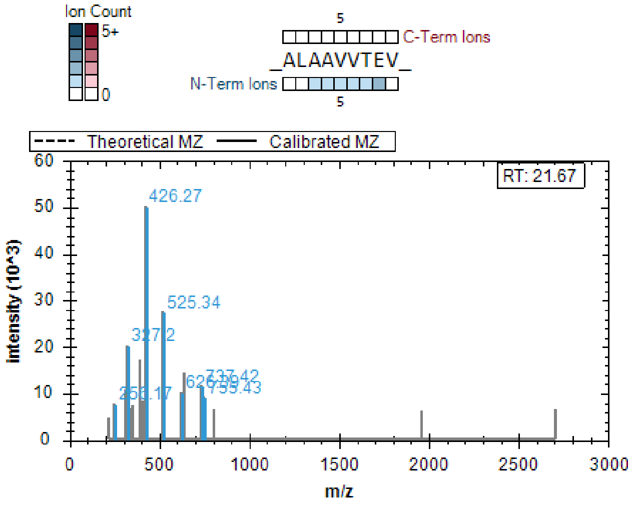
</td></tr>
<tr><td>EEAVVLRGL</td><td>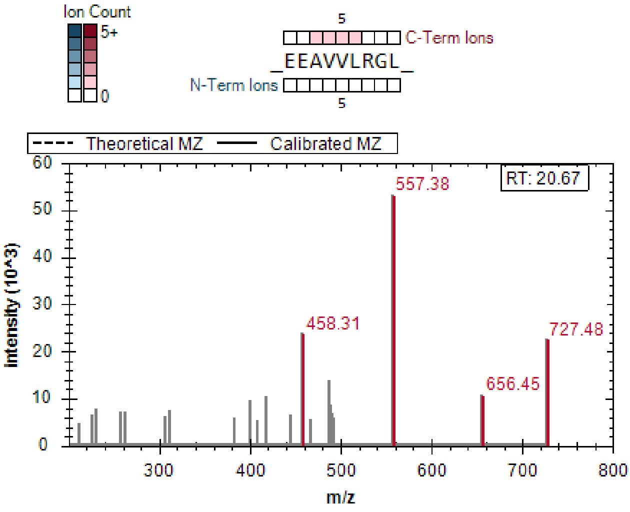
</td></tr>
</table>

<table>
<tr><td>EEYRGGNNW</td><td>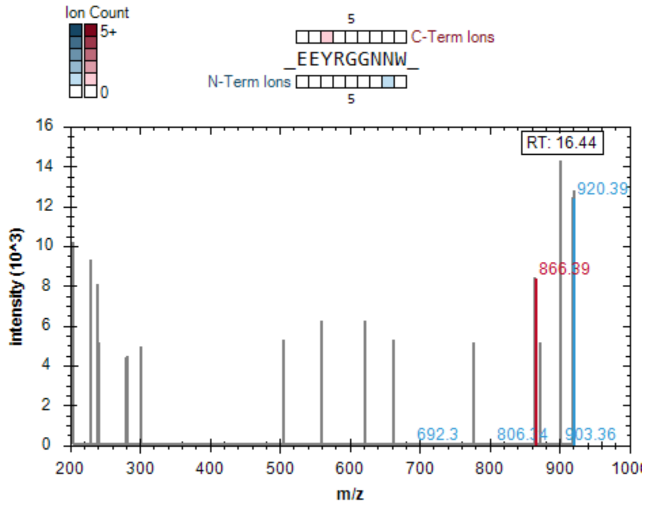
</td></tr>
<tr><td>FSNDALKTY</td><td>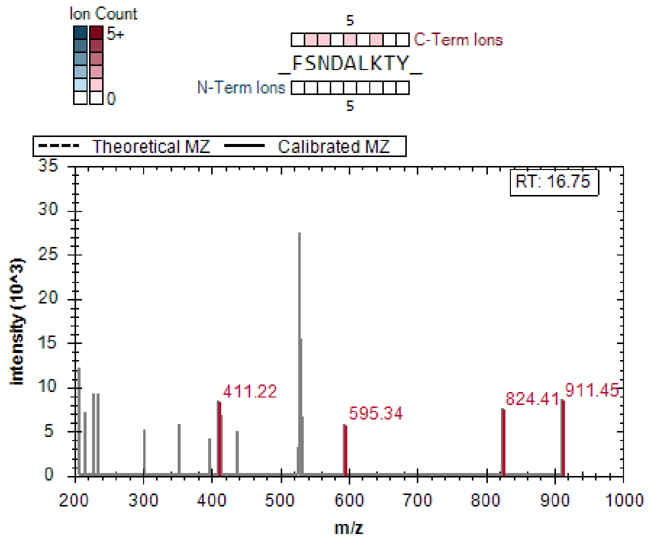
</td></tr>
<tr><td>IEVDGGRDW</td><td>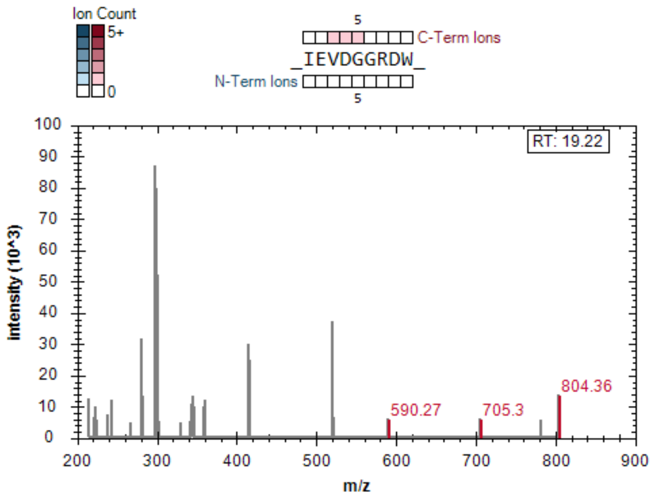
</td></tr>
</table>

IFSQRSYSY	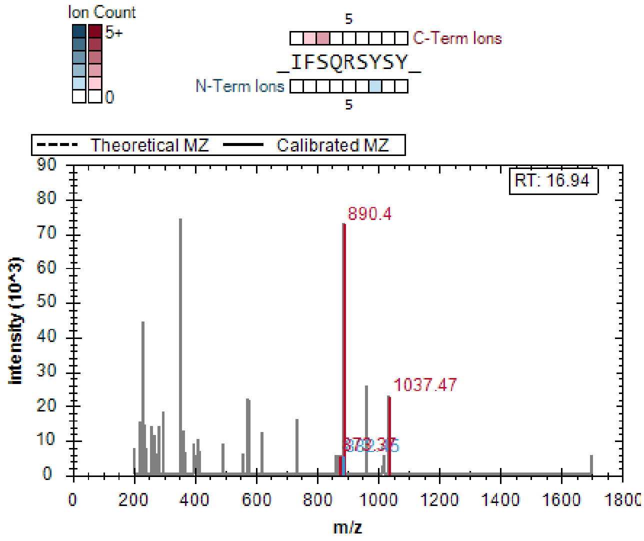
ISDPGVQGY	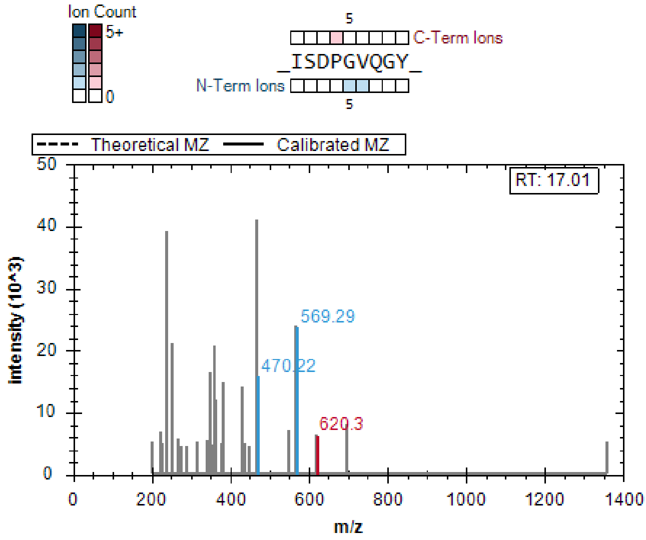
LVSNGVLVV	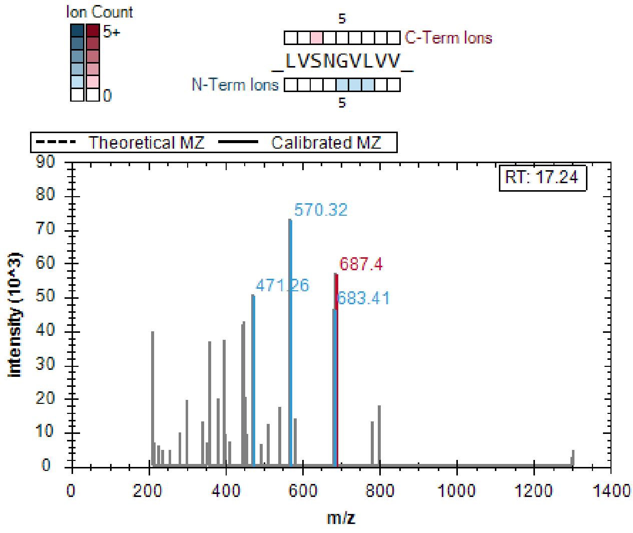

<table>
<tr><td>RTAAAAAGAASW</td><td>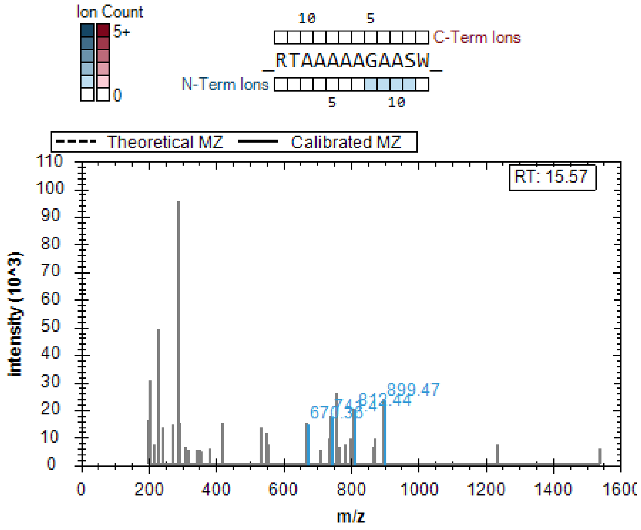
</td></tr>
<tr><td>RTDQFYVVY</td><td>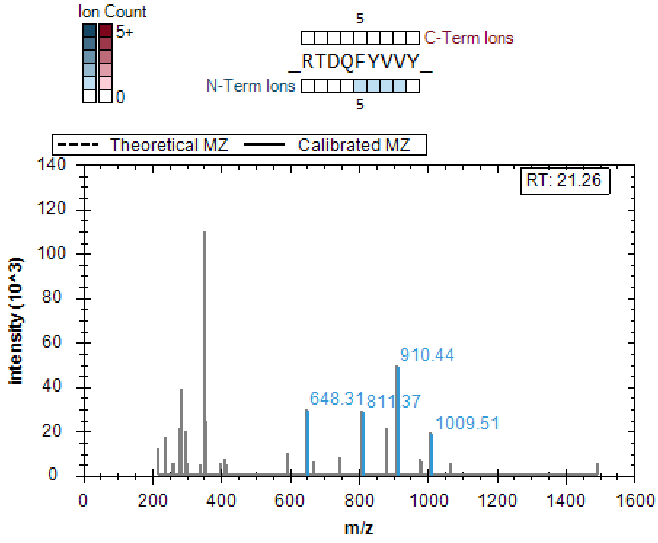
</td></tr>
<tr><td>SAAWDRPPL</td><td>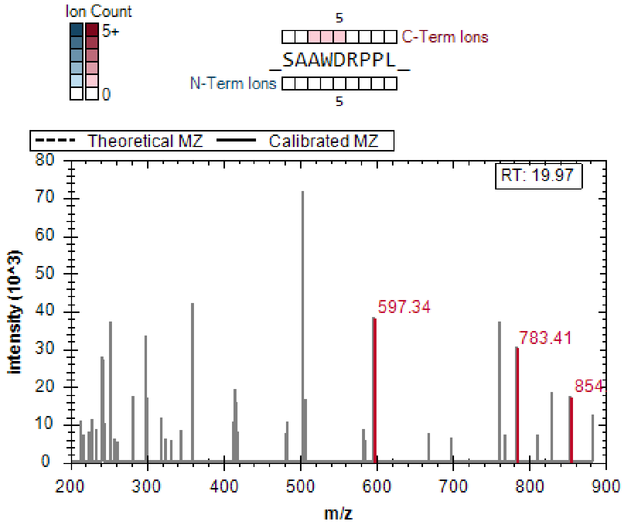
</td></tr>
</table>

<table>
<tr><td>STSTILRSF</td><td>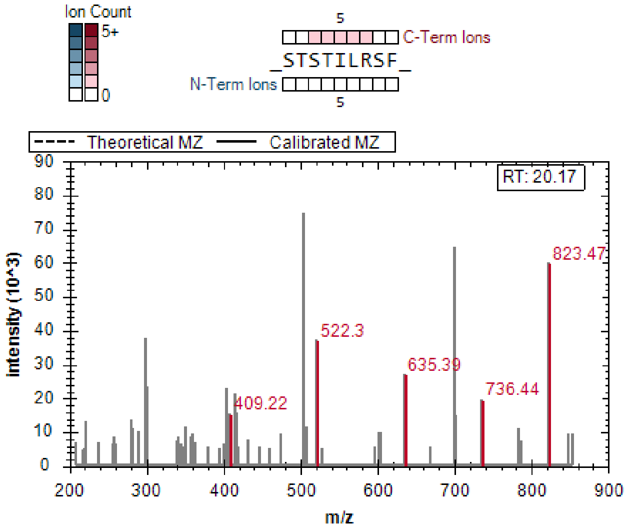
</td></tr>
<tr><td>TDGVSLLLP</td><td>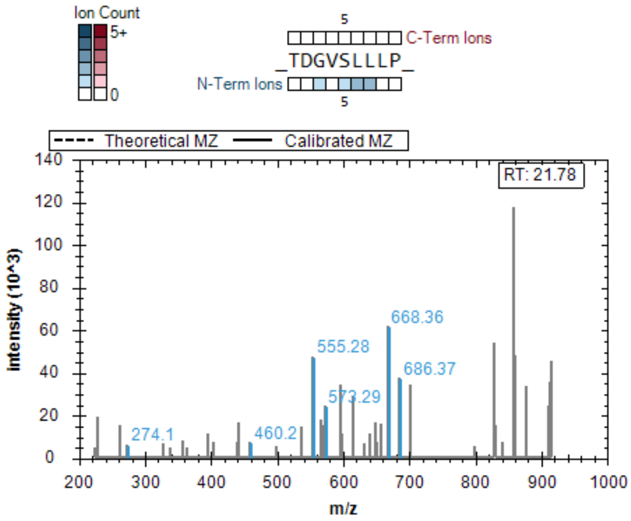
</td></tr>
<tr><td>TDNRTDIFY</td><td>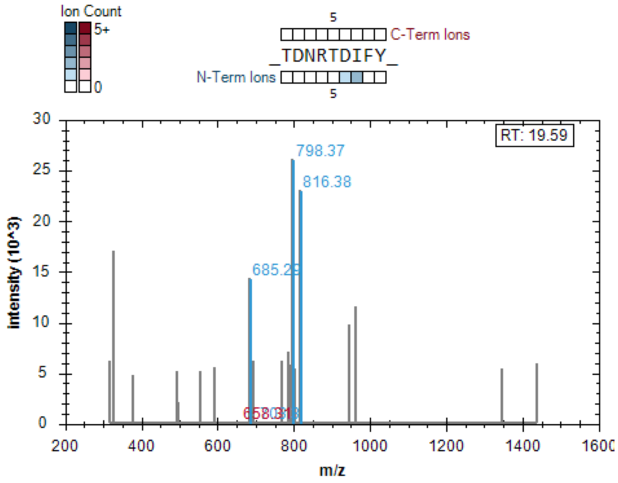
</td></tr>
</table>

VEDPIAEGGR	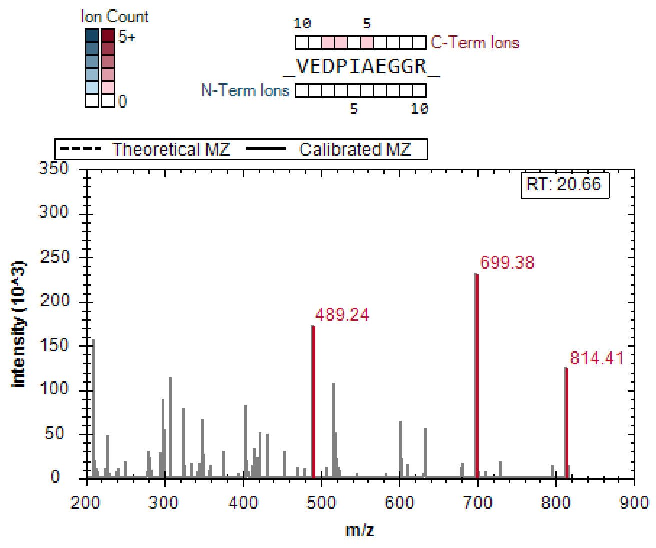
VTEKVYADTGLY	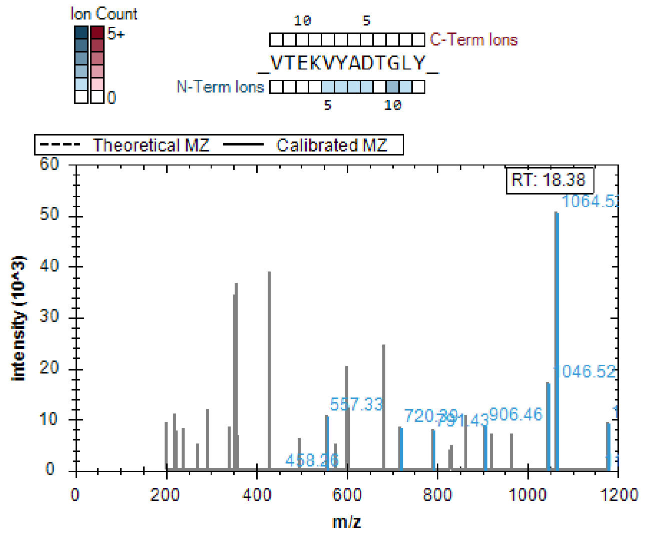

ANNOTATED SPECTRA OF DIFFERENTIALLY EXPRESSED PROTEINS

A375 CELLS

<table>
<tr><th>Protein</th><th>Peptide (Charge)</th><th>Spectra</th></tr>
<tr><td>ALG3</td><td>TGESILSLLR (2)</td><td>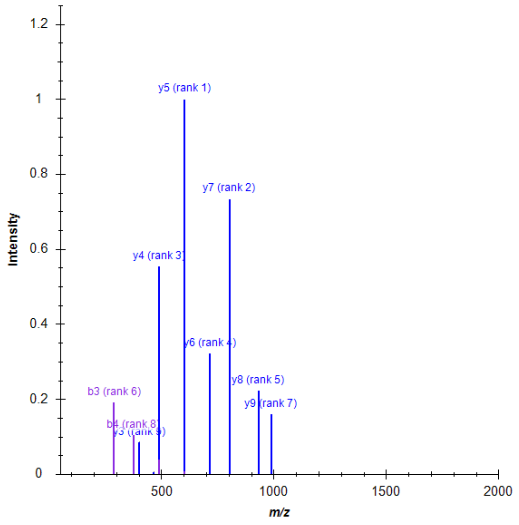
</td></tr>
<tr><td>BAP18</td><td>VGEIFSAAGAAFTK (2)</td><td>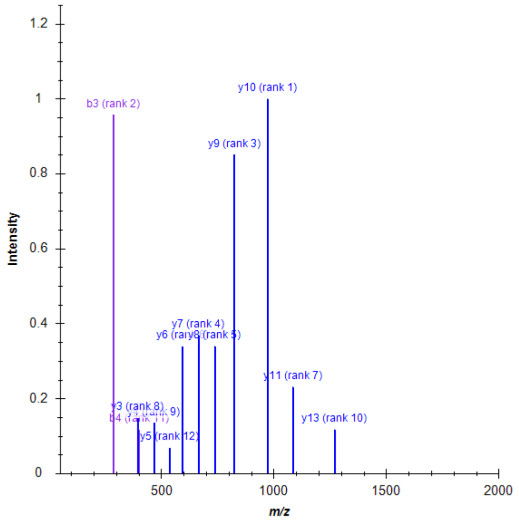
</td></tr>
</table>

CCDC85B	AVREDLGGCWQK (3)	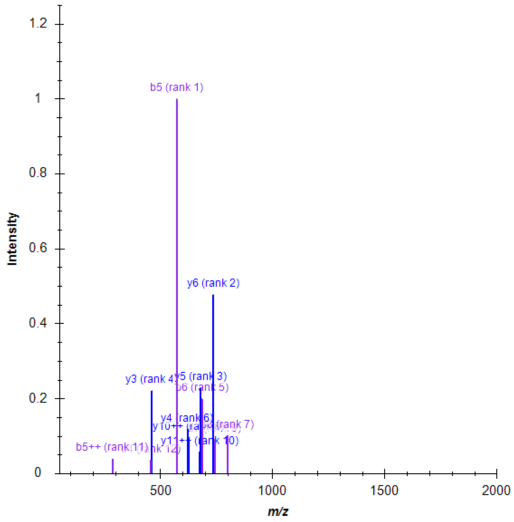
CDC42EP2	LRDLLSSDMISPPLGDFR (3)	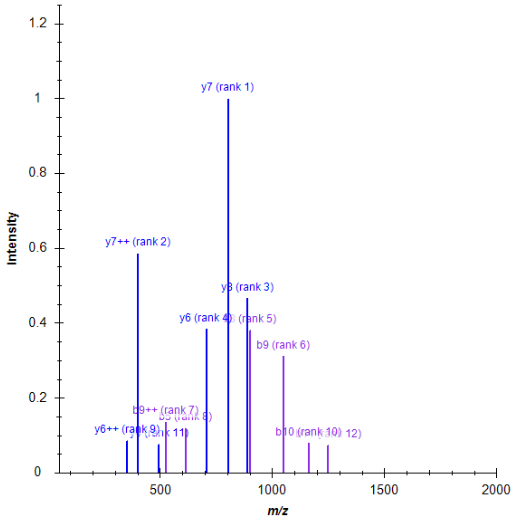
CLN3	LLAPLGLHLLPYSPR (3)	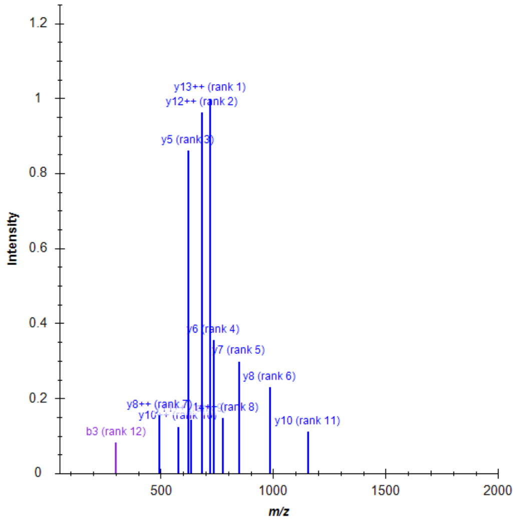

DCAF17 | LYSFQTIAEQFMQQK (3)

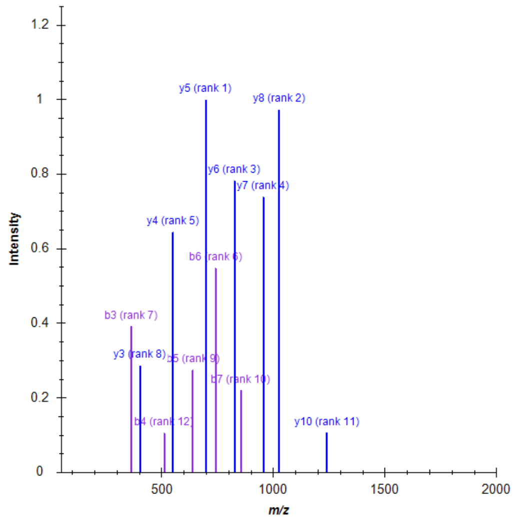

DCD | ENAGEDPGLAR (2)

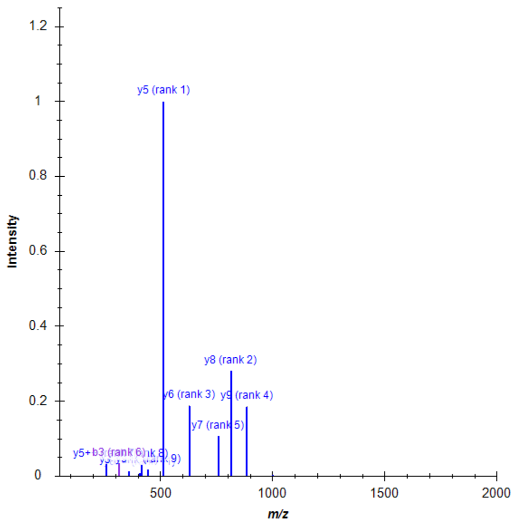

DUOXA2 | QAALPDLKCITTNL (2)

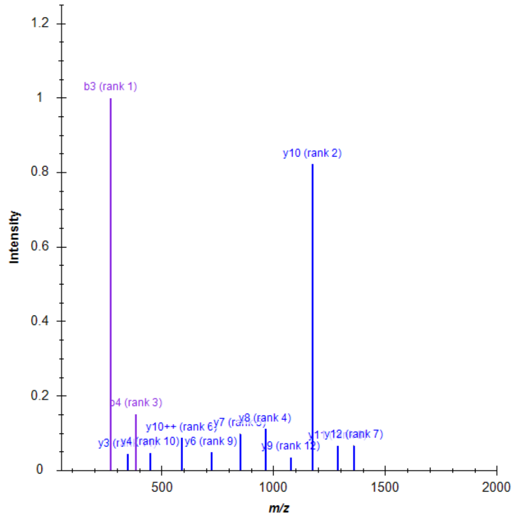

EVX1

MESRKDMVVFLDGGQLGT LVGK (4)

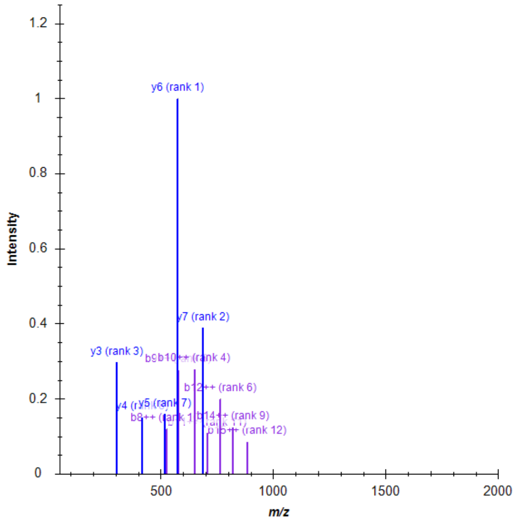

HLA-DQA1

FDPQFALTNIAVLK (2)

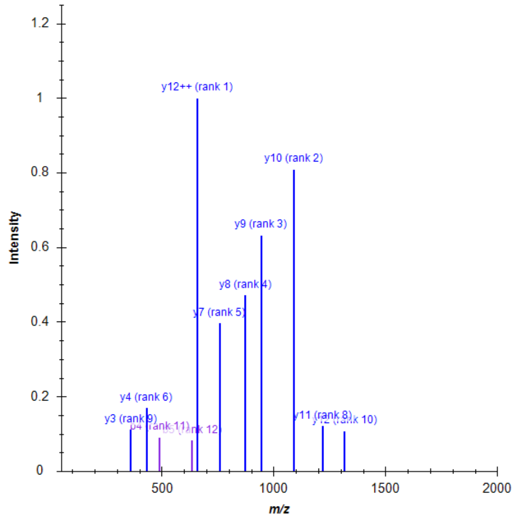

KAT8

RLDEWVDKNR (3)

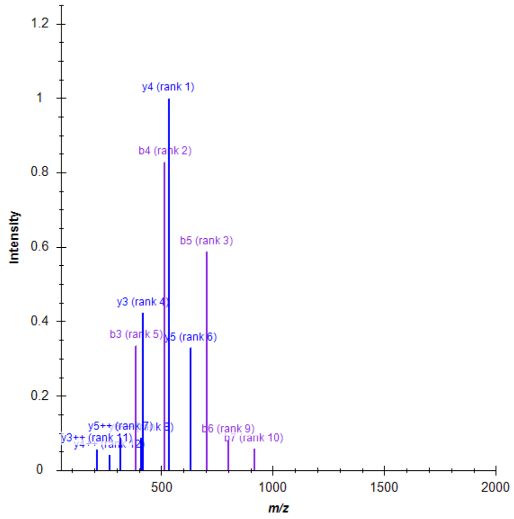

KDELR3	ALYLANWIR (2)	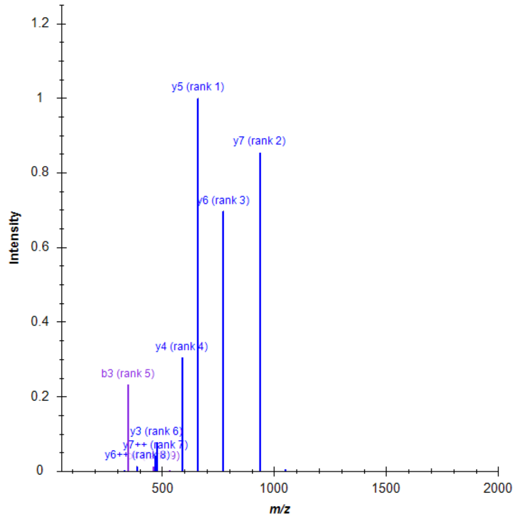
LEPROTL1	LVDDTDAMSNACK (2)	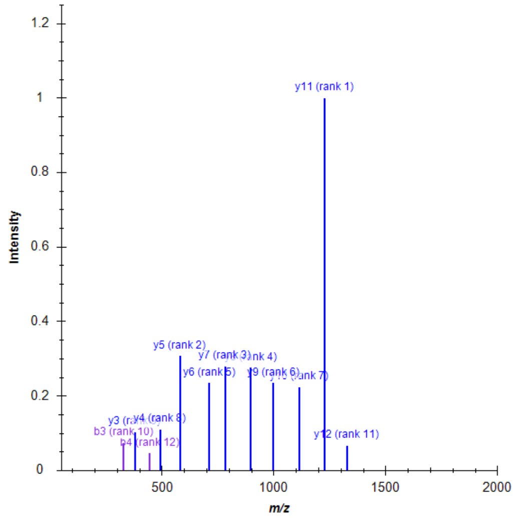
LILRA2	MGPVTSAHVGTYR (3)	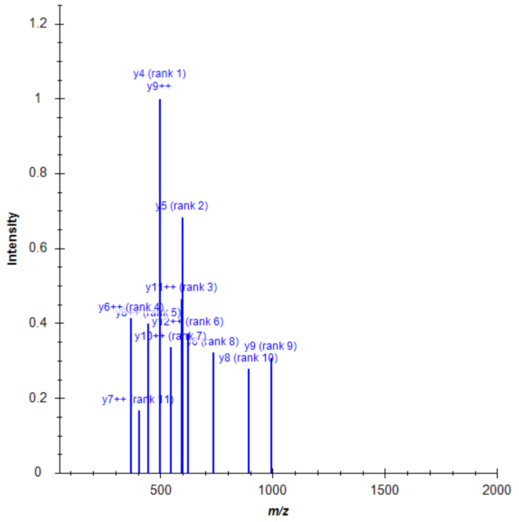

LOC122539214

EKETRMALPQGCLTFR (3)

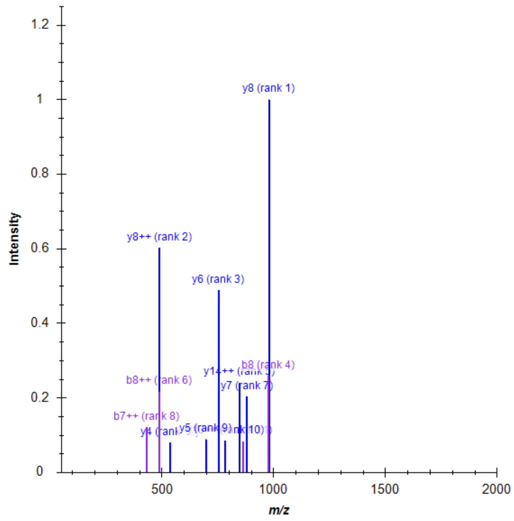

MAD2L2

NMEKIQVIKDFPWILADEQDVHMHDPR (4)

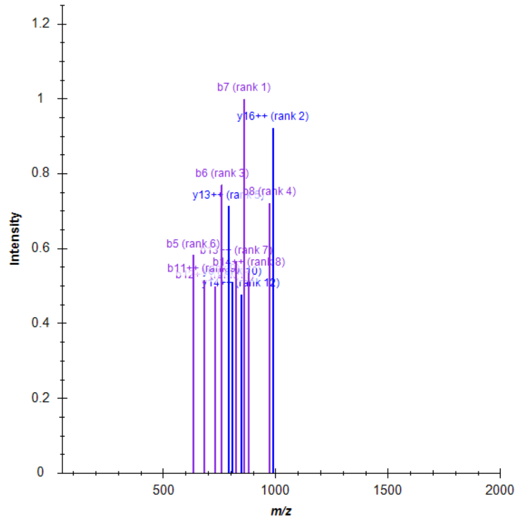

MAP1LC3A

IPVIIER (2)

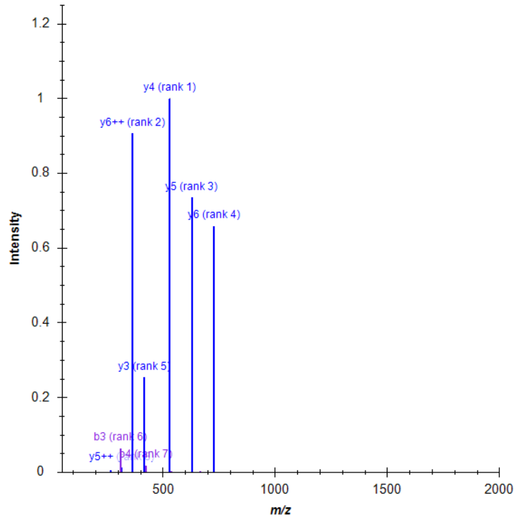

MEP1A	AMLEEALPVSLSQGQPSR QK (3)	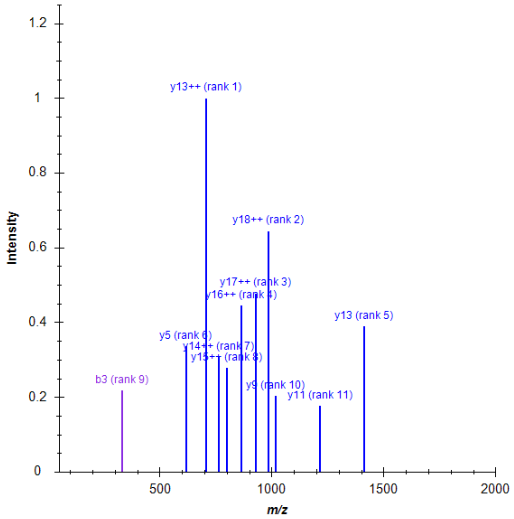
MGST2	VTPPAVTGSPEFER (2)	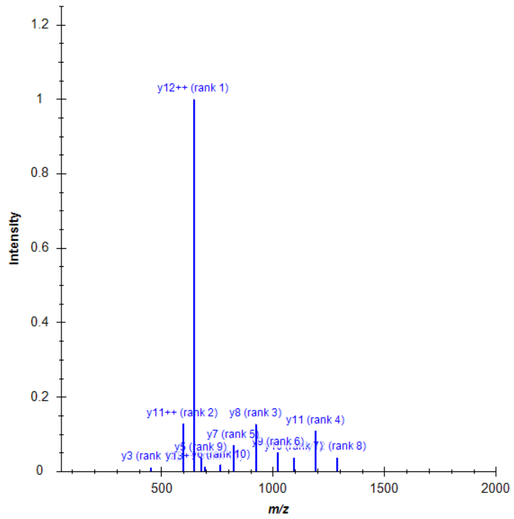
MT-ATP6	QMMTMHNTK (2)	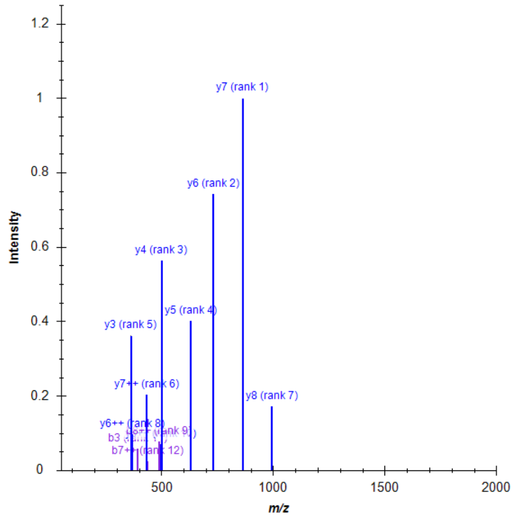

NFYB	ESFREQDIYLPIANVAR (3)	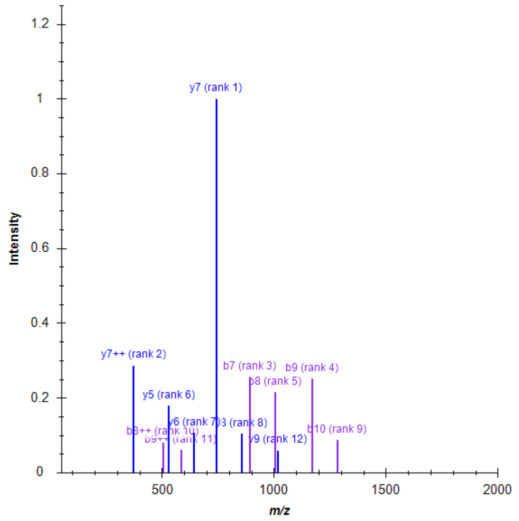
PEMT	SHCFTQAMLSQPR (3)	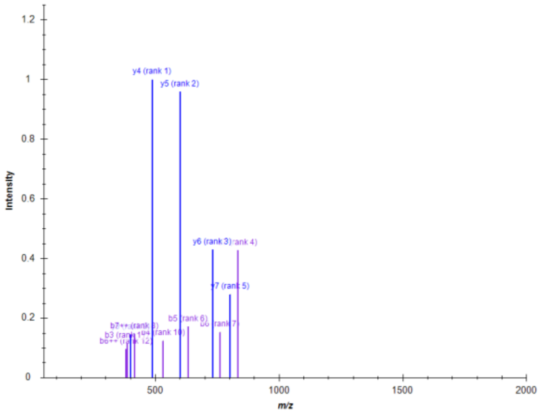
PFN3	GVHGGILNK (2)	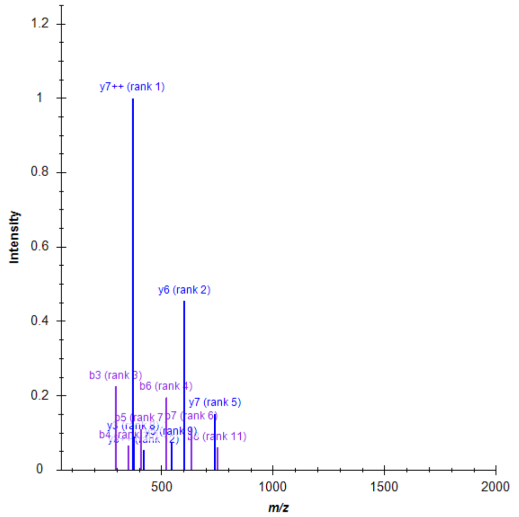

PSG11 MGPLSAPPCTEHIK (3)

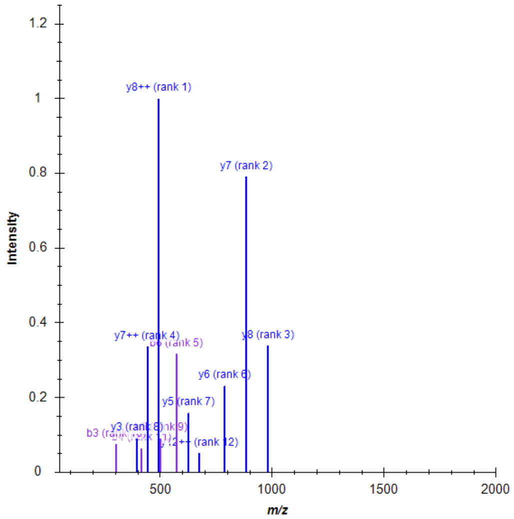

QPRT GPAHCLLLGER (3)

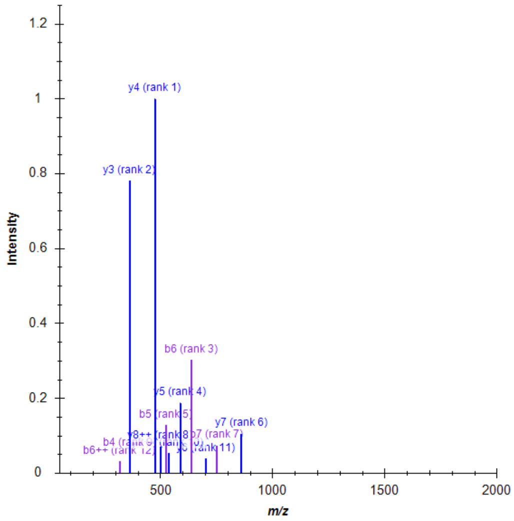

RABAC1 ATIRPWSTFVDQQR (3)

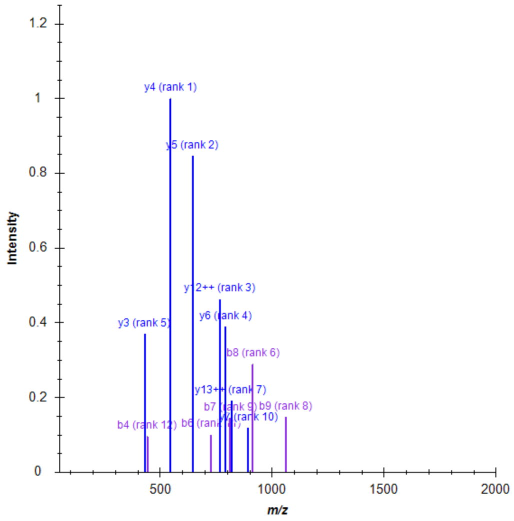

RAP1B	MREYKLVVLGSGGVGK (3)	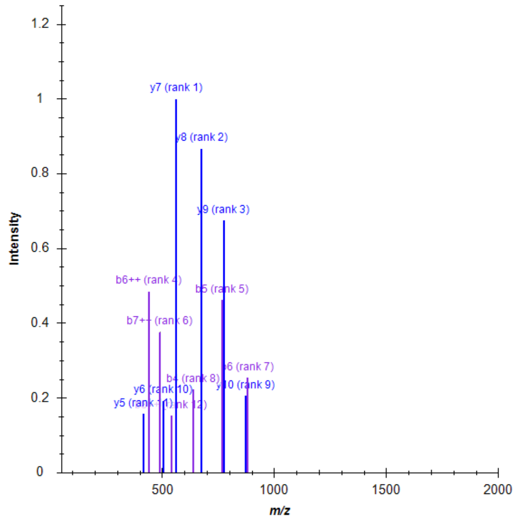
RMND5B	LKCPYCPMEQNPADGK (4)	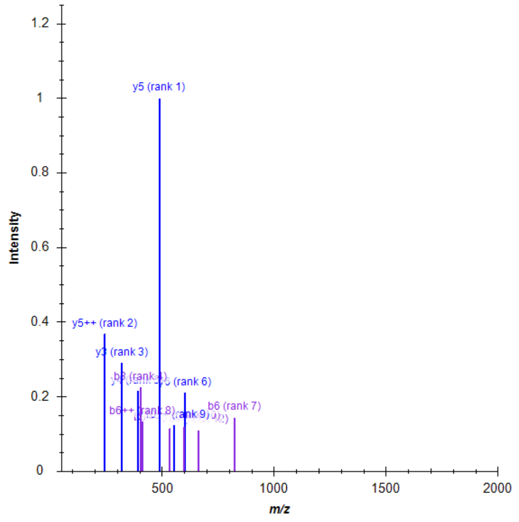
RNF139	MAAVGPPQQQVR (3)	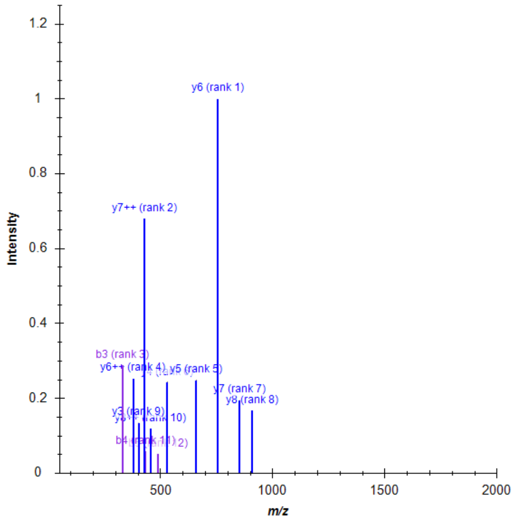

SLC16A7	VSNAQSVTSERETNI (2)	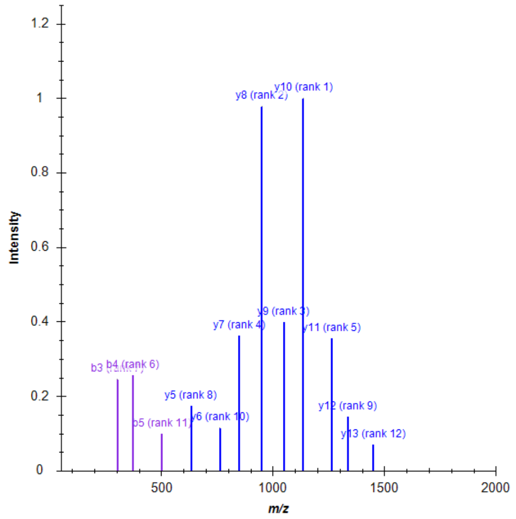
SLC25A30	EIRYRGMLHALVR (4)	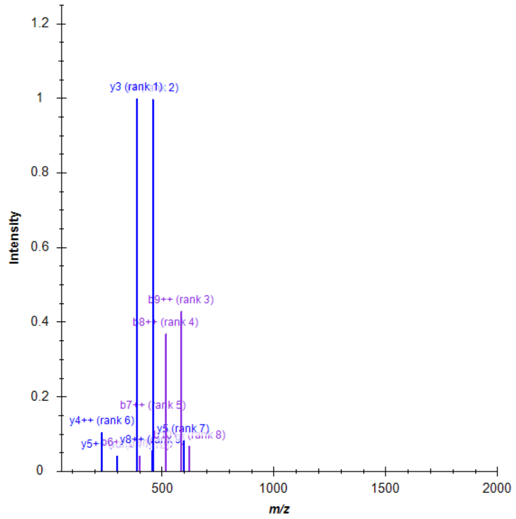
TMEM19	RLDSEYKEGGQR (3)	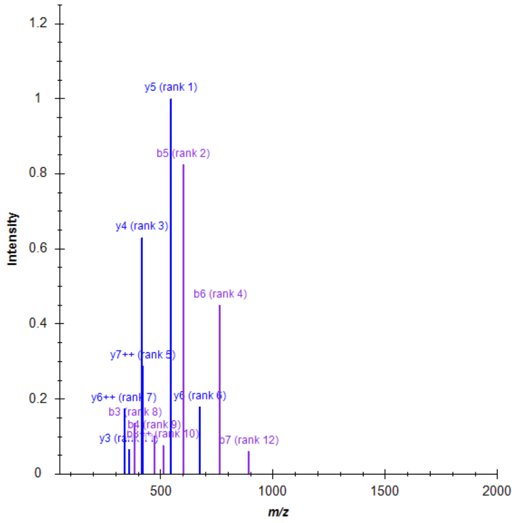

TMEM123

TIDEHAII (2)

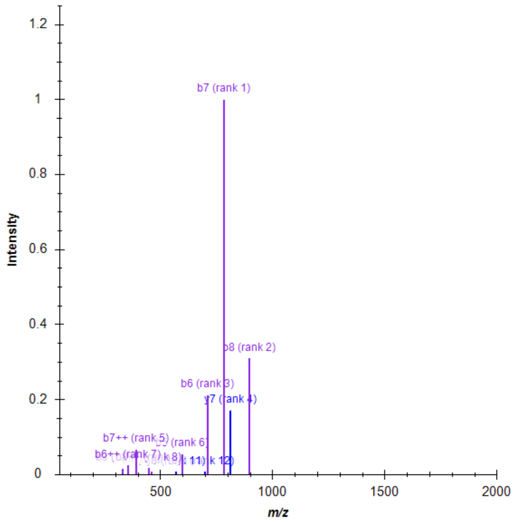

YIPF5

QYAGYDYSQQGR (2)

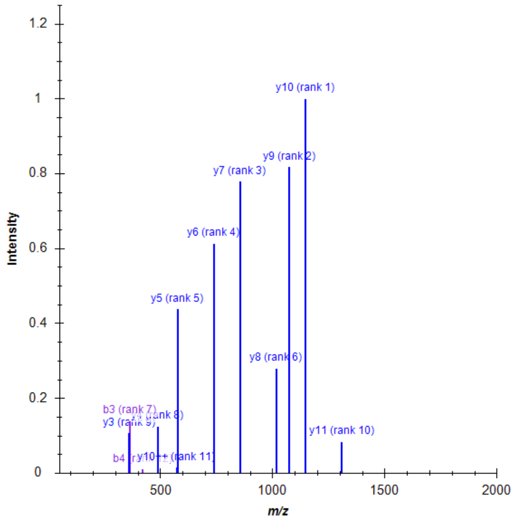

THP-1 CELLS

Protein	Peptide (Charge)	Spectra
ABHD11	ILAQQTGR (2)	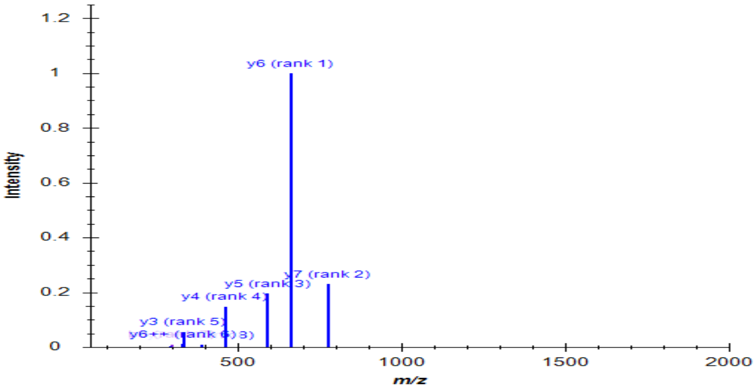
ACTR6	EEMVLSGKYK (3)	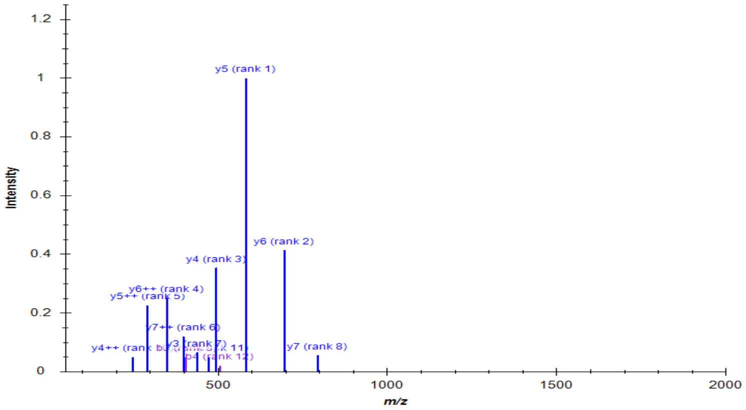
AKR1D1	SLGVSNFNR (2)	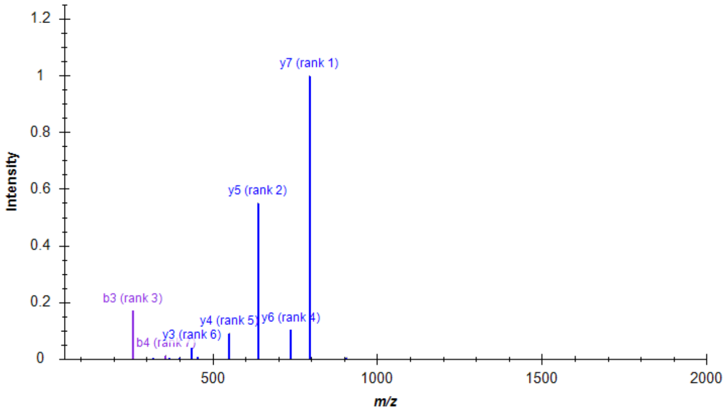

ALOX5AP	TGTLAFER (2)	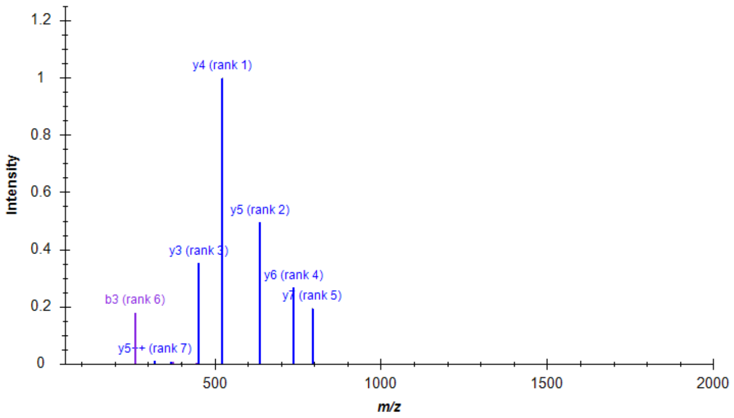
AREL1	VNFFQRELRQVHMK (4)	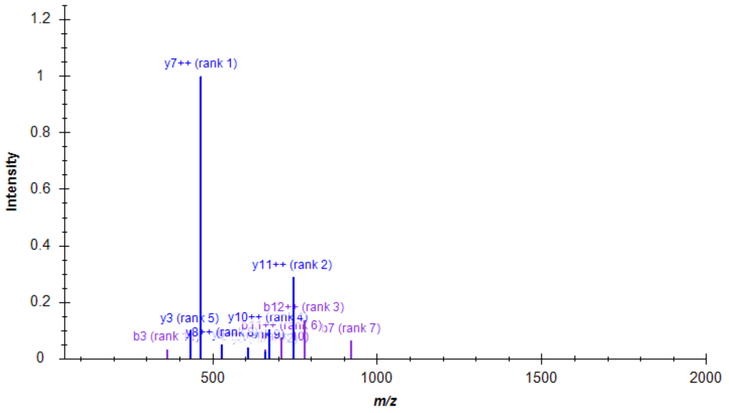
BCL10	EDTEEISCR (2)	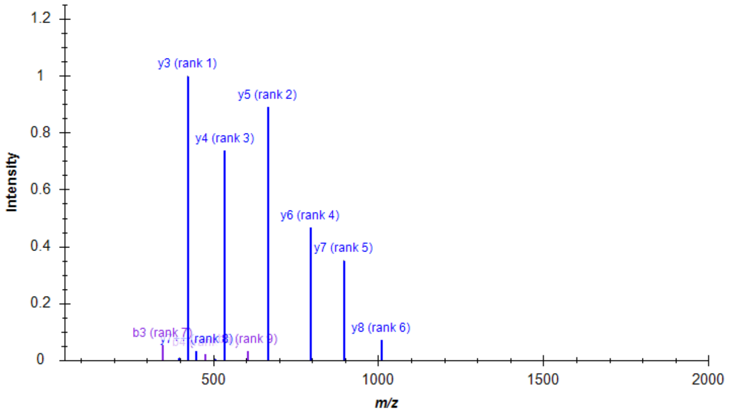

BRPF3	AMIHLSR (2)	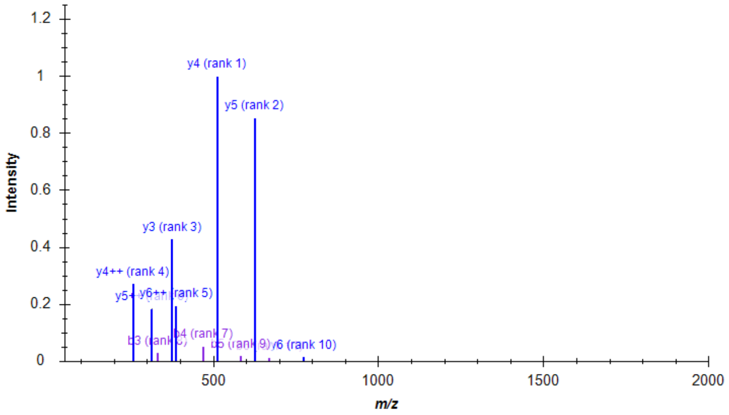
C8orf48	LKDSNFER (2)	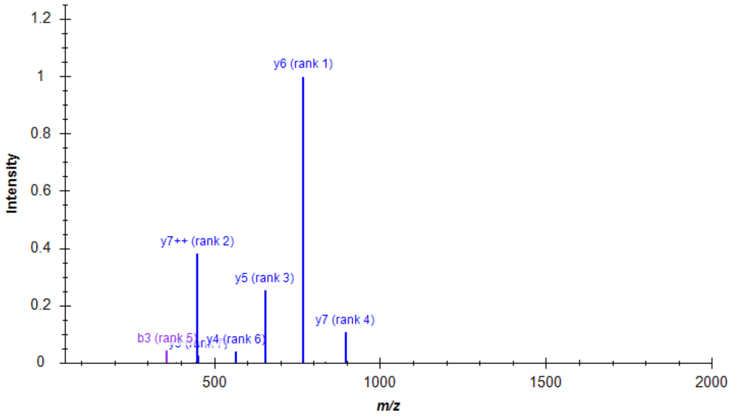
CCDC24	AEVAMLR (2)	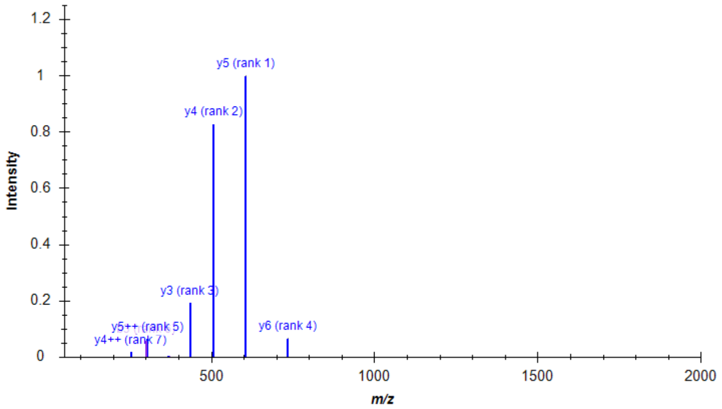

CD93	GGATCVLGPHGK (2)	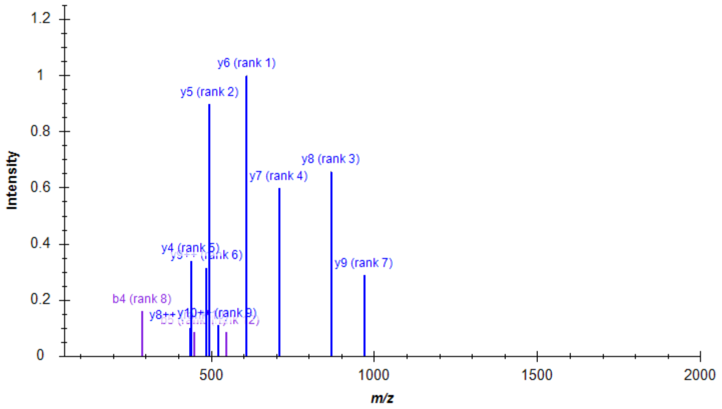
CDH16	CNVEGQCMR (3)	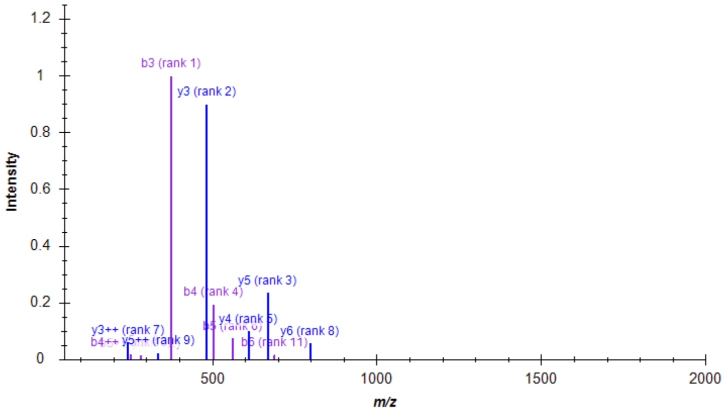
CDR2L	QLCEMEACR (3)	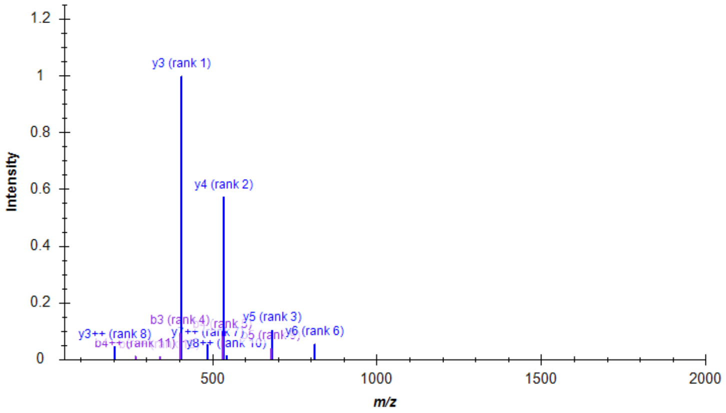

CEBPB

VLELTAENER (2)

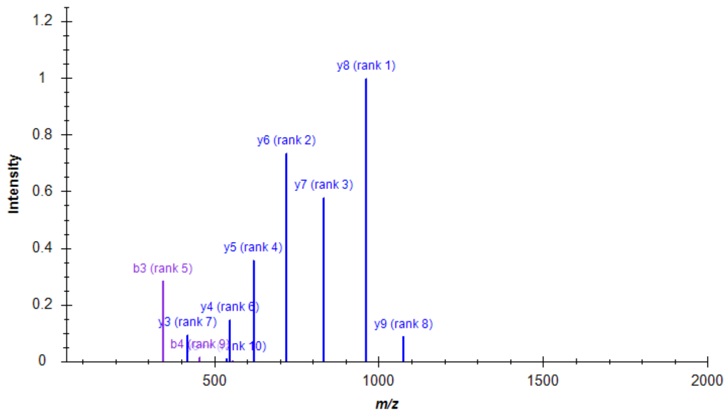

CIAO2B

SGERPVTAGEEDEQVPDS IDAR (3)

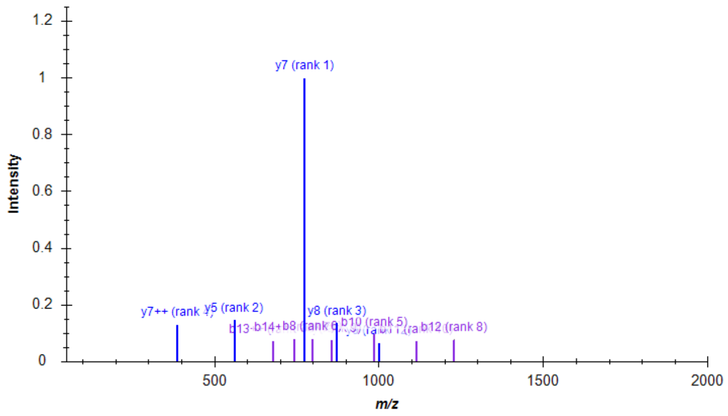

CMKLR1

MSSMNER (2)

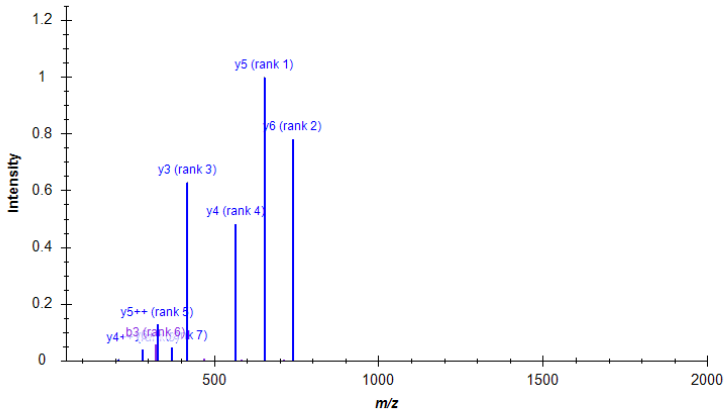

CNEP1R1	VVAPSIIAAR (2)	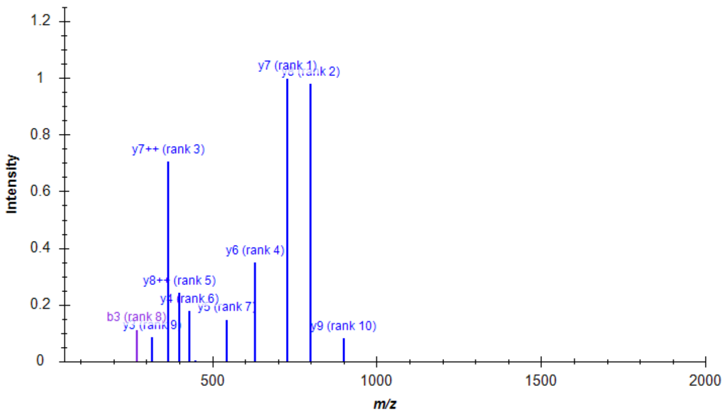
COA1	SEGLLYVHSSR (3)	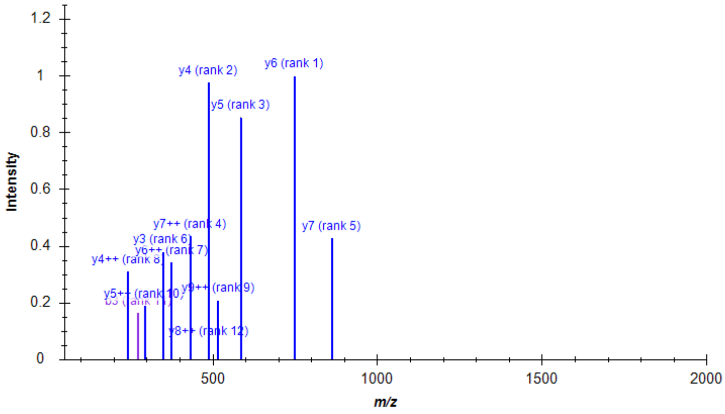
COL23A1	VAALEEER (2)	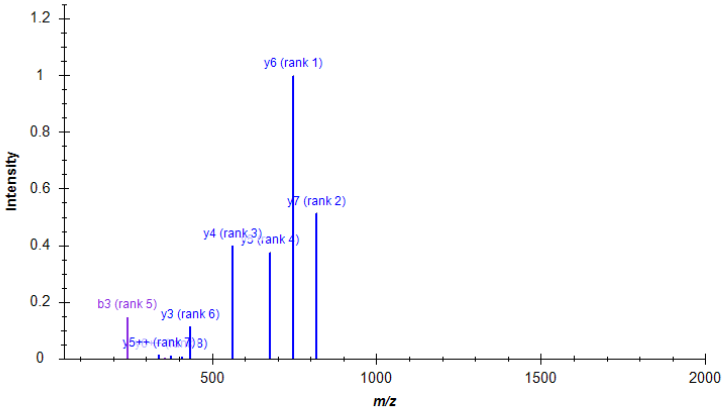

COPZ1

YYDDTYPSVK (2)

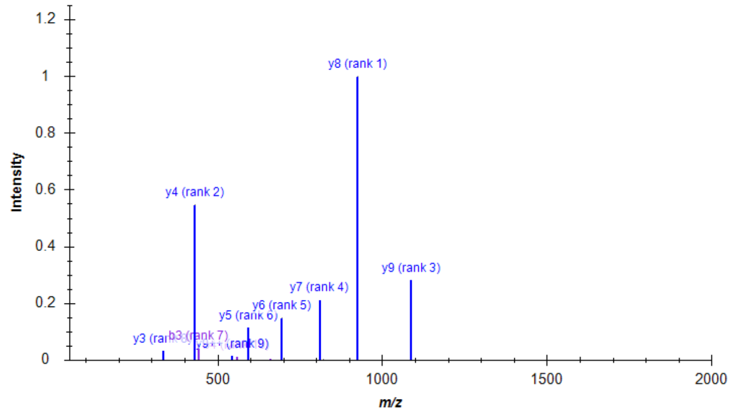

CSAD

MVPEDLER (2)

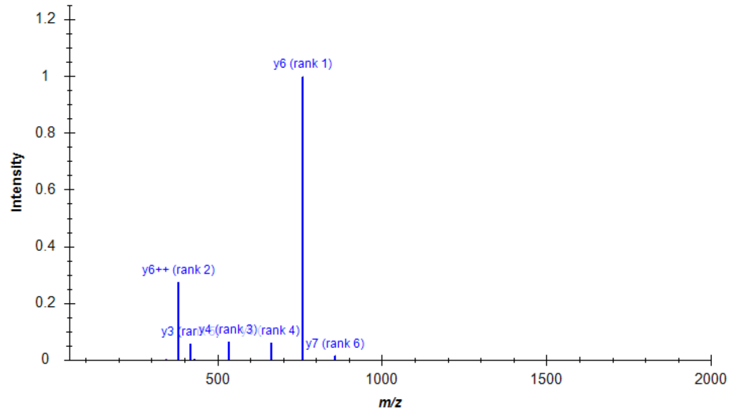

CX3CR1

SVHVDFSSSESQR (2)

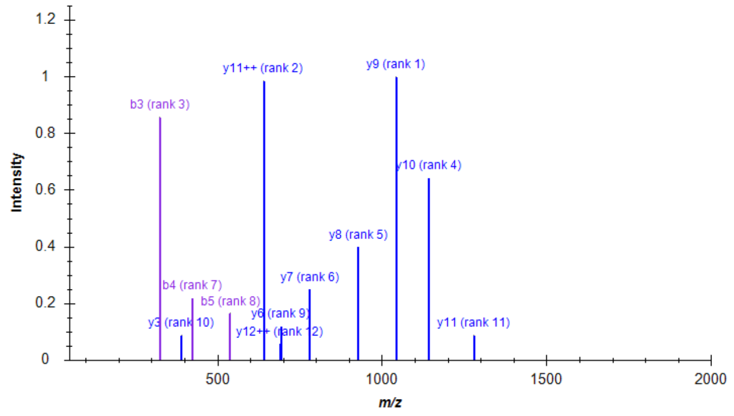

CXCR2 — TQVIQETCER (2)

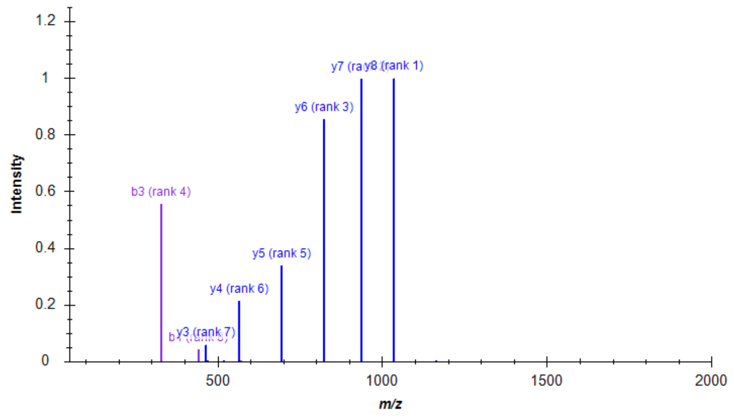

DNMBP — FCESNIESLNMELQQLR (4)

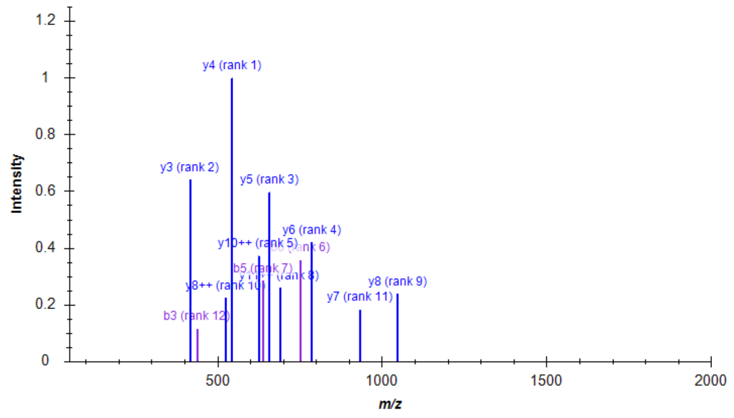

EVPLL — MQASADQVER (3)

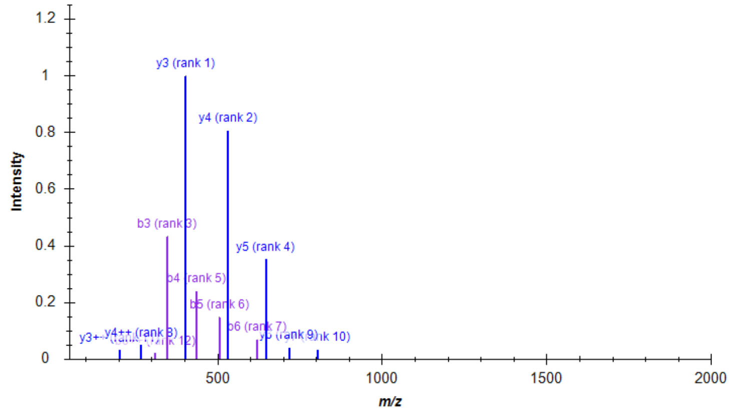

FAM117B	TSPTVATQTGASATSTR (2)	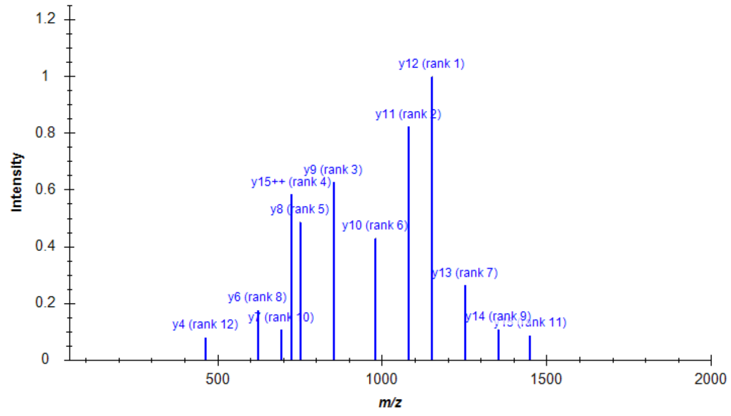
FBXO47	YLSVKDISMLSMVSK (4)	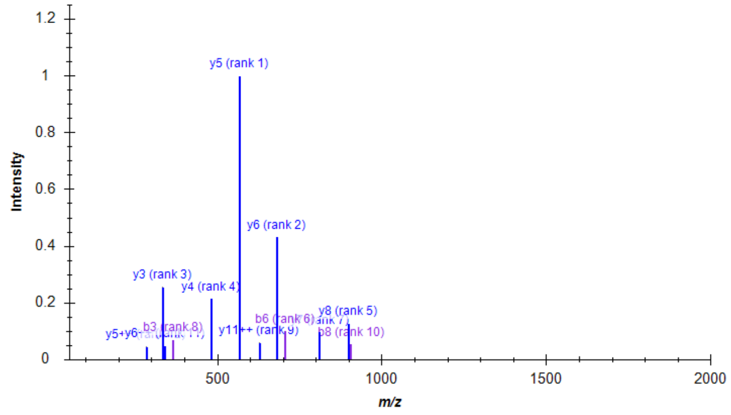
FBXO7	LGENVANIYK (2)	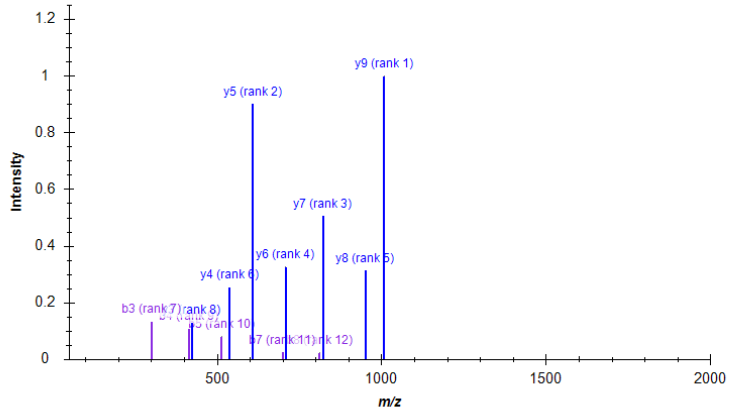

FTSJ1 EIRPQDCPISR (3)

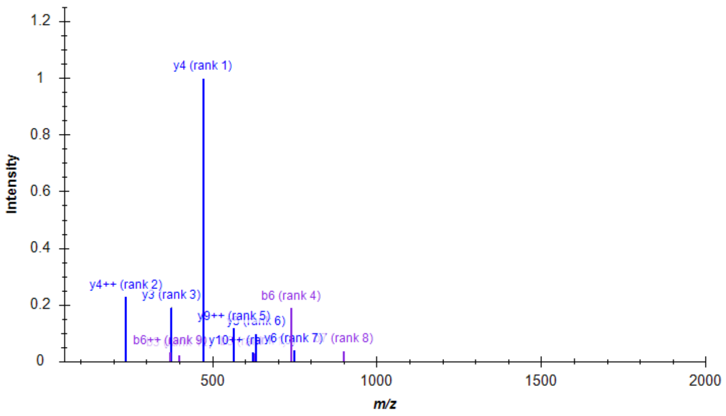

GALK2 HVYSEAAR (2)

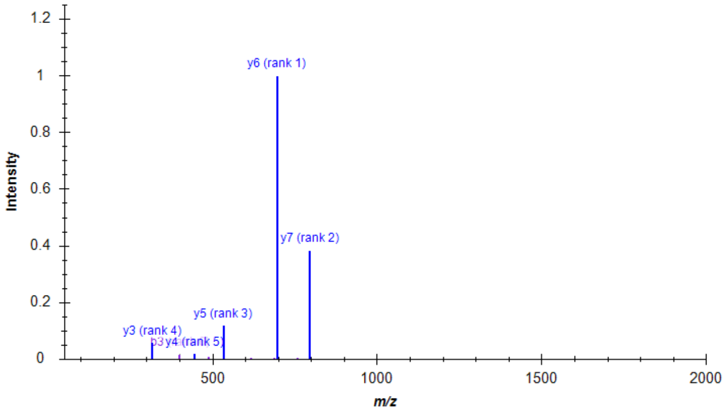

GAPT HEISVETQDHK (3)

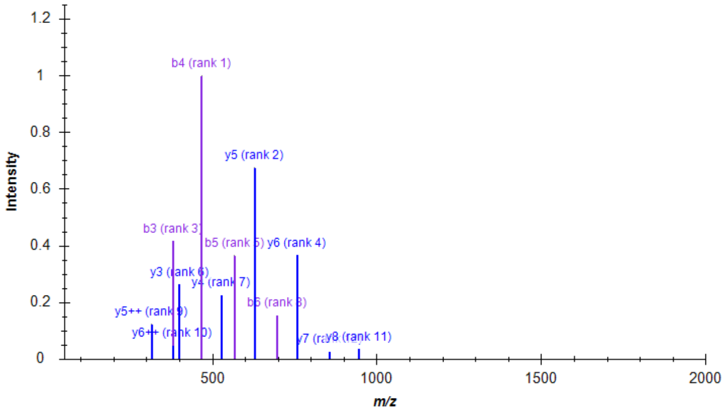

GINS2 | VSADSDVR (2)

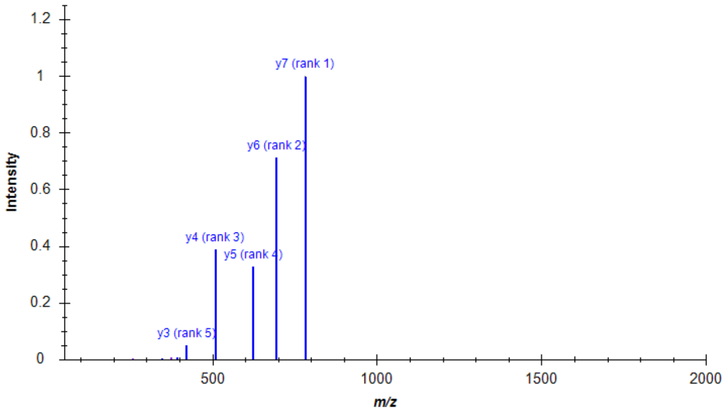

GNPDA2 | IIQFKPGQDR (3)

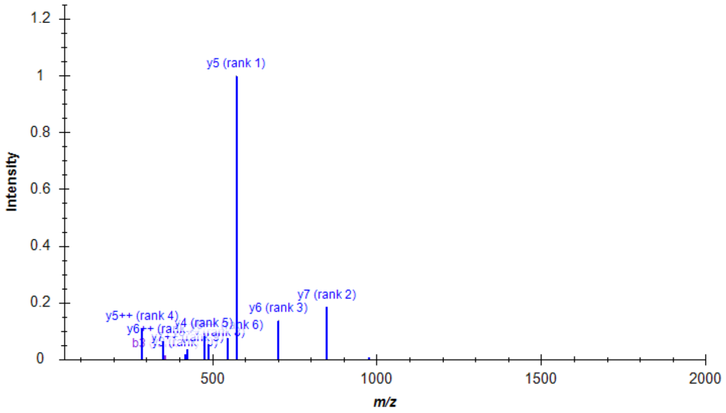

GOLGA8M | VAGELQAQVK (2)

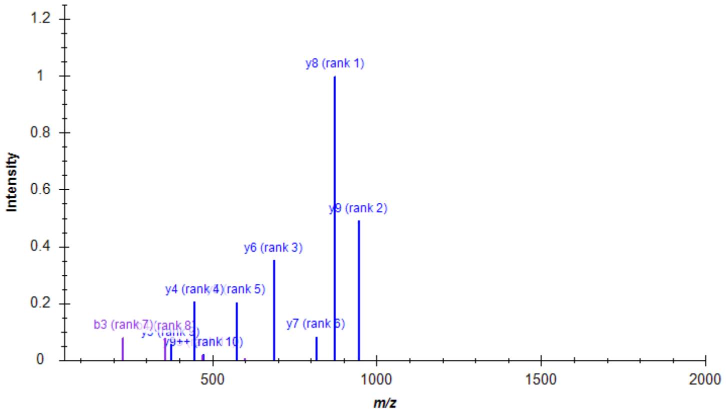

GRIK4 YQTYQRMWNYMYSK (4)

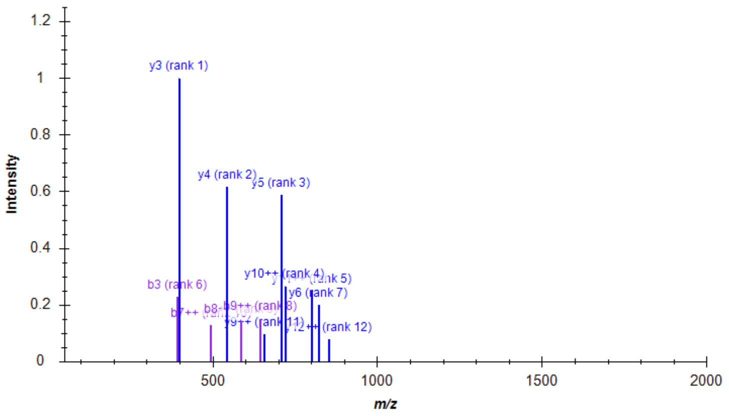

H2AC21 AGLQFPVGR (2)

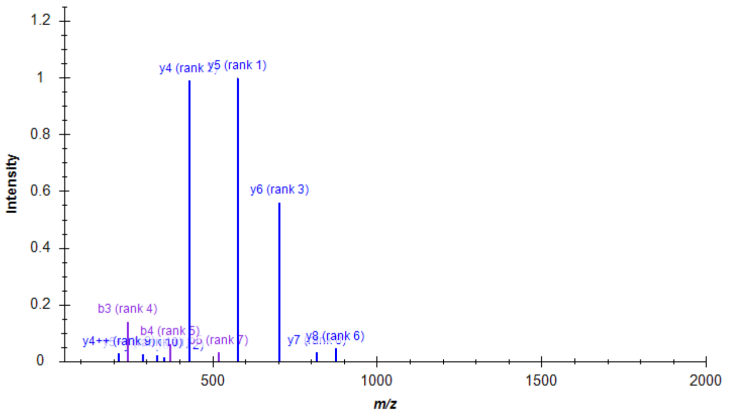

HIBADH DLGLAQDSATSTK (2)

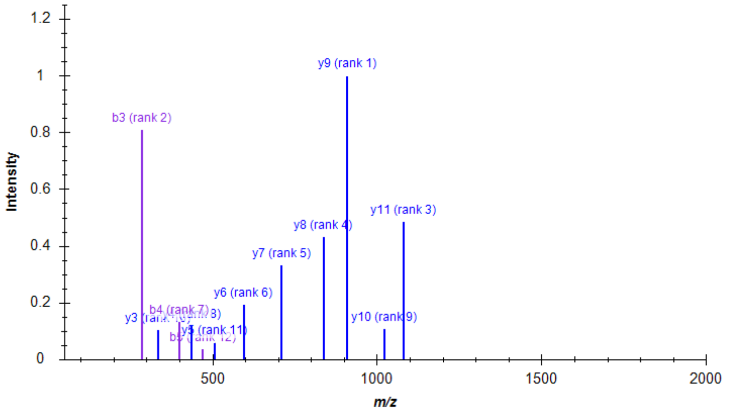

HLA-DPA1	TDYSFHK (2)	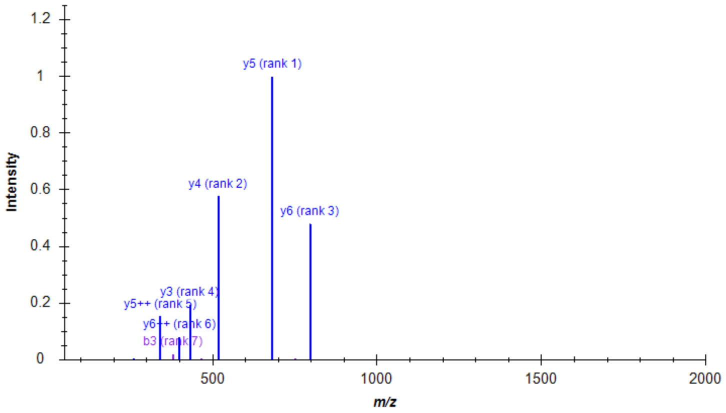
HRH1	SHSRQYVSGLHMNR (4)	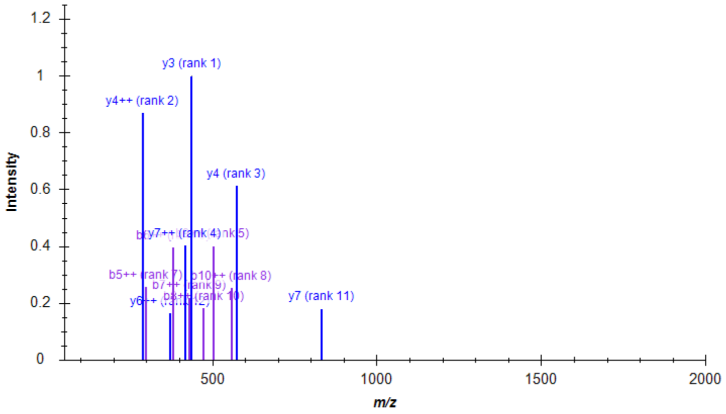
HS1BP3	SPGAAGLTSR (2)	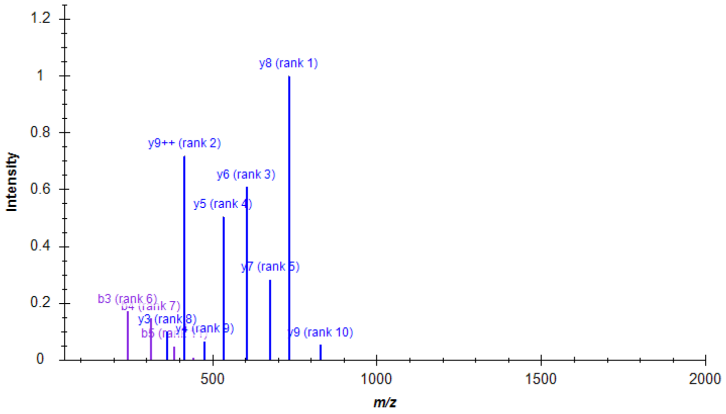

HSDL1

ALQYEYASK (2)

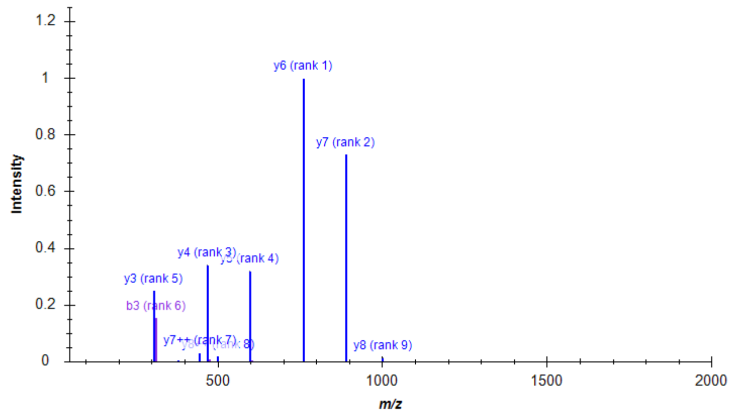

IL15

MRISKPHLR (3)

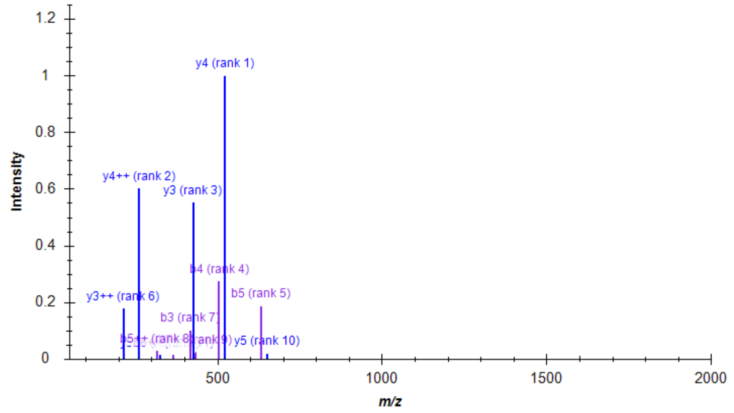

IL18R1

LLLENNK (2)

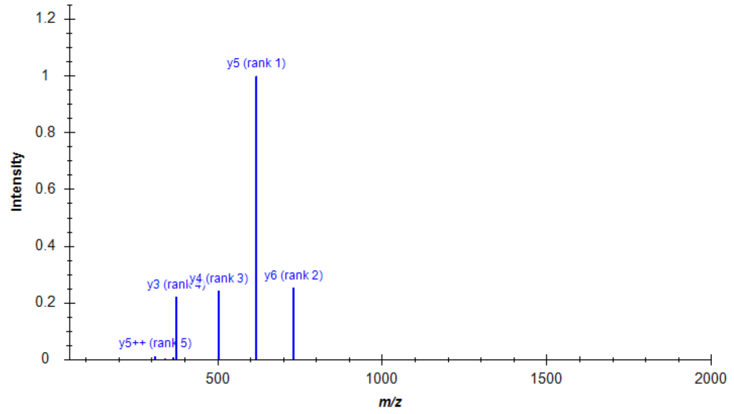

KCNA2	RMRYFDPLR (3)	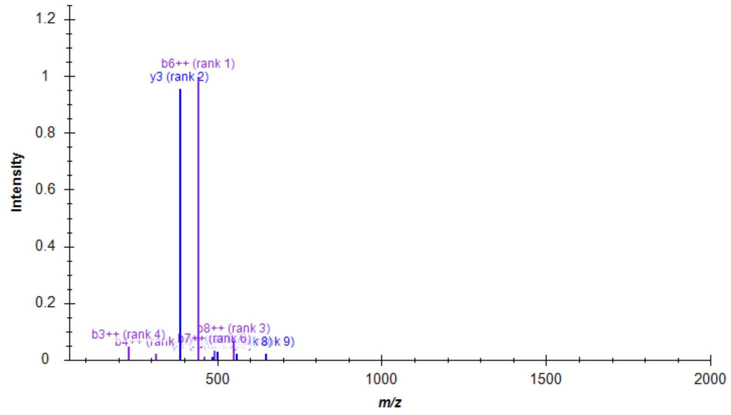
LHFPL5	RMCGEQTGKYTLGHCTIR (3)	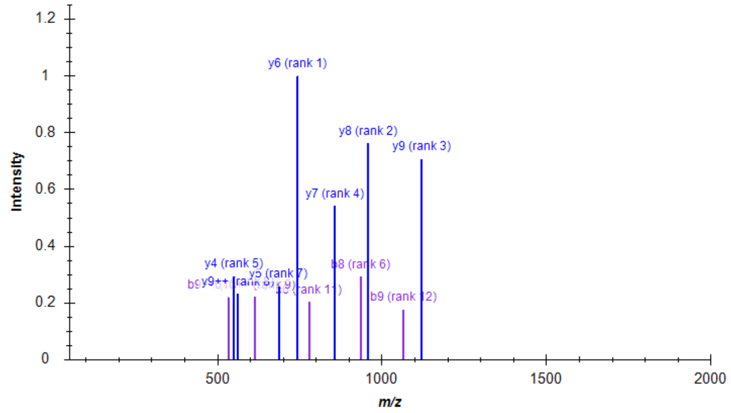
MARK4	MSSRTVLAPGNDR (3)	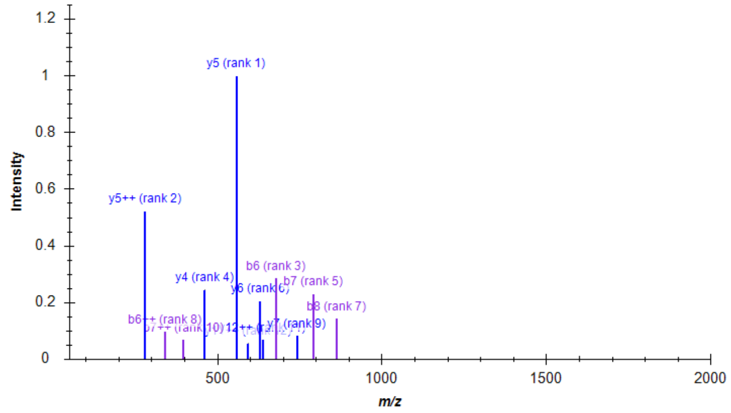

MBOAT1	RAEDLSAEQHR (3)	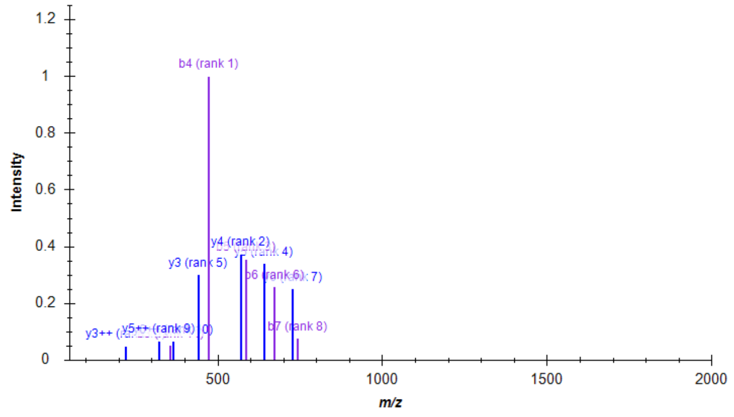
METTL24	MARERPPGR (3)	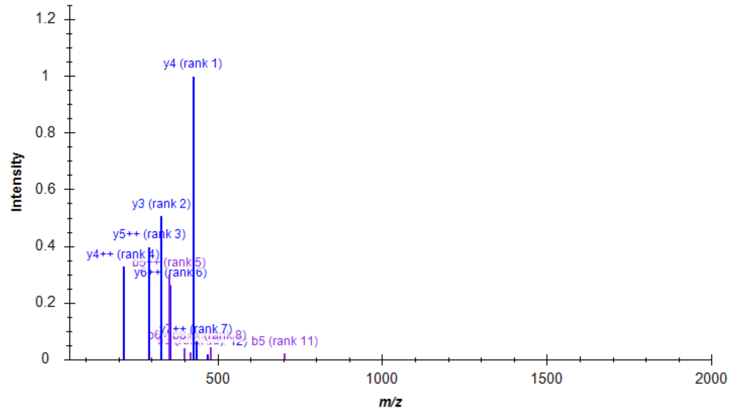
MIER2	RWHEMAGPQLPEGEAVK (4)	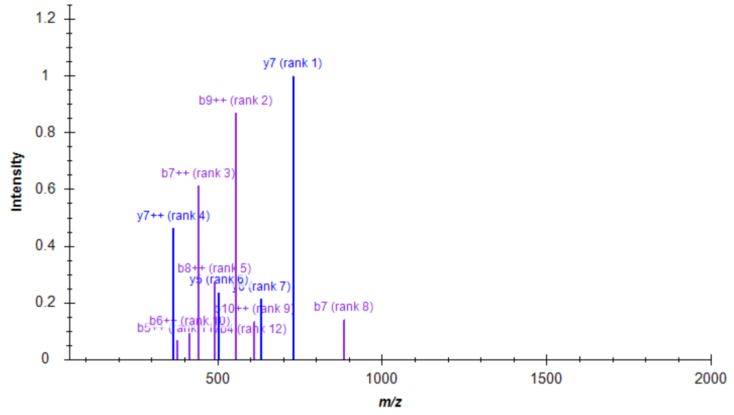

MLLT6	TCYICEEQGR (2)	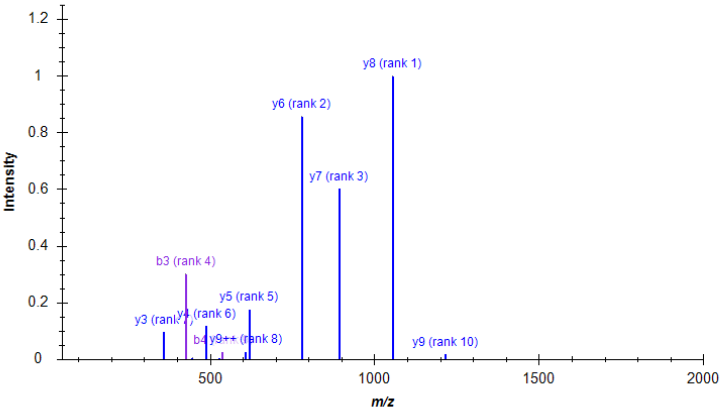
MND1	YKDCDPQVVEEIR (2)	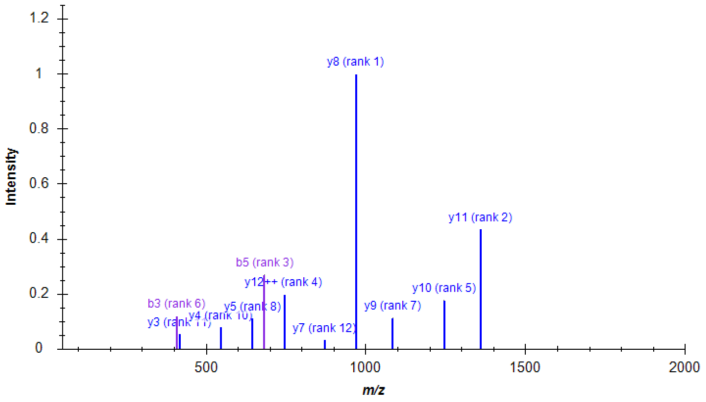
MRPL50	EKEPVVVETVEEK (3)	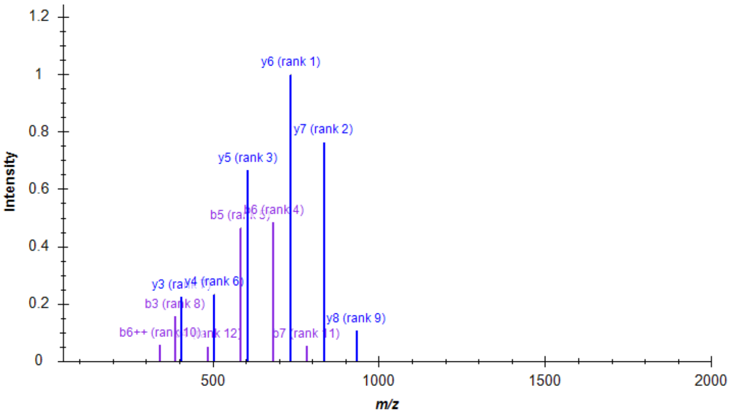

MRPL54 TLEELDPESR (2)

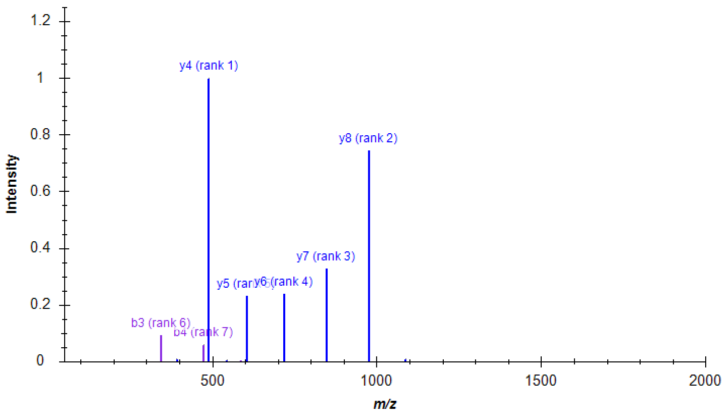

MRPS18C HITGLCGK (2)

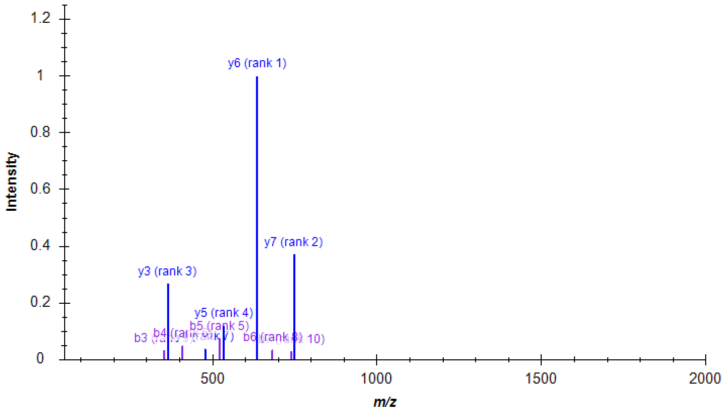

MSANTD2 APGPAMYER (2)

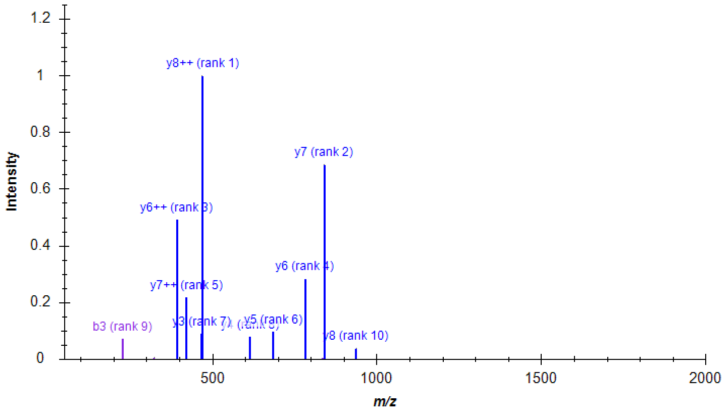

MSI2 VLGQPHHELDSK (3)

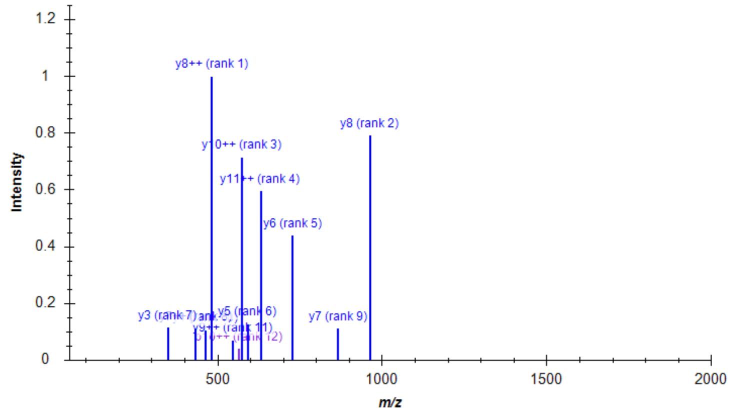

MTARC1 TPTTNAVHK (2)

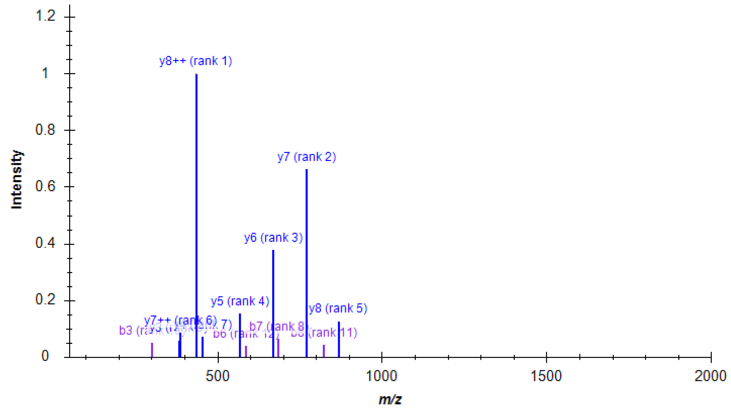

NAAA AAMAQVIGDR (2)

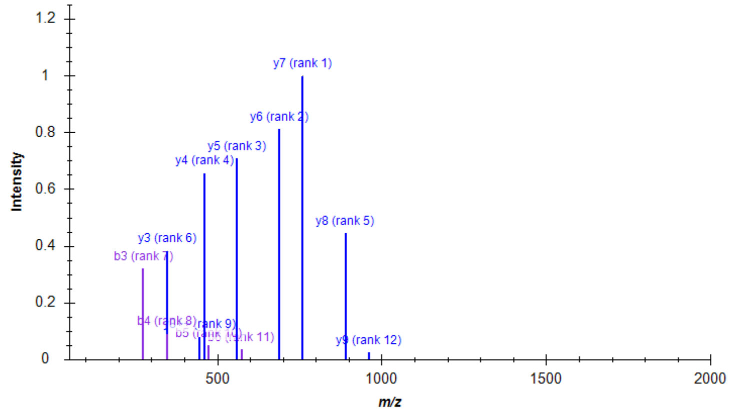

NDFIP2	AAAMAAAAAETSQR (2)	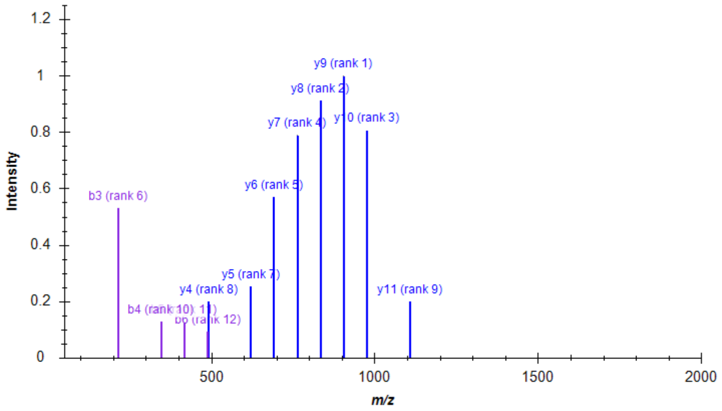
NDRG3	MADCGGLPQVVWPGK (2)	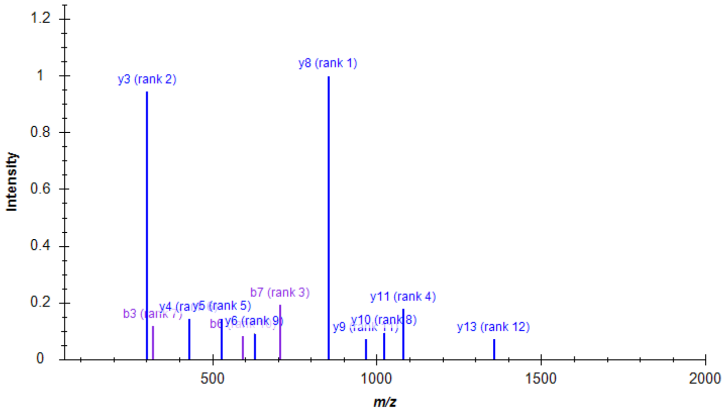
NMNAT1	HNLYSSESEDR (2)	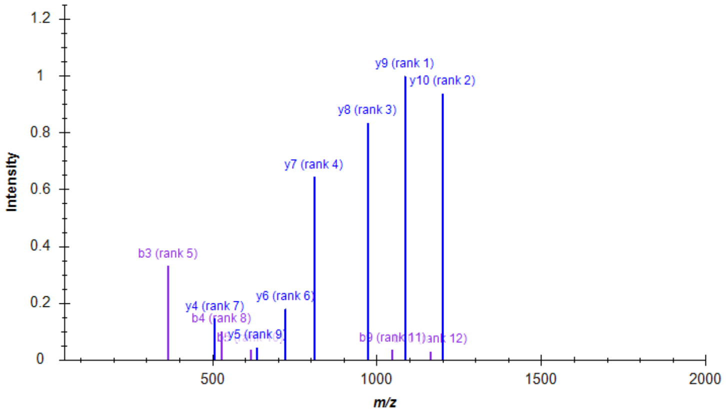

NR1H2	FPRMLMKLVSLR (4)	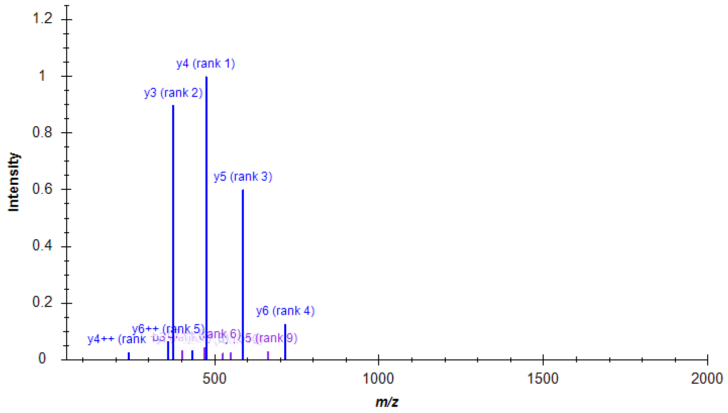
NUTM2G	AMREWQHTSNFDR (3)	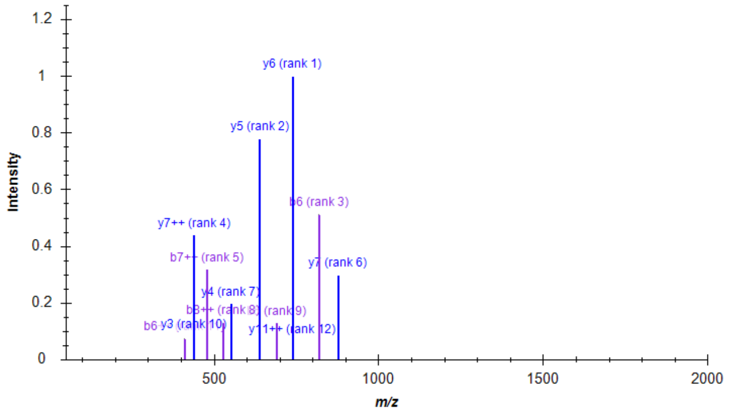
PATZ1	HSTEMLHNLNQQR (3)	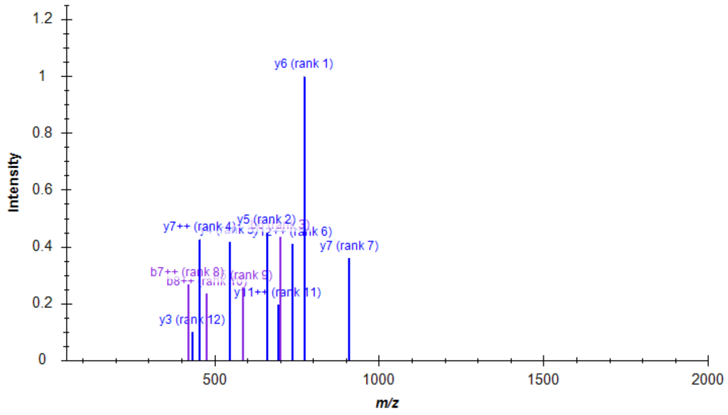

PCBP4	MLMHGKEVGSIIGK (4)	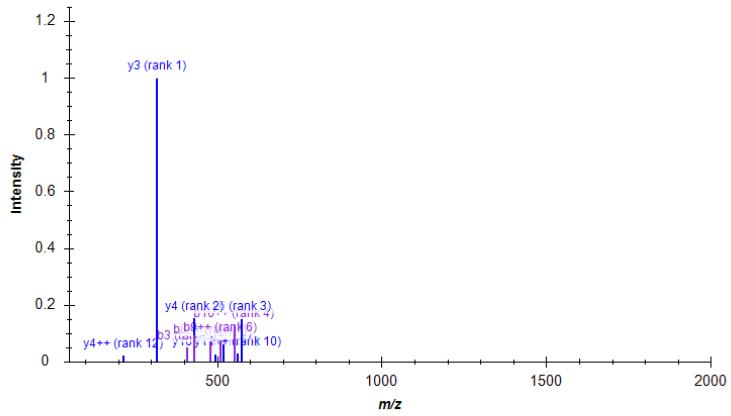
PDRG1	LFEAQGKPELK (3)	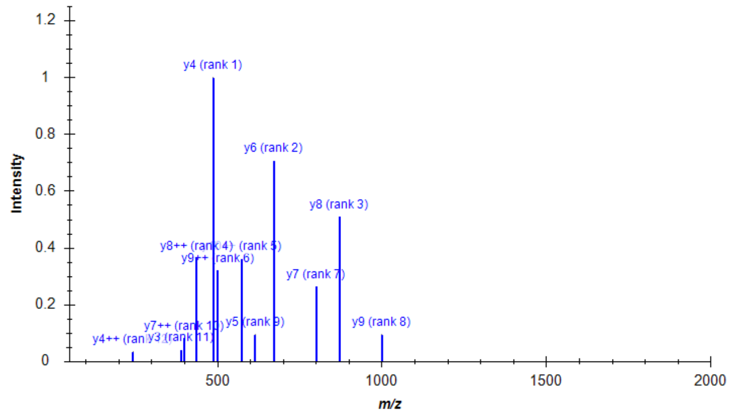
PGAP4	LQHYINPEPMR (3)	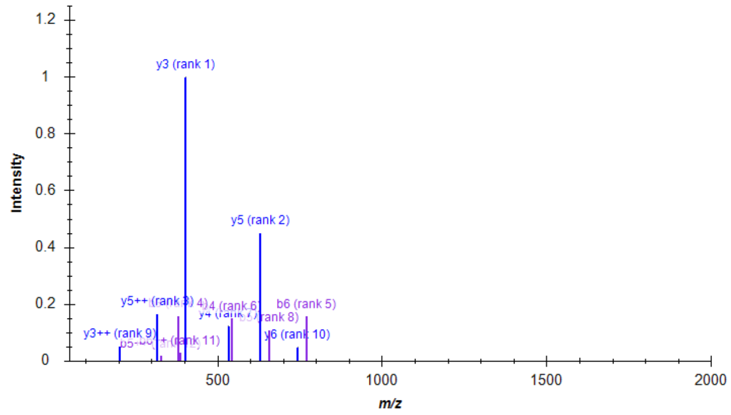

PLEKHG4B | ENPQRTEEMVQDFR (4)

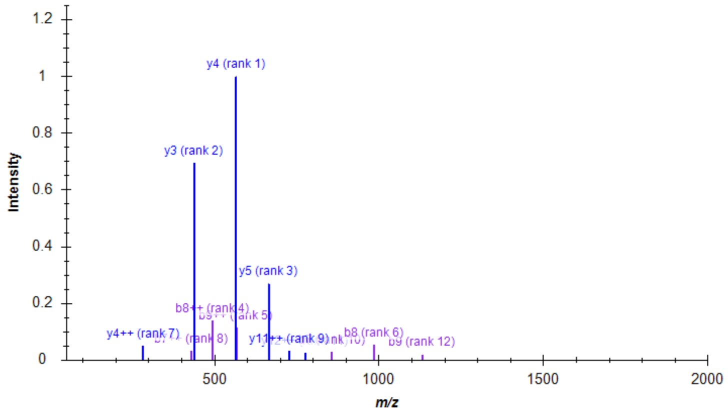

PPP1R11 | KVEWTSDTVDNEHMGR (3)

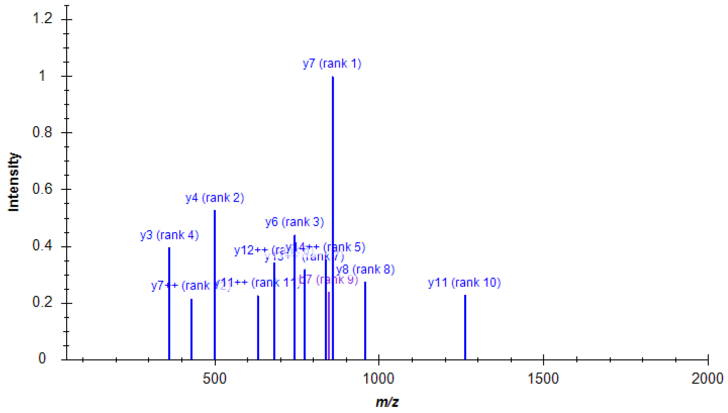

QRFPR | MKWQYTNR (3)

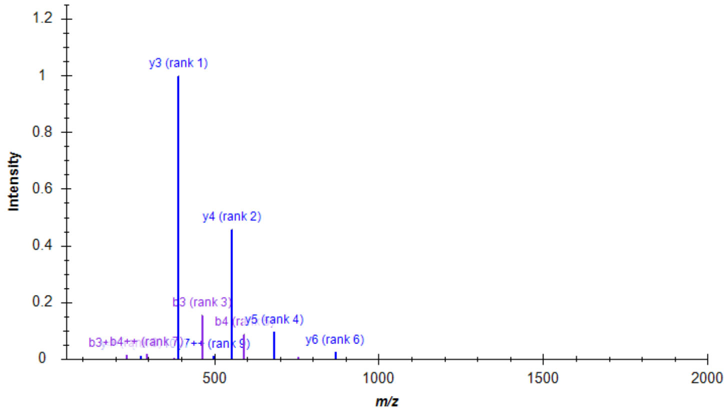

RFLNB	AVYTTTLDYNCR (2)	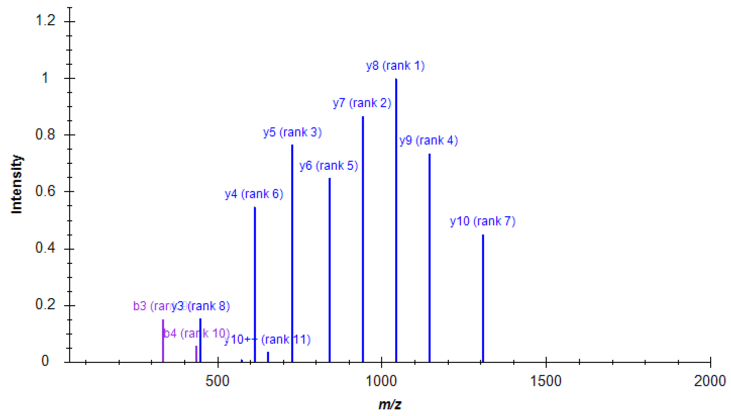
RGS10	TEEEEEDLPDAQTAAK (2)	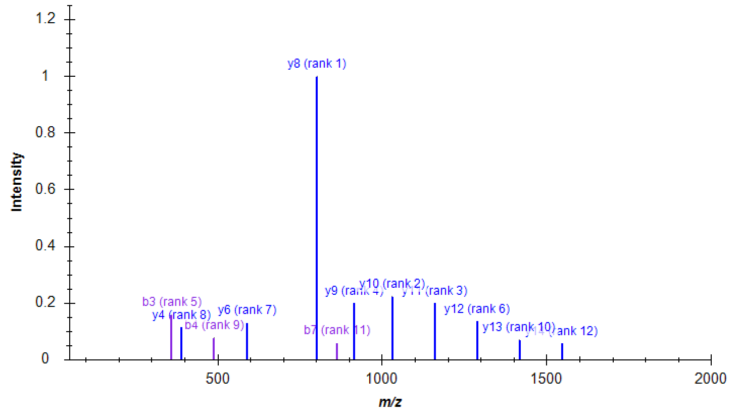
RNF133	MHLLKVGTWR (3)	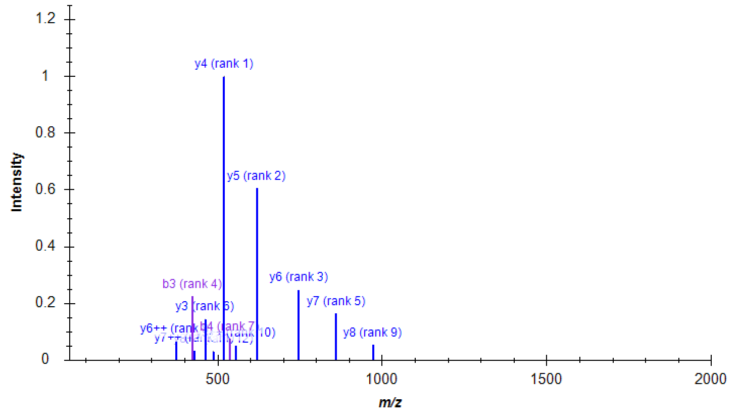

RNF7 — VQVMDACLR (2)

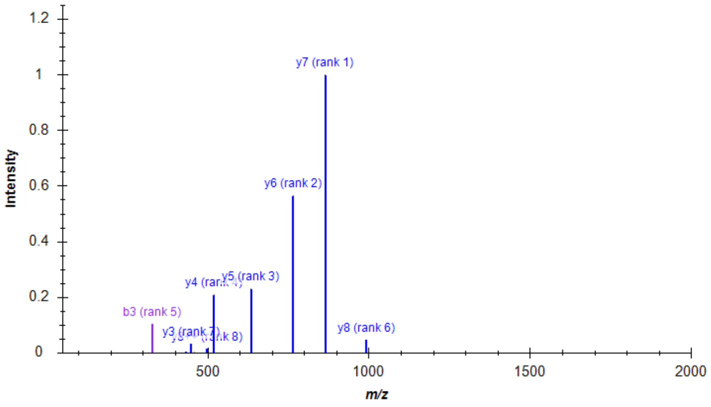

RPLP1 — AAGVNEPFWPGLFAK (2)

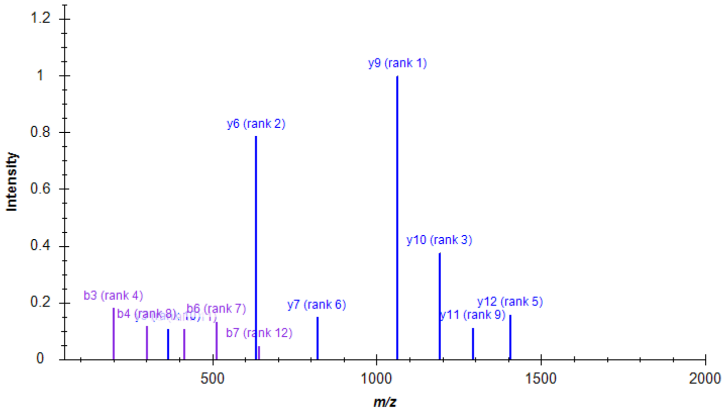

SELENOH — NAAALSQALR (2)

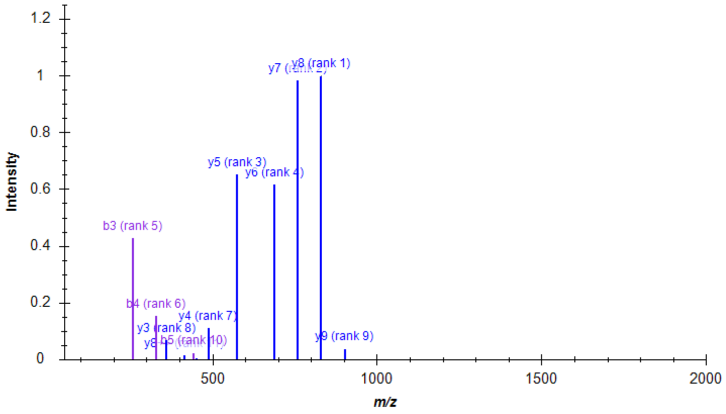

SLC10A4 | AENIIMMETAQTSL (2)

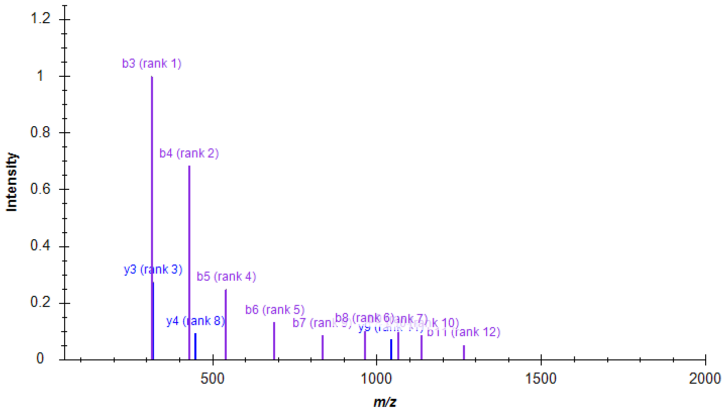

SLC35F6 | GRPLAEESEQER (3)

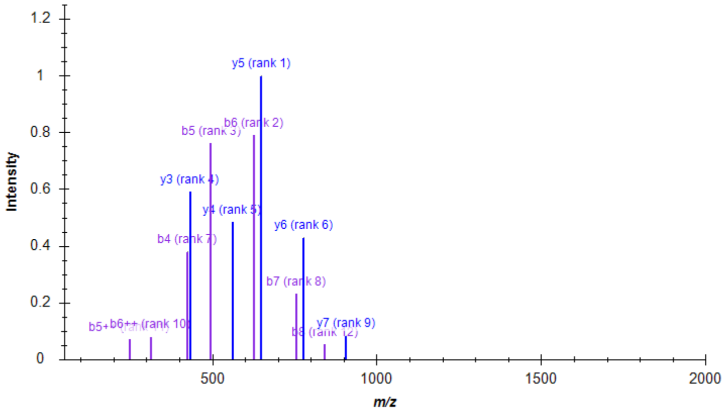

SMAD3 | KDEVCVNPYHYQR (3)

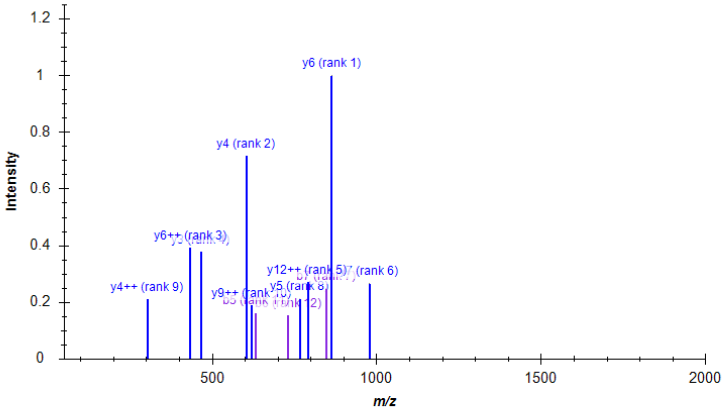

SMIM1	MQPQESHVHYSR (3)	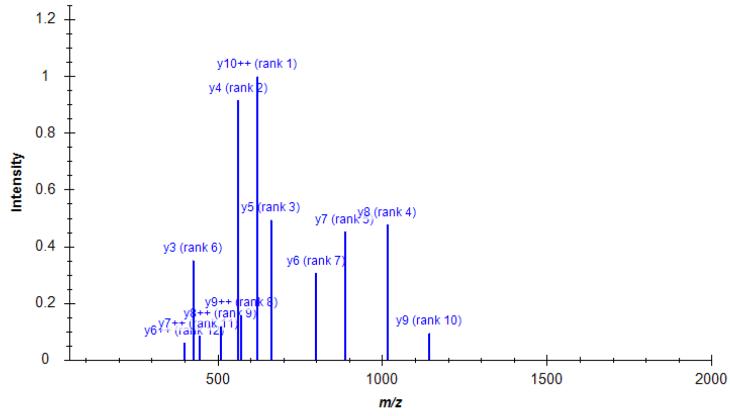
SP9	VHTTAADGLYPR (2)	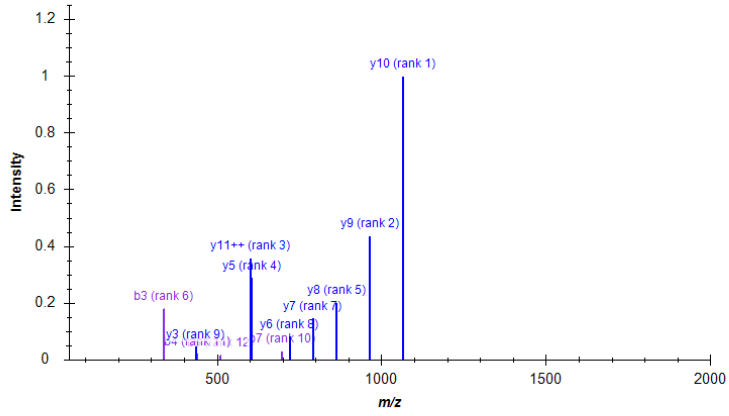
SSR3	KLSEADNR (2)	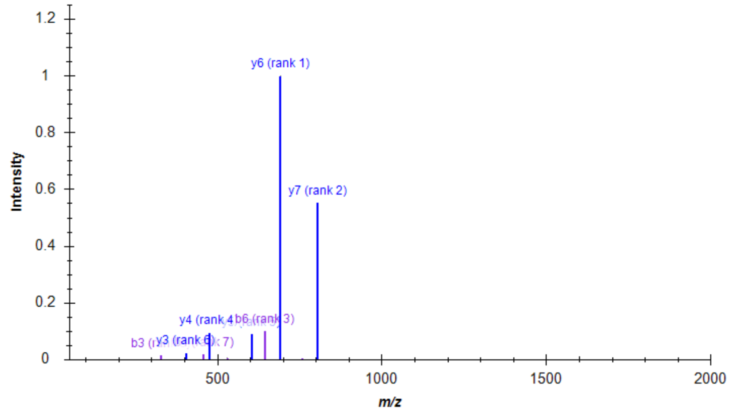

SUSD2	AGTWLAVHPNKVSMMEK (4)	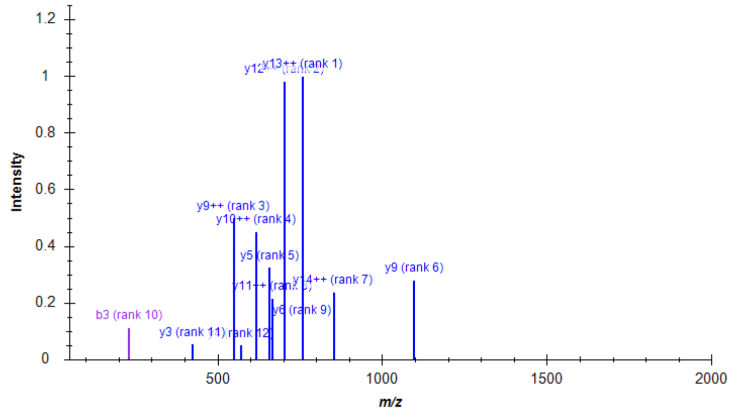
SYNGR1	DNPLNEGTDAAR (2)	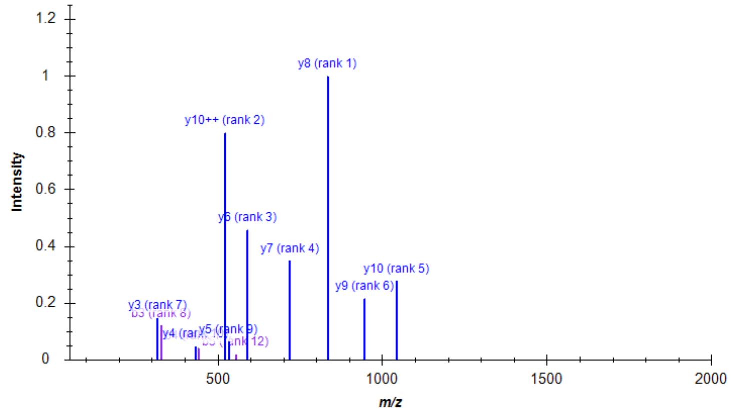
TAB2	TSSTSSSVNSQTLNR (2)	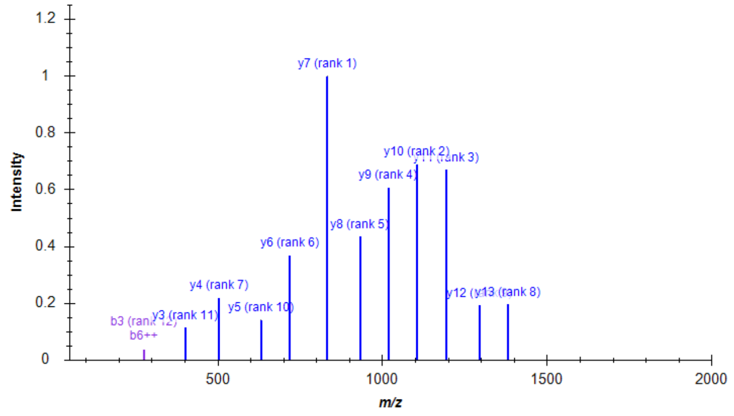

TAF9B	ADQSFTSPPPR (2)	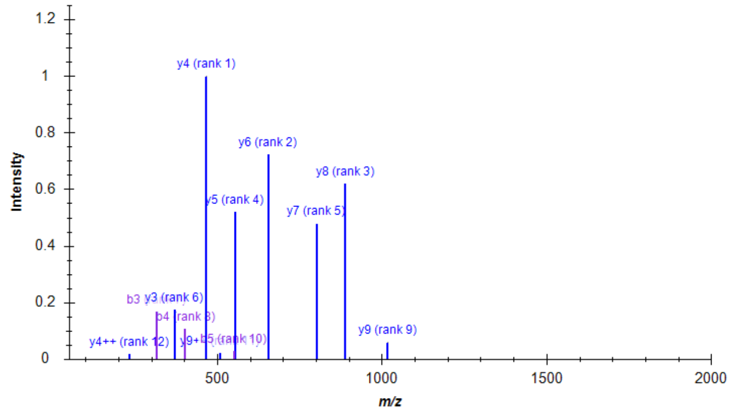
TGIF1	MVLAQSR (2)	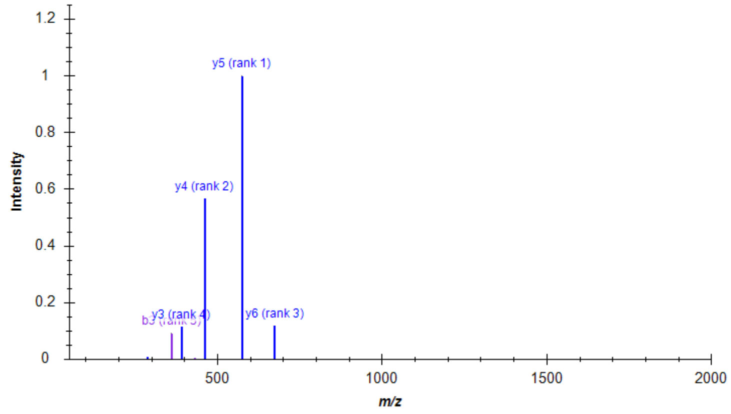
TIAL1	TLYVGNLSR (2)	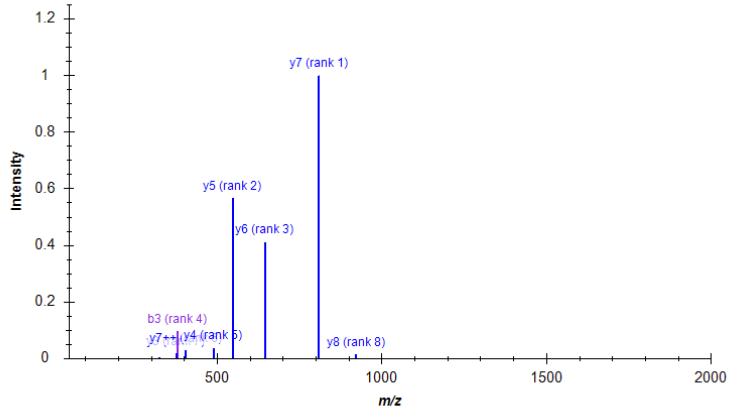

TSPAN14	VVNTQCGYDVR (2)	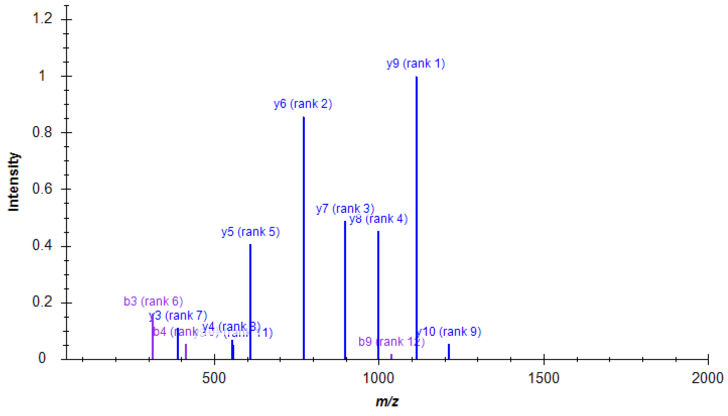
TUBA4A	QLFHPEQLITGK (2)	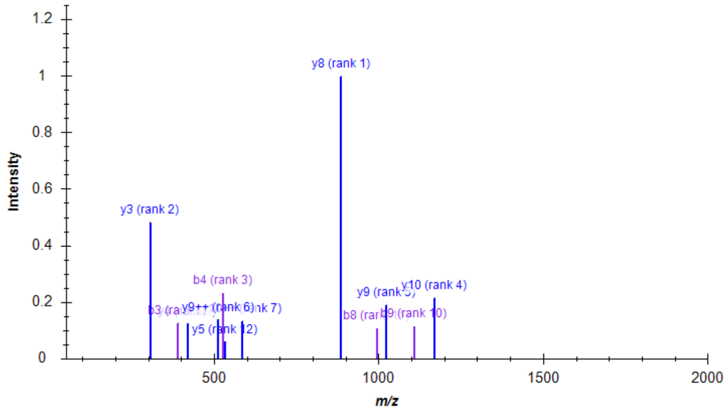
UBE2E2	ESVQQEPEREQVQPK (3)	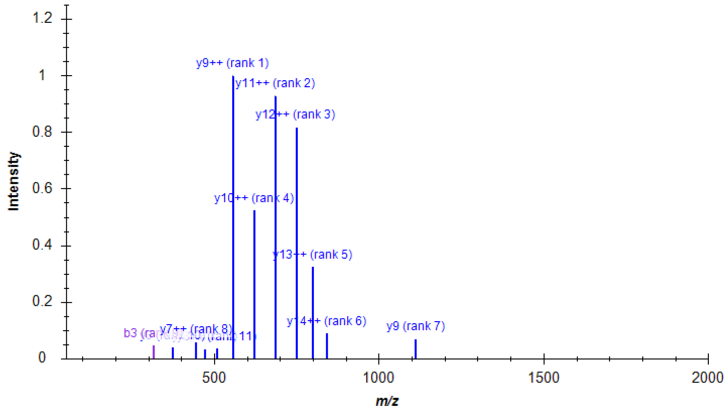

UBE2E3 DPAAPEPEEQEER (2)

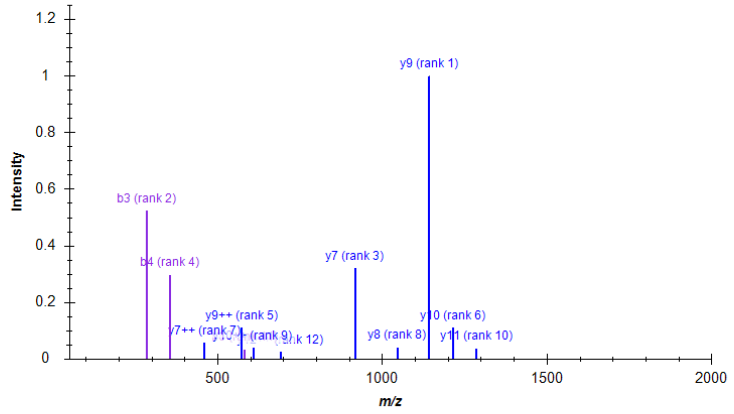

UBQLN3 NPAMMQEMIR (3)

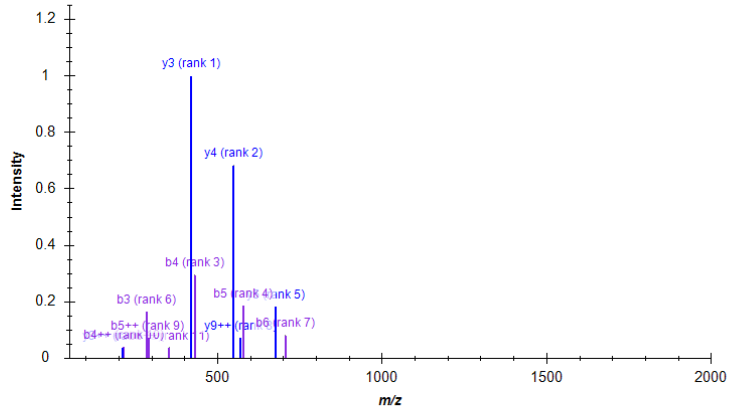

UCK2 LFVDTDADTR (2)

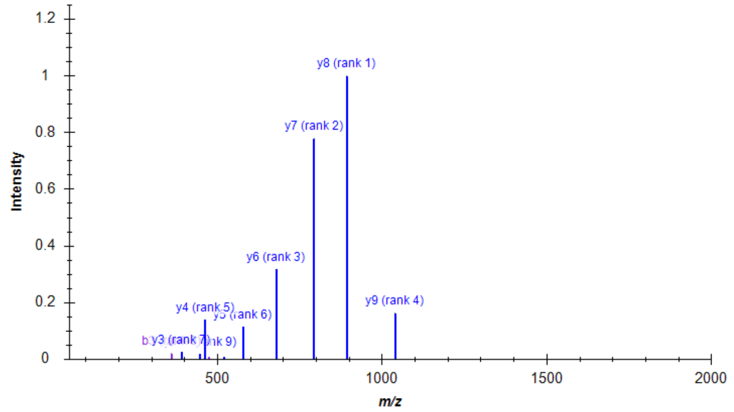

UGT1A5	MAIMNNMSLIIHR (3)	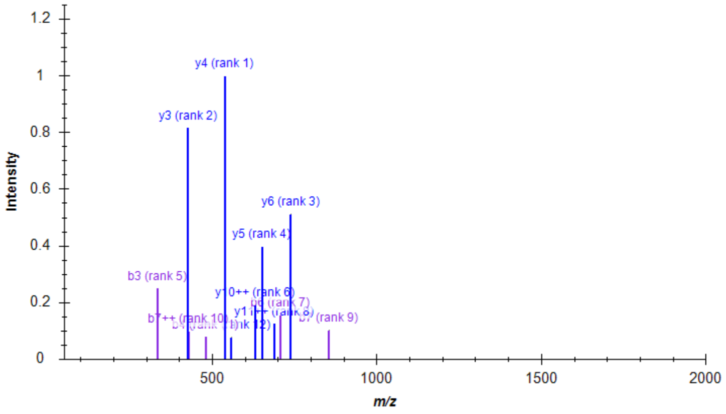
UXT	DKVYEQLAK (2)	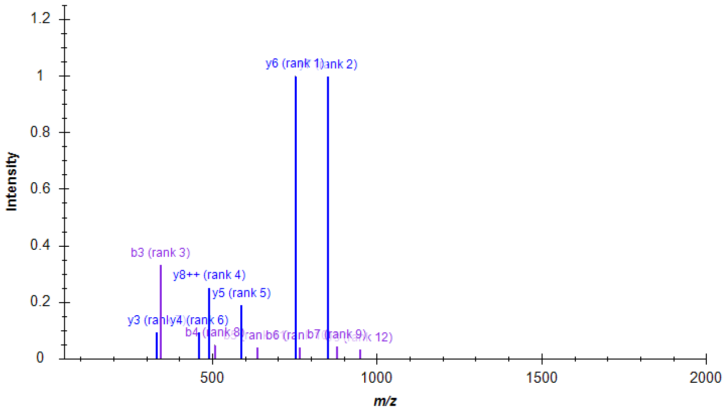
XAGE1B	VGILHLGSR (2)	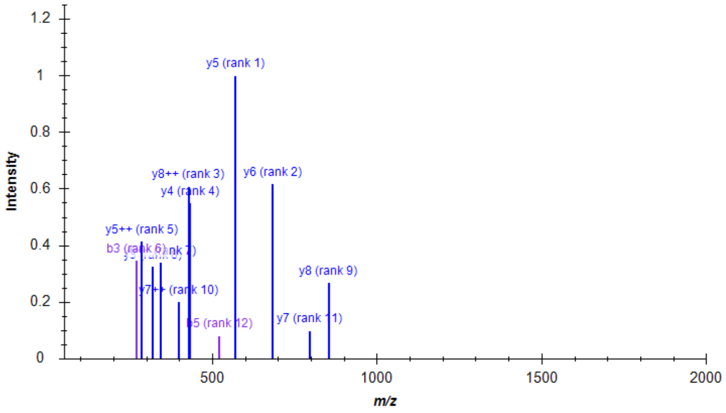

YIPF5 QYAGYDYSQQGR (2)

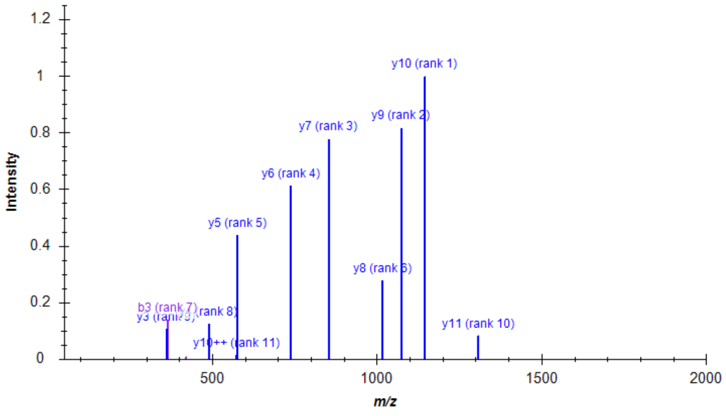

ZFAND6 TNGMCSVCYK (3)

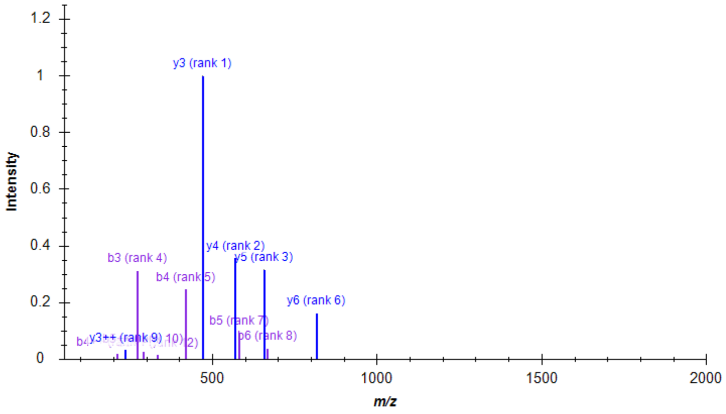

ZFP57 MHLGERPFCCTLCDK (3)

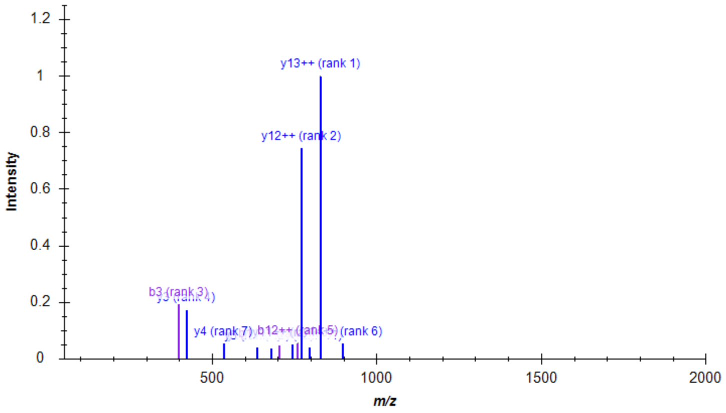

ZWILCH	LNCAAEDFYSR (2)	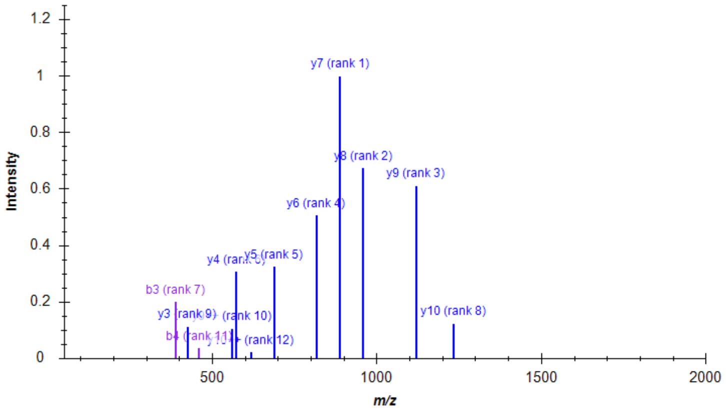
